# School-based socio-emotional learning programs to prevent depression, anxiety and suicide among adolescents: a global cost-effectiveness analysis

**DOI:** 10.1017/S204579602300029X

**Published:** 2023-07-12

**Authors:** Y. Y. Lee, S. Skeen, G. J. Melendez-Torres, C. A. Laurenzi, M. van Ommeren, A. Fleischmann, C. Servili, C. Mihalopoulos, D. Chisholm

**Affiliations:** 1 Monash University Health Economics Group, School of Public Health and Preventive Medicine, Monash University, Melbourne, Australia; 2 School of Public Health, Faculty of Medicine, The University of Queensland, Brisbane, Australia; 3 Mental Health Evaluation Research Stream, Queensland Centre for Mental Health Research, Brisbane, Australia; 4 Institute for Life Course Health Research, Department of Global Health, Stellenbosch University, Stellenbosch, South Africa; 5 Amsterdam Institute for Social Science Research, Faculty of Social and Behavioural Sciences, University of Amsterdam, Amsterdam, The Netherlands; 6 College of Medicine and Health, University of Exeter, Exeter, UK; 7 Department of Mental Health and Substance Use, World Health Organization, Geneva, Switzerland

**Keywords:** adolescent, anxiety disorders, cost-effectiveness analysis, depressive disorder, economic model, prevention, school health services, suicide

## Abstract

**Aims:**

Preventing the occurrence of depression/anxiety and suicide during adolescence can lead to substantive health gains over the course of an individual person’s life. This study set out to identify the expected population-level costs and health impacts of implementing universal and indicated school-based socio-emotional learning (SEL) programs in different country contexts.

**Methods:**

A Markov model was developed to examine the effectiveness of delivering universal and indicated school-based SEL programs to prevent the onset of depression/anxiety and suicide deaths among adolescents. Intervention health impacts were measured in healthy life years gained (HLYGs) over a 100-year time horizon. Country-specific intervention costs were calculated and denominated in 2017 international dollars (2017 I$) under a health systems perspective. Cost-effectiveness findings were subsequently expressed in terms of I$ per HLYG. Analyses were conducted on a group of 20 countries from different regions and income levels, with final results aggregated and presented by country income group – that is, low and lower middle income countries (LLMICs) and upper middle and high-income countries (UMHICs). Uncertainty and sensitivity analyses were conducted to test model assumptions.

**Results:**

Implementation costs ranged from an annual per capita investment of I$0.10 in LLMICs to I$0.16 in UMHICs for the universal SEL program and I$0.06 in LLMICs to I$0.09 in UMHICs for the indicated SEL program. The universal SEL program generated 100 HLYGs per 1 million population compared to 5 for the indicated SEL program in LLMICs. The cost per HLYG was I$958 in LLMICS and I$2,006 in UMHICs for the universal SEL program and I$11,123 in LLMICs and I$18,473 in UMHICs for the indicated SEL program. Cost-effectiveness findings were highly sensitive to variations around input parameter values involving the intervention effect sizes and the disability weight used to estimate HLYGs.

**Conclusions:**

The results of this analysis suggest that universal and indicated SEL programs require a low level of investment (in the range of I$0.05 to I$0.20 per head of population) but that universal SEL programs produce significantly greater health benefits at a population level and therefore better value for money (e.g., less than I$1,000 per HLYG in LLMICs). Despite producing fewer population-level health benefits, the implementation of indicated SEL programs may be justified as a means of reducing population inequalities that affect high-risk populations who would benefit from a more tailored intervention approach.

## Introduction

The initial onset of common mental health conditions, such as depression and/or anxiety, often occurs during adolescence between the ages of 10 and 19 years (Solmi *et al.*, 2022). Additionally, the rate of suicide deaths rapidly increases during adolescence and is the fourth leading cause of death among young people, with 88% of adolescent suicides occurring in low- and middle-income countries (WHO, 2021c). Important mental health risk factors at this age include adverse experiences (e.g., family violence, bullying) (Patton *et al.*, 2016); alcohol and substance use (Esmaeelzadeh *et al.*, 2018); and as highlighted during the COVID-19 pandemic, isolation from social networks and activities (Munasinghe *et al.*, 2020). Preventing the occurrence of depression/anxiety and suicidal behaviours during this crucial developmental stage can lead to substantive health gains throughout a person’s life (de Girolamo *et al.*, 2012).

School settings are an important platform for delivering mental health prevention/promotion interventions among adolescents (Fazel *et al.*, 2014a, 2014b). School-based interventions to prevent mental health conditions and/or suicide typically involve a trained facilitator (e.g., teacher or health professional) delivering several intervention modules that teach young people psychological strategies to improve their mental well-being and reduce the risk of adverse mental health outcomes. Current evidence indicates that school-based socio-emotional learning (SEL) programs are effective in reducing the incidence of depression/anxiety and suicidal behaviours among adolescent students (Hetrick *et al.*, 2016; Skeen *et al.*, 2019; Wasserman *et al.*, 2015). These programs have been included in recent guidelines published by the World Health Organization (WHO) on mental health promotion and preventive interventions for adolescents (WHO, 2020) and the prevention of suicide (WHO, 2021a, 2022). Two categories of school-based SEL programs include (1) universal interventions, which target all students regardless of their underlying risk profile, and (2) indicated interventions, which target students with an elevated risk of depression, anxiety and/or suicide completion (Mrazek and Haggerty, 1994). Students eligible for an indicated intervention are identified (via individual screening that involves scoring a checklist of mental health symptoms or indicators of suicide risk) as having a subthreshold mental health condition – that is, clinically relevant symptoms that fall short of diagnostic criteria for mental illness. Indicated SEL interventions often produce larger intervention effect sizes than universal SEL interventions (Hetrick *et al.*, 2016). However, this increased efficacy is offset by the large costs involved in screening for subthreshold cases and the smaller aggregate health impacts, accruing to a narrow subset of the adolescent population (Lee *et al.*, 2017).

In May 2019, the 72nd World Health Assembly passed a resolution requesting the WHO Director-General to prepare and update a menu of policy options and cost-effective interventions for improving mental health (WHO, 2021d). This menu of policy options has informed an update of the WHO comprehensive mental health action plan 2013–2020 (WHO, 2013). This study presents the results of an economic evaluation undertaken as part of the aforementioned update to examine the cost-effectiveness of delivering universal and indicated school-based SEL programs to prevent depression/anxiety and suicidal behaviours, among adolescent students across different country contexts. This study contributes to the ongoing generation of comparative economic evidence for population-wide, as well as clinical-level, mental health interventions (WHO, 2021d). In the context of recent COVID-19 pandemic response and recovery efforts, it also addresses urgent policy questions around how, where and ‘at what cost’ countries can roll out enhanced levels of mental health awareness and support to affected adolescent populations.

## Methods

### Analytic approach

This study adopted a health systems perspective in accordance with the World Health Organization CHOosing Interventions that are Cost-Effective (WHO-CHOICE) methods (Bertram *et al.*, 2021a). WHO-CHOICE is a program of work that helps countries to identify healthcare priorities based on health impacts and cost-effectiveness (Bertram and Edejer, 2021). All healthcare options are compared to a common comparator, a null scenario in which the impacts of currently implemented interventions are removed, thereby enabling comparison of interventions across geographical areas and domains of health. Analytic choices arising from the implementation of WHO-CHOICE methods are briefly presented here, with detailed methods provided in Appendix S1. A Markov model was developed in Microsoft Excel 2019 to examine the costs and health impacts of scaling up the delivery of universal and indicated school-based SEL programs. Universal and indicated SEL programs comprised the delivery of several SEL program components (or intervention modules) that are effective in preventing the onset of depression/anxiety and suicidal behaviour (Skeen *et al.*, 2019; Wasserman *et al.*, 2015). Delivery of the universal SEL program encompassed all secondary school students aged 12–17 years, while delivery of the indicated SEL program targeted at-risk school students identified as having subthreshold depression/anxiety following screening. Analyses were conducted on a group of 20 countries from different regions and income levels (that between them account for >80% of the global population and global burden of mental health conditions) (WHO, 2021d). Aggregated results are presented for two country income groups: low- to lower middle-income countries (LLMICs) and upper middle- to high-income countries (UMHICs). Table 1 lists the 20 countries by country income group, alongside other relevant data.

**Table 1.** Adolescent population (aged 12–17 years), secondary school attendance and selected epidemiological parameters across 20 countries

Country	2017 population aged 12−17 years (thousands)	Secondary school attendance (%)	Depression prevalence 12−17 years (%)	Anxiety disorders prevalence 12−17 years (%)	Suicide rate 12−17 years (per 100,000)
*Low- to lower middle-income countries (LLMICs)*					
Bangladesh	Male: 9,988	Male: 70.2	Male: 1.1	Male: 3.2	Male: 4.3
	Female: 9,552	Female: 78.1	Female: 2.0	Female: 5.0	Female: 8.0
	Both: 19,540	Both: 74.1	Both: 1.5	Both: 4.1	Both: 6.1
Ethiopia	Male: 7,640	Male: 46.5	Male: 1.6	Male: 2.8	Male: 2.7
	Female: 7,528	Female: 42.7	Female: 1.9	Female: 5.3	Female: 1.5
	Both: 15,168	Both: 44.7	Both: 1.8	Both: 4.0	Both: 2.1
Guatemala	Male: 1,204	Male: 57.2	Male: 1.1	Male: 2.1	Male: 4.2
	Female: 1,156	Female: 51.9	Female: 1.9	Female: 3.6	Female: 3.7
	Both: 2,360	Both: 54.6	Both: 1.5	Both: 2.9	Both: 4.0
India	Male: 80,340	Male: 65.9	Male: 0.9	Male: 2.6	Male: 5.0
	Female: 71,963	Female: 67.3	Female: 1.2	Female: 3.8	Female: 10.7
	Both: 152,302	Both: 66.6	Both: 1.0	Both: 3.2	Both: 7.7
Indonesia	Male: 14,153	Male: 79.5	Male: 0.9	Male: 2.5	Male: 2.4
	Female: 13,497	Female: 78.7	Female: 1.1	Female: 3.8	Female: 1.0
	Both: 27,650	Both: 79.1	Both: 1.0	Both: 3.1	Both: 1.7
Nigeria	Male: 13,076	Male: 68.3	Male: 1.3	Male: 2.9	Male: 2.2
	Female: 12,604	Female: 68.0	Female: 2.1	Female: 4.2	Female: .9.0
	Both: 25,680	Both: 68.2	Both: 1.7	Both: 3.5	Both: 1.6
Pakistan	Male: 12,159	Male: 49.4	Male: 0.8	Male: 2.9	Male: 2.3
	Female: 11,240	Female: 40.1	Female: 1.4	Female: 4.4	Female: 5.9
	Both: 23,398	Both: 45.0	Both: 1.1	Both: 3.6	Both: 4.1
Philippines	Male: 6,556	Male: 88.0	Male: 1.1	Male: 2.5	Male: 3.7
	Female: 6,131	Female: 92.9	Female: 1.3	Female: 3.9	Female: 1.9
	Both: 12,687	Both: 90.4	Both: 1.2	Both: 3.1	Both: 2.8
Ukraine	Male: 1,231	Male: 95.8	Male: 1.3	Male: 2.5	Male: 17.0
	Female: 1,162	Female: 97.1	Female: 1.5	Female: 3.5	Female: 5.0
	Both: 2,393	Both: 96.5	Both: 1.4	Both: 3.0	Both: 11.2
Viet Nam	Male: 4,151	Male: 68.3	Male: 0.8	Male: 1.5	Male: 3.1
	Female: 3,906	Female: 68.0	Female: 1.5	Female: 2.6	Female: 1.7
	Both: 8,058	Both: 68.2	Both: 1.2	Both: 2.0	Both: 2.4
*Upper middle- to high-income countries (UMHICs)*					
China	Male: 51,575	Male: 79.7	Male: 0.8	Male: 2.6	Male: 2.2
	Female: 44,726	Female: 81.2	Female: 1.1	Female: 4.1	Female: 1.7
	Both: 96,301	Both: 80.4	Both: 0.9	Both: 3.3	Both: 2.0
Germany	Male: 2,270	Male: 95.8	Male: 1.1	Male: 5.1	Male: 3.9
	Female: 2,155	Female: 96.5	Female: 2.1	Female: 10.0	Female: 1.6
	Both: 4,425	Both: 96.2	Both: 1.6	Both: 7.4	Both: 2.8
Iran (Islamic Republic of)	Male: 3,365	Male: 85.8	Male: 2.2	Male: 6.3	Male: 5.1
	Female: 3,126	Female: 87.7	Female: 2.8	Female: 7.1	Female: 3.3
	Both: 6,491	Both: 86.7	Both: 2.5	Both: 6.7	Both: 4.2
Japan	Male: 3,559	Male: 97.8	Male: 1.0	Male: 3.6	Male: 4.8
	Female: 3,374	Female: 99.0	Female: 2.0	Female: 4.6	Female: 2.3
	Both: 6,932	Both: 98.4	Both: 1.5	Both: 4.1	Both: 3.6
Mexico	Male: 7,296	Male: 79.0	Male: 0.8	Male: 2.1	Male: 6.1
	Female: 7,021	Female: 81.9	Female: 1.4	Female: 3.9	Female: 3.1
	Both: 14,317	Both: 80.4	Both: 1.1	Both: 3.0	Both: 4.6
Russian Federation	Male: 4,282	Male: 96.2	Male: 1.0	Male: 2.5	Male: 12.6
	Female: 4,085	Female: 97.3	Female: 1.3	Female: 3.5	Female: 4.2
	Both: 8,366	Both: 96.8	Both: 1.2	Both: 3.0	Both: 8.5
South Africa	Male: 3,231	Male: 96.2	Male: 1.5	Male: 3.4	Male: 2.9
	Female: 3,148	Female: 85.6	Female: 1.9	Female: 5.2	Female: 1.2
	Both: 6,379	Both: 91.0	Both: 1.7	Both: 4.3	Both: 2.0
Thailand	Male: 2,601	Male: 83.9	Male: 1.1	Male: 2.5	Male: 6.2
	Female: 2,521	Female: 82.7	Female: 1.4	Female: 3.8	Female: 1.8
	Both: 5,121	Both: 83.3	Both: 1.2	Both: 3.1	Both: 4.0
Turkey	Male: 4,074	Male: 87.8	Male: 1.5	Male: 3.6	Male: 3.2
	Female: 3,917	Female: 86.6	Female: 2.3	Female: 4.2	Female: 1.6
	Both: 7,991	Both: 87.2	Both: 1.8	Both: 3.9	Both: 2.4
United States of America	Male: 12,345	Male: 94.9	Male: 1.9	Male: 4.2	Male: 8.0
	Female: 11,864	Female: 96.1	Female: 4.8	Female: 7.0	Female: 2.7
	Both: 24,210	Both: 95.5	Both: 3.3	Both: 5.6	Both: 5.4

Country-specific costs were obtained from the WHO-CHOICE database and denominated in 2017 international dollars (2017 I$) (Bertram *et al.*, 2017). In accordance with WHO-CHOICE methods, all costs were discounted at a 3% annual rate, with no discounting applied to health impacts while healthcare cost savings and changes in workforce productivity were out of scope (Bertram *et al.*, 2021a). The model did not evaluate impacts on future employment outcomes that result from enhanced educational outcomes due to improved adolescent mental health. Intervention health impacts were measured in healthy life years gained (HLYGs). Costs and health impacts were analysed for each of the 20 countries at the country-specific level, with the final results aggregated and presented by country income group (i.e., LLMICs and UMHICs). The main study outcome was the average cost-effectiveness ratio, expressed as the total intervention cost (2017 I$) divided by the incremental HLYGs produced by the intervention when compared to no intervention.

The modelling approach was refined following consultations with an international expert panel who provided in-person feedback at a meeting held at WHO headquarters office in Geneva on August 21, 2019, and through out-of-session email communications (see Acknowledgements). This study adhered to Consolidated Health Economic Evaluation Reporting Standards (CHEERS) (see Appendix S2) (Husereau *et al.*, 2013).

### Demographic projections

**Figure 1.**
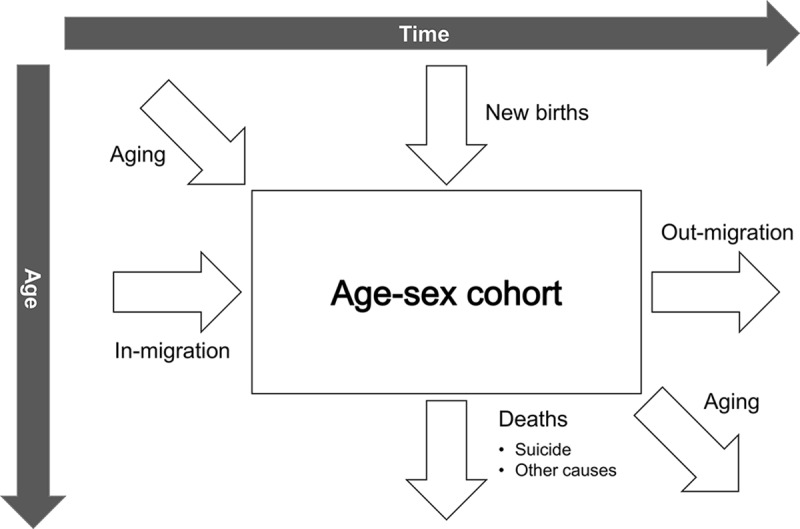
Overview of the demographic projection model.

The demographic projection model (Fig. 1) begins with the country population in the 2017 baseline year, split by 1-year age group and sex. Flows in and out of each age–sex cohort were simulated for each subsequent year over 100 years. These flows included outflows due to death (suicide or other causes), inflows in the 0- to 1-year age cohort due to new births, and net migration. Data on the 2017 country population between ages 0 and 80+ years and corresponding age-specific mortality rates over the 100-year time horizon were obtained from OneHealth Tool (Avenir Health, 2022). Data on new births and net migration were obtained from the UN World Population Prospects 2017 (WPP 2017) report (UN DESA, 2017). All demographic projections were validated against WPP 2017 estimates. See Appendix S3 for further details.

### Intervention coverage

Universal and indicated school-based SEL programs were modelled with a population coverage of 95% among adolescents aged 12–17 years. Country-specific adjustments were then made to exclude adolescents who do not attend secondary school (UIS, 2022). Under the indicated SEL program, intervention coverage was further restricted to students with subthreshold depression/anxiety – around 5% of adolescents (Bertha and Balazs, 2013; Carrellas *et al.*, 2017; Haller *et al.*, 2014). See Appendix S3 for further details.

### Intervention effect sizes

The model quantified the impact of universal and indicated school-based SEL programs in reducing the incidence of depression/anxiety and suicide mortality among adolescents. It was assumed that universal and indicated SEL programs encompassed a modular design, such that both programs incorporated evidence-based program components that are efficacious in reducing the risk of depression/anxiety and suicide deaths. The intervention effect size for depression/anxiety incidence was based on a meta-analysis of studies examining the efficacy of universal and indicated SEL programs to reduce depression and/or anxiety symptoms among adolescents. The meta-analysis comprised 29 universal and 31 indicated studies gathered from a previous systematic review (Skeen *et al.*, 2019). The universal SEL program produced a standardized mean difference (SMD) of −0.10 (95% CI: −0.17 to −0.04) in reducing depression/anxiety symptoms at 1-year follow-up, while the indicated SEL program produced an SMD of −0.19 (95% CI: −0.33 to −0.05). Effect sizes attenuated completely after 1-year follow-up, a finding consistent with other studies (Hetrick *et al.*, 2016; Stockings *et al.*, 2016). The Cochrane conversion method (see Appendix S4) was used to transform SMD effect sizes into corresponding relative risk (RR) effect sizes (Lee *et al.*, 2018). The universal SEL program led to an RR of 0.84 (95% CI: 0.75 to 0.94), while the indicated SEL program produced an RR of 0.73 (95% CI: 0.57 to 0.93).

The intervention effect size for suicide mortality was based on a meta-analysis of three studies identified by a WHO review (WHO, 2015) that contributed to an update of mhGAP evidence-based guidelines (WHO, 2022). School-based SEL programs produced an RR of 0.65 (95% CI: 0.51 to 0.83) in reducing suicide attempts after 1-year follow-up. Appendix S5 presents a mathematical proof demonstrating how post-intervention reductions in suicide mortality can be estimated using an effect size involving suicide attempts. To summarize, if the case fatality proportion of suicide attempts is assumed to be constant before and after an intervention, then it is possible to directly apply the effect size for suicide attempts to suicide mortality. Following feedback from the international expert panel, a lower intervention effect size was adopted by using the upper confidence interval bound as the point estimate – a conservative modelling choice. Both universal and indicated school-based SEL programs were subsequently estimated to produce an RR of 0.83 (95% CI: 0.70 to 0.99) in reducing suicide mortality after 1-year follow-up. The derivation of intervention effect sizes is further described in Appendix S3.

### Health impact modelling

**Figure 2.**
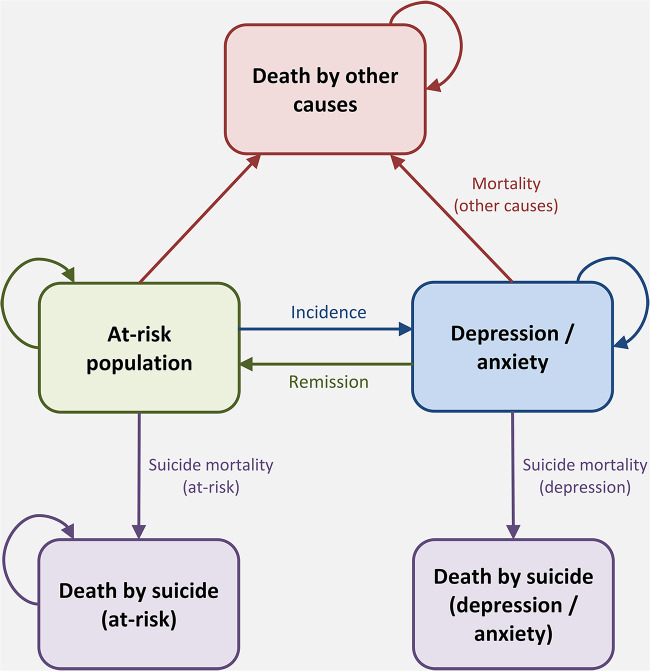
State transition diagram representing the transitions between different health states with the model.

The state transition diagram in Fig. 2 outlines how each age–sex cohort in the model transitions between health states over time. Country-specific data on suicide mortality and the epidemiology of depression and anxiety (i.e., prevalence, incidence and remission) were obtained by age and sex from Global Burden of Disease Study 2017 (GBD 2017) (IHME, 2022). The dependent comorbidity method (Mathers *et al.*, 2006) was used to combine separate estimates on the epidemiology of ‘depression’ and ‘anxiety’ into a single estimate on the epidemiology of ‘depression and/or anxiety’ (see Appendix S6). The prevalence of depression/anxiety determined the number of people in the ‘depression/anxiety’ health state at baseline, while the ‘at-risk’ health state included those without a diagnosis of depression/anxiety. The Markov model estimated the annual numbers of people who had an incident case of depression/anxiety, remitted, died from suicide, or died from other causes over time.

Post-intervention outcomes of interest included a reduction in the total number of incident depression/anxiety cases and suicide deaths. Intervention effect sizes were applied to the incidence of depression/anxiety and suicide mortality linked to the ‘at-risk’ and ‘depression/anxiety’ health states. The ‘at-risk’ health state comprised all school students aged 12–17 years in the universal program and all students aged 12–17 years with subthreshold depression/anxiety in the indicated program. Intervention effect sizes were applied for a total post-intervention duration of 1 year. Age–sex cohorts that remained in school (up to age 17 years) would receive the full intervention effect for another year, following repeated exposure to the universal/indicated SEL program. The impact of variations in participant engagement and/or adherence were not explicitly accounted for in the model. Nevertheless, the influence of engagement/adherence was implicitly incorporated as part of the intervention effect sizes, which reflect the levels of engagement/adherence that are observed across the different studies included in each respective meta-analysis.

Intervention health impacts were summarized using the HLYGs measure, which is equivalent to averted disability-adjusted life years and is made up of years of life lost (YLL) and years lived with disability (YLDs). YLLs were estimated for each age–sex cohort by taking the number of deaths in a particular year and multiplying this by potential YLL. Potential YLLs were calculated as the lowest value of either the difference between the current age of the cohort and the average life expectancy in the country or the difference between the current age of the cohort and the remaining time before the end of the 100-year model time horizon. YLDs were estimated for each age–sex cohort by calculating the total number of depression/anxiety cases occurring in a year and multiplying this by the disability weight for depression/anxiety – which was estimated by combining the GBD 2017 disability weights for depression and anxiety (IHME, 2022), while accounting for dependent comorbidity (Mathers *et al.*, 2006). Further details on health impact modelling are provided in Appendix S3.

### Costing analysis

The costing framework and methods developed by WHO-CHOICE were used to estimate the country-specific costs of scaling up universal/indicated school-based SEL programs (Bertram *et al.*, 2021a). Country-specific intervention costs were estimated using previous costing templates developed by WHO to evaluate noncommunicable disease (NCD) prevention and control policies and interventions (WHO, 2011, 2017). These NCD costing templates were modified to account for program-related costs involved in implementing universal/indicated SEL programs (e.g., program management, advocacy, training, program delivery, supplies and equipment). Templates also accounted for four stages of policy development: the planning stage (year 1), policy development (year 2), partial implementation (years 3–5) and full implementation (year 6 onwards). Universal and indicated SEL programs were both delivered by program facilitators via group sessions occurring in the classroom. Face-to-face intervention delivery was deemed the most appropriate modality for scaling up these programs in resource-constrained settings. Data from studies included in the prior meta-analysis of intervention effect sizes were used to estimate resource use associated with program delivery (e.g., time required to train program facilitators and total contact time with students). The indicated SEL program further encompassed time spent by facilitators to screen students for subthreshold depression/anxiety using locally validated mental health questionnaires. See Appendix S7 for further details.

### Uncertainty analysis and sensitivity analysis

Uncertainty analyses were conducted to quantify the impact of input parameter uncertainty on the final cost-effectiveness results. Ersatz software (version 1.31, Brisbane, Australia; available at: http://www.epigear.com/) was used to perform Monte Carlo simulation with 1,000 iterations and produce results with 95% uncertainty intervals (95% UI). Univariate deterministic sensitivity analyses were conducted to test how cost-effectiveness ratios in the base case analysis would change following a 10% increase/decrease in the mean value of each input parameter (up to 425 in total) when varied one-by-one. Multivariate probabilistic sensitivity analyses were done to analyse the strength of association (measured using Spearman’s rank correlation coefficient, *r*_s_) between each input parameter on the resulting cost-effectiveness ratio, while simultaneously accounting for interactions between other input parameters. Univariate and multivariate sensitivity analyses make known which input parameters have the greatest impact on the cost-effectiveness ratio. They highlight which input parameters need to be estimated with greater precision to reduce uncertainty around the final result. Additional sensitivity analyses were performed to examine how cost-effectiveness ratios would change after (1) excluding the intervention effect size for suicide mortality and (2) applying different annual discount rates to health impacts (i.e., 3% and 6%, instead of 0%) and intervention costs (i.e., 0% and 6%, instead of 3%). A threshold analysis was also done to examine the effect of incrementally reducing both of the intervention effect sizes applied to depression/anxiety cases and suicide deaths from 0% to 100% (with a reduction of 100% equating to an RR of 1.00).

## Results

The model estimated that the total intervention cost of scaling up the universal school-based SEL program would be I$0.096 per capita among LLMICs and I$0.162 per capita among UMHICs. Across the 20 countries, the universal SEL program could potentially avert an additional 742 depression/anxiety cases and 0.581 suicides per 1 million population. By comparison, the total intervention cost for the indicated school-based SEL program would be I$0.060 per capita among LLMICs and I$0.084 per capita among UMHICs. Across the 20 countries, the indicated SEL program could avert an additional 45 depression/anxiety cases and 0.017 suicides per 1 million population. The total cost of scaling up universal and indicated SEL programs was comparable to other low-cost, clinical interventions for the treatment of common mental disorders. For example, a previous WHO-CHOICE analysis estimated that the total cost of scaling up basic psychosocial support for depression and anxiety ranged between I$0.115 and I$0.159 per capita (WHO, 2013).

A summary of population-standardized results for the base case analysis is shown in Table 2. When analysing the universal SEL program, the cost-effectiveness ratio was estimated to be I$958 per HLYG among LLMICs and I$2,006 per HLYG among UMHICs. When analysing the indicated SEL program, the cost-effectiveness ratio was much larger at I$11,123 per HLYG among LLMICs and I$18,473 per HLYG among UMHICs. Absolute results that were not population-standardized are shown in Appendix S8. These results are presented alongside detailed breakdowns of YLDs, YLLs and the total number of depression/anxiety cases and suicide deaths averted (expressed in absolute and population-standardized units).

**Table 2.** Population-standardized results for the base case analysis

Intervention type and country income group	Population in 2017 (millions)	CE ratio (I$ per HLYG) (95% UI)	Intervention costs (2017 I$)^a^ (95% UI)	Healthy life years gained^a^ (95% UI)
Universal SEL program				
LLMICs (*n* = 10)	2,520	$958 (724–1,337)	$95,548 (88,127–102,939)	99.70 (72.09–128.88)
UMHICs (*n* = 10)	2,516	$2,006 (1,551–2,782)	$162,213 (148,977–175,088)	80.86 (58.18–102.89)
Indicated SEL program				
LLMICs (*n* = 10)	2,520	$11,123 (6,003–22,290)	$59,600 (55,082–64,179)	5.36 (2.65–9.57)
UMHICs (*n* = 10)	2,516	$18,473 (10,546–37,703)	$84,284 (77,792–90,298)	4.56 (2.16–8.01)

Abbreviations: 95% UI, 95% uncertainty interval; CE, cost-effectiveness; HLYG, healthy life year gained; I$, international dollars; LLMICs, low- to lower middle-income countries; UMHICs, upper middle- to high-income countries.

a Per 1 million population per year.

The majority of aggregate health impacts (measured in terms of HLYGs) were attributable to YLDs averted due to the prevention of depression/anxiety cases, rather than YLLs averted due to the prevention of suicides. For example, across the 20 countries, YLDs averted represented 83.9% of total HLYGs for the universal SEL program and 91.3% of total HLYGs for the indicated SEL program. The small proportion of health gains attributable to averted suicides is explained by the comparatively smaller (absolute) number of suicide deaths that occur among adolescents, when compared to incident cases of depression/anxiety. The indicated SEL program was much less cost-effective when compared to the universal SEL program. This was due to the large costs involved with screening students, alongside health impacts accruing to a narrow subset of students.

The results of the univariate and multivariate sensitivity analyses are presented, respectively, in Appendix S9 and S10. The cost-effectiveness ratio for the universal SEL program was highly sensitive to changes in the intervention effect size applied to depression/anxiety cases, the intervention effect size applied to suicide deaths, and the disability weight for depression/anxiety. In addition to these same parameters, the cost-effectiveness ratio for the indicated SEL program was highly sensitive to changes in the increased risk of developing depression/anxiety among individuals with subthreshold depression/anxiety and the prevalence of subthreshold depression/anxiety. Additional sensitivity analyses (see Table 3) found that cost-effectiveness ratios for the universal and indicated SEL programs marginally increased (i.e., were slightly less cost-effective) when excluding the intervention effect size for suicide mortality and greatly increased (i.e., were much less cost-effective) when using a 6% discount rate for health impacts. The results of the threshold analysis are presented in Appendix S11. Cost-effectiveness ratios for the universal SEL program remained below I$5,000 per HLYG after intervention effect sizes decreased by 80% among LLMICs and 59% among UMHICs. By contrast, cost-effectiveness ratios for the indicated SEL program remained below I$50,000 per HLYG following a 77% and 63% reduction in intervention effect sizes among LLMICs and UMHICs, respectively.

**Table 3.** Results for the sensitivity analyses (i) excluding the intervention effect size applied to suicide mortality and (ii) applying different discount rates to health impacts and intervention costs

Intervention type and country income group	Base case^a^ CE ratio (I$ per HLYG) (95% UI)	Exclude effect size for suicide mortality CE ratio (I$ per HLYG) (95% UI)	Health Impacts 3% CE ratio (I$ per HLYG) (95% UI)	Health Impacts 6% CE ratio (I$ per HLYG) (95% UI)	Costs 0% CE ratio (I$ per HLYG) (95% UI)	Costs 6% CE ratio (I$ per HLYG) (95% UI)
Universal SEL program						
LLMICs (*n* = 10)	$958 (724–1,337)	$1,177 (887–1,636)	$2,598 (1,959–3,601)	$4,618 (3,479–6,394)	$3,200 (2,412–4,434)	$479 (361–663)
UMHICs (*n* = 10)	$2,006 (1,551–2,782)	$2,314 (1,787–3,185)	$5,507 (4,241–7,763)	$9,826 (7,556–13,614)	$6,678 (5,146–9,227)	$1,004 (773–1,397)
Indicated SEL program						
LLMICs (*n* = 10)	$11,123 (6,003–22,290)	$12,363 (6,959–24,525)	$31,313 (17,360–61,129)	$56,691 (31,065–112,622)	$36,684 (20,404–72,918)	$5,624 (3,119–11,257)
UMHICs (*n* = 10)	$18,473 (10,546–37,703)	$19,812 (11,335–41,337)	$51,574 (29,383–107,206)	$92,309 (53,029–192,840)	$61,243 (34,808–129,456)	$9,265 (5,307–19,293)

Abbreviations: 95% UI, 95% uncertainty interval; CE, cost-effectiveness; HLYG, healthy life year gained; I$, international dollars; LLMICs, low- to lower middle-income countries; UMHICs, upper middle- to high-income countries.

a The base case scenario applied a discount rate of 0% to health impacts and 3% to intervention costs.

## Discussion

### Summary of findings

This study aimed to identify the expected population-level costs and health impacts of implementing universal and indicated school-based SEL programs at scale in secondary schools. The results suggest that implementation costs for both are similarly modest, ranging from an annual per capita investment of I$0.10 in LLMICs to I$0.16 in UMHICs for the universal SEL program and I$0.06 in LLMICs to I$0.09 in UMHICs for the indicated SEL program. However, the universal SEL program generated 20 times as much population health gain (e.g., 100 HLYGs per 1 million population were generated by universal SEL in LLMICs compared to 5 for indicated SEL). Considering costs and effects together, the cost per HLYG for the universal SEL program was just below I$1,000 in LLMICS (I$958) and one order of magnitude higher in UMHICs (I$2,006), whereas the average cost-effectiveness ratio for the indicated SEL program was substantially higher than that of the universal SEL program (I$11,123 and I$18,473 in LLMICs and UMHICs, respectively).

These cost-effectiveness results can be contextualized by referencing other health interventions for which equivalent data are available. For instance, the cost per HLYG associated with increased excise taxes on tobacco or alcoholic beverages is in the range of I$100 in LLMICs, episodic treatment of depression or ischaemic heart disease falls in the range I$100–1,000, and management of diabetes and its complications exceed I$1,000 (Bertram *et al.*, 2021b). This study solely quantified health impacts produced by school-based SEL programs in relation to the prevention of depression, anxiety and self-harm among 12- to 17-year-old adolescents. It did not quantify other known impacts of school-based SEL programs, such as students’ attitudes to self, others and their school; school commitment; academic achievement; impacts on health risk behaviours (e.g., alcohol/substance use) and impacts on parents. Furthermore, the school-based SEL programs were modelled as stand-alone interventions; when in practice, there may be opportunities to integrate them within other existing school-based programs focused on students’ health, well-being and social skills (WHO and UNESCO, 2021a).

The results of this study add to the findings of previous economic evaluations of school-based SEL programs to improve adolescent mental health and well-being (Le *et al.*, 2021). Several model-based economic evaluations have found that universal/indicated school-based SEL programs are likely cost-effective in preventing depression and anxiety (Lee *et al.*, 2017, 2021a, 2021b; Mihalopoulos *et al.*, 2012; Simon *et al.*, 2013; Ssegonja *et al.*, 2020). Conversely, trial-based economic evaluations demonstrate mixed evidence of cost-effectiveness – with two studies finding no evidence of cost-effectiveness (Anderson *et al.*, 2014; Stallard *et al.*, 2013) and one finding that an indicated SEL program was good value for money (Lynch *et al.*, 2019). Evidence on the cost-effectiveness of school-based SEL programs to prevent suicide outcomes was similarly mixed – with one model-based study observing that a universal SEL program was cost-saving under a societal perspective (but not a health sector perspective) (Kinchin *et al.*, 2020) and another trial-based study finding no evidence of cost-effectiveness (Ahern *et al.*, 2018). A recent return-on-investment analysis found that universal and indicated SEL programs can produce economic benefits that far exceed the costs of implementation – particularly when accounting for future improvements in employment and earnings potential that accrue to individuals experiencing improved mental health outcomes during adolescence (Stelmach *et al.*, 2022).

### Application and relevance of study findings

These findings assume new relevance when considering the inclusion of school-based SEL as an integral and key element of Helping Adolescents Thrive (HAT) – an evidence-based programmatic guidance package developed by WHO and UNICEF to enhance mental health promotion and protection and to reduce risk behaviours among adolescents. Early uptake of HAT at a country level has indicated strong interest in establishing or enhancing school-based mental health programs and has informed the development of the HAT Teacher’s Guide and related comic book to facilitate integration of SEL in the school curricula of young adolescents (UNICEF and WHO, 2021). Delivering SEL in schools should be considered a single element within a multipronged strategy for implementing school-based mental health promotive policies that are closely linked to other health services and referral mechanisms currently delivered in schools (NICE, 2022; WHO and UNESCO, 2021a, 2021b). Despite producing fewer population-level health benefits, the implementation of indicated SEL programs may be justified as a means of reducing population inequalities that affect high-risk populations who would benefit from a more tailored intervention approach.

The cost-effectiveness findings in this study are highly sensitive to fluctuations around the intervention effect sizes that were input into the model. It follows that any prospective rollout should proactively address implementation challenges that can reduce the fidelity and/or quality of SEL program delivery (Durlak, 2016; Kaspar and Massey, 2023). For example, inadequate participant engagement and/or adherence can adversely impact on the real-world effectiveness of SEL programs. The WHO’s HAT Toolkit provides detailed operational guidance on how to implement SEL programs (WHO and UNICEF, 2021). Nine implementation factors have been identified to improve the likelihood that scaled up programs achieve their intended impacts. These include involving the adolescent target group and key stakeholders (e.g., caregivers and teachers) in the program development and implementation, ensuring the integration of SEL programs within the broader education curricula and adapting program materials to the local cultural context while ensuring intervention fidelity. Moreover, ongoing monitoring and evaluation is vital to bringing about effective program implementation.

While this study was initiated before the onset of the pandemic, its findings offer valuable information to national policymakers confronted with the deep and far-reaching mental health impacts of COVID-19 on young people, particularly the quantification of the expected level of prevented cases of depression, anxiety and suicide if school-based SEL programs are fully implemented. Additionally, the newly developed model can be, and already has been, applied at the country level to inform national mental health investment cases. In the Philippines, for example, an investment of 68 Philippine pesos (US$ 1.10) per capita in universal school-based SEL programs for 12- to 17-year-olds has the potential to avert over 450,000 cases of depression/anxiety and 377 suicides over a period of 10 years (WHO, 2021b).

### Limitations

This study has several limitations. Data have been synthesized from various sources, with estimates of intervention effect size largely obtained from high-income country settings where more research and evaluation studies have been completed. Further research on the effectiveness of SEL programs in low- and middle-income countries is required, alongside consideration of cultural adaptations to improve program acceptance and uptake. This analysis has deliberately considered the combined effects of SEL interventions on depression/anxiety and suicide, even though many programs implemented in practice only consider impacts on one of these outcomes. Post hoc analysis of results indicates that over 90% of the estimated HLYG are attributable to the reduced incidence of depression/anxiety, so this might be considered a lower bound estimate of the potential health impact of these interventions. Use of the Cochrane conversion method to impute RR effect sizes from SMD effect sizes may limit the internal validity of study findings. Moreover, the assumption that reductions in suicide attempts lead to commensurate reductions in suicide deaths should be further explored, particularly in settings where prior suicide attempts are weaker predictors of completed suicides. Improving the accurate measurement of intervention effect sizes is vital, given the sensitivity of cost-effectiveness findings to this input parameter.

## Conclusion

This study adds to current global evidence on SEL programs as a recommended strategy for child and adolescent mental health promotion and protection by quantifying their expected health impacts and costs when implemented at scale in different country income settings. It suggests that both universal and indicated SEL programs require a low level of investment (in the range of I$0.05 to I$0.20 per person) but that universal SEL programs produce significantly greater health benefits and therefore better value for money (e.g., less than I$1,000 per HLYG in LLMICs). Any prospective implementation of these programs in schools should take proactive steps to ensure the fidelity and quality of SEL program delivery and, in turn, the realization of the potential cost-effectiveness gains identified in this study.

## Data Availability

In accordance with the WHO open access policy, data collected for this study are being made available to others on the Figshare repository through a CC BY 4.0 license at the date of publication, including country-level demographic and epidemiological data, other model parameters relating to intervention costs and effectiveness and the model structure itself. Application of the CC BY 4.0 license requires interested users of the data to attribute the original source.

## Supplementary material

For supplementary material accompanying this paper visit http://doi.org/10.1017/S204579602300029X. click here to view supplementary material

## References

[ref1] Ahern S, Burke LA, McElroy B, Corcoran P, McMahon EM, Keeley H, Carli V, Wasserman C, Hoven CW, Sarchiapone M, Apter A, Balazs J, Banzer R, Bobes J, Brunner R, Cosman D, Haring C, Kaess M, Kahn JP, Kereszteny A, Postuvan V, Saiz PA, Varnik P and Wasserman D (2018) A cost-effectiveness analysis of school-based suicide prevention programmes. *European Child & Adolescent Psychiatry* 27, 1295–1304. 29442231 10.1007/s00787-018-1120-5

[ref2] Anderson R, Ukoumunne OC, Sayal K, Phillips R, Taylor JA, Spears M, Araya R, Lewis G, Millings A, Montgomery AA and Stallard P (2014) Cost-effectiveness of classroom-based cognitive behaviour therapy in reducing symptoms of depression in adolescents: A trial-based analysis. *Journal of Child Psychology and Psychiatry, and Allied Disciplines* 55, 1390–1397. 24813670 10.1111/jcpp.12248

[ref3] Avenir Health (2022) OneHealth tool. https://www.avenirhealth.org/software-onehealth.php (accessed 20 July 2022).

[ref4] Bertha EA and Balazs J (2013) Subthreshold depression in adolescence: A systematic review. *European Child & Adolescent Psychiatry* 22, 589–603. 23579389 10.1007/s00787-013-0411-0

[ref5] Bertram MY and Edejer TTT (2021) Introduction to the special issue on “The World Health Organization Choosing Interventions That Are Cost-Effective (WHO-CHOICE) Update”. *International Journal of Health Policy and Management* 10, 670–672. 34634892 10.34172/ijhpm.2021.105 PMC9278374

[ref6] Bertram MY, Lauer JA, Stenberg K and Edejer TTT (2021a) Methods for the economic evaluation of health care interventions for priority setting in the health system: An update from WHO CHOICE. *International Journal of Health Policy and Management* 10, 673–677. 33619929 10.34172/ijhpm.2020.244 PMC9278384

[ref7] Bertram MY, Lauer JA, Stenberg K, Ralaidovy AH and Edejer TT (2021b) Progressive realisation of universal health coverage in low- and middle-income countries: Beyond the “Best Buys”. *International Journal of Health Policy and Management* 10, 697–705. 33619938 10.34172/ijhpm.2020.245 PMC9278372

[ref8] Bertram MY, Stenberg K, Brindley C, Li J, Serje J, Watts R and Edejer TT (2017) Disease control programme support costs: An update of WHO-CHOICE methodology, price databases and quantity assumptions. *Cost Effectiveness and Resource Allocation* 15, 21. 10.1186/s12962-017-0083-6 PMC5658944 29089861

[ref9] Carrellas NW, Biederman J and Uchida M (2017) How prevalent and morbid are subthreshold manifestations of major depression in adolescents? A literature review. *Journal of Affective Disorders* 210, 166–173. 28049101 10.1016/j.jad.2016.12.037

[ref10] de Girolamo G, Dagani J, Purcell R, Cocchi A and McGorry PD (2012) Age of onset of mental disorders and use of mental health services: Needs, opportunities and obstacles. *Epidemiology and Psychiatric Sciences* 21, 47–57. 22670412 10.1017/s2045796011000746

[ref11] Durlak JA (2016) Programme implementation in social and emotional learning: Basic issues and research findings. *Cambridge Journal of Education* 46, 333–345.

[ref12] Esmaeelzadeh S, Moraros J, Thorpe L and Bird Y (2018) Examining the association and directionality between mental health disorders and substance use among adolescents and Young Adults in the U.S. and Canada – A systematic review and meta-analysis. *Journal of Clinical Medicine* 7, 543. 10.3390/jcm7120543 PMC6306768 30551577

[ref13] Fazel M, Hoagwood K, Stephan S and Ford T (2014a) Mental health interventions in schools 1: Mental health interventions in schools in high-income countries. *The Lancet Psychiatry* 1, 377–387. 26114092 10.1016/S2215-0366(14)70312-8 PMC4477835

[ref14] Fazel M, Patel V, Thomas S and Tol W (2014b) Mental health interventions in schools in low-income and middle-income countries. *The Lancet Psychiatry* 1, 388–398. 26361001 10.1016/S2215-0366(14)70357-8

[ref15] Haller H, Cramer H, Lauche R, Gass F and Dobos GJ (2014) The prevalence and burden of subthreshold generalized anxiety disorder: A systematic review. *BMC Psychiatry* 14, 128. 10.1186/1471-244X-14-128 PMC4048364 24886240

[ref16] Hetrick SE, Cox GR, Witt KG, Bir JJ and Merry SN (2016) Cognitive behavioural therapy (CBT), third-wave CBT and interpersonal therapy (IPT) based interventions for preventing depression in children and adolescents. *Cochrane Database of Systematic Reviews* 2016(8), CD003380. 10.1002/14651858.CD003380.pub4 PMC8407360 27501438

[ref17] Husereau D, Drummond M, Petrou S, Carswell C, Moher D, Greenberg D, Augustovski F, Briggs AH, Mauskopf J, Loder E and Force CT (2013) Consolidated Health Economic Evaluation Reporting Standards (CHEERS) statement. *BMJ* 346, f1049. 10.1136/bmj.f1049 23529982

[ref18] IHME (2022) Global burden of disease study 2017 (GBD 2017) data resources. https://ghdx.healthdata.org/gbd-2017 (accessed 10 February 2022).

[ref19] Kaspar KL and Massey SL (2023) Implementing social-emotional learning in the elementary classroom. *Early Childhood Education Journal* 51, 641–650. 35250255 10.1007/s10643-022-01324-3 PMC8881978

[ref20] Kinchin I, Russell AMT, Petrie D, Mifsud A, Manning L and Doran CM (2020) Program evaluation and decision analytic modelling of universal suicide prevention training (safeTALK) in secondary schools. *Applied Health Economics and Health Policy* 18, 311–324. 31410773 10.1007/s40258-019-00505-3

[ref21] Lee YY, Barendregt JJ, Stockings EA, Ferrari AJ, Whiteford HA, Patton GA and Mihalopoulos C (2017) The population cost-effectiveness of delivering universal and indicated school-based interventions to prevent the onset of major depression among youth in Australia. *Epidemiology and Psychiatric Sciences* 26, 545–564. 27509769 10.1017/S2045796016000469 PMC6998892

[ref22] Lee YY, Le LK, Lal A, Engel L and Mihalopoulos C (2021a) The cost-effectiveness of delivering an e-health intervention, MoodGYM, to prevent anxiety disorders among Australian adolescents: A model-based economic evaluation. *Mental Health & Prevention* 24, 200210.

[ref23] Lee YY, Le LK, Lal A, Engel L and Mihalopoulos C (2021b) The cost-effectiveness of delivering universal psychological interventions in schools to prevent depression among Australian adolescents: A model-based economic evaluation. *Mental Health & Prevention* 24, 200213.

[ref24] Lee YY, Le LK, Stockings EA, Hay P, Whiteford HA, Barendregt JJ and Mihalopoulos C (2018) Estimation of a relative risk effect size when using continuous outcomes data: An application of methods in the prevention of major depression and eating disorders. *Medical Decision Making* 38, 866–880. 30156470 10.1177/0272989X18793394

[ref25] Le LK, Esturas AC, Mihalopoulos C, Chiotelis O, Bucholc J, Chatterton ML and Engel L (2021) Cost-effectiveness evidence of mental health prevention and promotion interventions: A systematic review of economic evaluations. *PLoS Medicine* 18, e1003606. 10.1371/journal.pmed.1003606 PMC8148329 33974641

[ref26] Lynch FL, Dickerson JF, Clarke GN, Beardslee WR, Weersing VR, Gladstone TRG, Porta G, Brent DA, Mark TL, DeBar LL, Hollon SD and Garber J (2019) Cost-effectiveness of preventing depression among at-risk youths: Postintervention and 2-year follow-up. *Psychiatric Services* 70, 279–286. 30929618 10.1176/appi.ps.201800144 PMC6897501

[ref27] Mathers CD, Iburg KM and Begg S (2006) Adjusting for dependent comorbidity in the calculation of healthy life expectancy. *Population Health Metrics* 4, 4. 10.1186/1478-7954-4-4 PMC1484491 16620383

[ref28] Mihalopoulos C, Vos T, Pirkis J and Carter R (2012) The population cost-effectiveness of interventions designed to prevent childhood depression. *Pediatrics* 129, e723–e730. 22312000 10.1542/peds.2011-1823

[ref29] Mrazek PJ and Haggerty RJ (1994) *Reducing Risks for Mental Disorders: Frontiers for Preventive Intervention Research*. Washington DC, USA: National Academies Press. 25144015

[ref30] Munasinghe S, Sperandei S, Freebairn L, Conroy E, Jani H, Marjanovic S and Page A (2020) The impact of physical distancing policies during the COVID-19 pandemic on health and well-being among Australian Adolescents. *Journal of Adolescent Health* 67, 653–661. 10.1016/j.jadohealth.2020.08.008 PMC7577185 33099413

[ref31] NICE (2022) Social, emotional and mental wellbeing in primary and secondary education – NICE guideline [NG223]. https://www.nice.org.uk/guidance/ng223 (accessed 20 July 2022).

[ref32] Patton GC, Sawyer SM, Santelli JS, Ross DA, Afifi R, Allen NB, Arora M, Azzopardi P, Baldwin W, Bonell C, Kakuma R, Kennedy E, Mahon J, McGovern T, Mokdad AH, Patel V, Petroni S, Reavley N, Taiwo K, Waldfogel J, Wickremarathne D, Barroso C, Bhutta Z, Fatusi AO, Mattoo A, Diers J, Fang J, Ferguson J, Ssewamala F and Viner RM (2016) Our future: A Lancet commission on adolescent health and wellbeing. *Lancet* 387, 2423–2478. 27174304 10.1016/S0140-6736(16)00579-1 PMC5832967

[ref33] Simon E, Dirksen CD and Bogels SM (2013) An explorative cost-effectiveness analysis of school-based screening for child anxiety using a decision analytic model. *European Child & Adolescent Psychiatry* 22, 619–630. 23539355 10.1007/s00787-013-0404-z

[ref34] Skeen S, Laurenzi CA, Gordon SL, du Toit S, Tomlinson M, Dua T, Fleischmann A, Kohl K, Ross D, Servili C, Brand AS, Dowdall N, Lund C, van der Westhuizen C, Carvajal-Aguirre L, Eriksson de Carvalho C and Melendez-Torres GJ (2019) Adolescent mental health program components and behavior risk reduction: A meta-analysis. *Pediatrics* 144, e20183488. 10.1542/peds.2018-3488 31262779

[ref35] Solmi M, Radua J, Olivola M, Croce E, Soardo L, Salazar de Pablo G, Il Shin J, Kirkbride JB, Jones P, Kim JH, Kim JY, Carvalho AF, Seeman MV, Correll CU and Fusar-Poli P (2022) Age at onset of mental disorders worldwide: Large-scale meta-analysis of 192 epidemiological studies. *Molecular Psychiatry* 27, 281–295. 34079068 10.1038/s41380-021-01161-7 PMC8960395

[ref36] Ssegonja R, Sampaio F, Alaie I, Philipson A, Hagberg L, Murray K, Sarkadi A, Langenskiold S, Jonsson U and Feldman I (2020) Cost-effectiveness of an indicated preventive intervention for depression in adolescents: A model to support decision making. *Journal of Affective Disorders* 277, 789–799. 33065819 10.1016/j.jad.2020.08.076

[ref37] Stallard P, Phillips R, Montgomery AA, Spears M, Anderson R, Taylor J, Araya R, Lewis G, Ukoumunne OC, Millings A, Georgiou L, Cook E and Sayal K (2013) A cluster randomised controlled trial to determine the clinical effectiveness and cost-effectiveness of classroom-based cognitive-behavioural therapy (CBT) in reducing symptoms of depression in high-risk adolescents. *Health Technology Assessment* 17, vii–xvii, 1–109. 10.3310/hta17470 PMC4781207 24172024

[ref38] Stelmach R, Kocher EL, Kataria I, Jackson-Morris AM, Saxena S and Nugent R (2022) The global return on investment from preventing and treating adolescent mental disorders and suicide: A modelling study. *BMJ Global Health* 7, e007759. 10.1136/bmjgh-2021-007759 PMC9240828 35705224

[ref39] Stockings EA, Degenhardt L, Dobbins T, Lee YY, Erskine HE, Whiteford HA and Patton G (2016) Preventing depression and anxiety in young people: A review of the joint efficacy of universal, selective and indicated prevention. *Psychological Medicine* 46, 11–26. 26315536 10.1017/S0033291715001725

[ref40] UIS (2022) Welcome to UIS.Stat. http://data.uis.unesco.org/ (accessed 25 August 2019).

[ref41] UN DESA (2017) World population prospects: The 2017 revision. https://www.un.org/development/desa/publications/world-population-prospects-the-2017-revision.html (accessed 3 August 2019).

[ref42] UNICEF and WHO (2021) Teacher’s guide to the magnificent Mei and friends series (WHO-UNICEF Helping Adolescents Thrive [HAT] initiative). https://apps.who.int/iris/handle/10665/341349 (accessed 20 July 2022).

[ref43] Wasserman D, Hoven CW, Wasserman C, Wall M, Eisenberg R, Hadlaczky G, Kelleher I, Sarchiapone M, Apter A, Balazs J, Bobes J, Brunner R, Corcoran P, Cosman D, Guillemin F, Haring C, Iosue M, Kaess M, Kahn JP, Keeley H, Musa GJ, Nemes B, Postuvan V, Saiz P, Reiter-Theil S, Varnik A, Varnik P and Carli V (2015) School-based suicide prevention programmes: The SEYLE cluster-randomised, controlled trial. *The Lancet* 385, 1536–1544. 10.1016/S0140-6736(14)61213-7 25579833

[ref44] WHO (2011) Scaling up action against noncommunicable diseases: How much will it cost? https://apps.who.int/iris/handle/10665/44706 (accessed 20 July 2022).

[ref45] WHO (2013) Comprehensive mental health action plan 2013–2030. https://apps.who.int/iris/handle/10665/345301 (accessed 20 July 2022).

[ref46] WHO (2015) School-based interventions for reducing deaths from suicide and suicide attempts among young people. https://cdn.who.int/media/docs/default-source/mental-health/mhgap/self-harm-and-suicide/school-based-interventions-for-reducing-deaths-from-suicide-and-suicide-attempts-among-young-people.pdf (accessed 20 July 2022).

[ref47] WHO (2017) Tackling NCDs: “Best buys” and other recommended interventions for the prevention and control of noncommunicable diseases. https://apps.who.int/iris/handle/10665/259232 (accessed 20 July 2022).

[ref48] WHO (2020) Guidelines on mental health promotive and preventive interventions for adolescents: Helping adolescents thrive. https://apps.who.int/iris/handle/10665/336864 (accessed 20 July 2022). 33301276

[ref49] WHO (2021a) Live life: An implementation guide for suicide prevention in countries. https://apps.who.int/iris/handle/10665/341726 (accessed 20 July 2022).

[ref50] WHO (2021b) Prevention and management of mental health conditions in the Philippines: The case for investment, Manila, Philippines: WHO Regional Office for the Western Pacific.

[ref51] WHO (2021c) Suicide worldwide in 2019: Global health estimates. Available at: https://apps.who.int/iris/handle/10665/341728 (accessed 20 July 2022).

[ref52] WHO (2021d) WHO menu of cost-effective interventions for mental health. https://apps.who.int/iris/handle/10665/343074 (accessed 20 July 2022).

[ref53] WHO (2022) mhGAP evidence resource centre. https://www.who.int/teams/mental-health-and-substance-use/treatment-care/mental-health-gap-action-programme/evidence-centre (accessed 20 July 2022).

[ref54] WHO and UNESCO (2021a) Making every school a health-promoting school: Implementation guidance. https://apps.who.int/iris/handle/10665/341908 (accessed 20 July 2022).

[ref55] WHO and UNESCO (2021b) WHO guideline on school health services. https://apps.who.int/iris/handle/10665/341910 (accessed 20 July 2022).

[ref56] WHO and UNICEF (2021) Helping adolescents thrive toolkit: Strategies to promote and protect adolescent mental health and reduce self-harm and other risk behaviours. https://apps.who.int/iris/handle/10665/341327 (accessed 20 July 2022).

