# Supplementary material for: School-based socio-emotional learning programs to prevent depression, anxiety and suicide among adolescents: a global cost-effectiveness analysis
Source: Epidemiol Psychiatr Sci. 2023 Jul 12;32:e46. doi: 10.1017/S204579602300029X (PMC10477081; doi:10.1017/S204579602300029X)
Supplement: Supplementary file 1 [file S204579602300029Xsup001.pdf]

Supplementary Appendices

Appendix S1 – Background and rationale for the analytic approach 2
Overview 2
Research context 2
WHO-CHOICE methods 2
Country income groups 3
International expert panel 3
Appendix S2 – Consolidated Health Economic Evaluation Reporting Standards (CHEERS) statement 4
Appendix S3 – Overview of model parameters 7
Appendix S4 – Description of the Cochrane conversion method 39
Appendix S5 – Mathematical equations underlying the modelling of health impacts 40
Introduction 40
Background equations for each health state 41
Equations for suicide rates and suicide probabilities 42
Expressing suicide rates in terms of the population attributable fraction 42
Expressing the suicide probabilities as a function of the suicide rate 43
Equation for the mortality probability due to other causes 44
Expressing the other cause mortality probability in terms of the all-cause mortality and suicide rates 44
Equations for the incidence probability of suicide attempts 44
Equations for the case fatality probability following a suicide attempt 45
Expressing the incidence of suicide attempts in terms of the suicide rate and case fatality of attempts 45
Applying the intervention effect size to the overall suicide rate in the population 46
Applying the intervention effect size to the suicide probability among people with depression/anxiety 46
Applying the intervention effect size to suicide probability among people at risk of depression/anxiety 47
Appendix S6 – Methods used to account for dependent comorbidity between depression and anxiety 48
Appendix S7 – Additional details on the costing analysis 50
Overview 50
Data required to cost program delivery 50
Appendix S8 – Additional results for the base case analysis 54
Appendix S9 – Univariate deterministic sensitivity analysis results 57
Appendix S10 – Multivariate probabilistic sensitivity analysis results 59
Appendix S11 – Threshold analysis results 61
Reference list 65

Appendix S1 – Background and rationale for the analytic approach

Overview

The following section outlines the research context informing the economic evaluation of universal and indicated school-based socio-emotional learning (SEL) programs to prevent depression/anxiety and suicide among adolescents aged 12-17 years. It provides a brief synopsis of WHO-CHOICE methods, alongside the background and rationale for various analytic choices implemented in the economic evaluation. These have all been discussed in further detail elsewhere.[1-3]

Research context

The current study was part of an overarching body of work carried out by the WHO Secretariat to develop a menu of policy options and cost-effective interventions for mental health.[3] Provided at the request of WHO Member States, the purpose of this menu is to assist Member States in implementing, as appropriate for national context, actions to achieve voluntary global targets for mental health through the objectives of the Mental Health Action Plan 2013-2020.[4] The list of interventions within this menu is not exhaustive and is intended to provide information and guidance on costs, effectiveness and cost-effectiveness of population-based and individual interventions based on current evidence; and to act as the basis for future work to develop and expand the evidence base. The menu has been developed in line with Appendix 3 of the WHO's Global Action Plan for the Prevention and Control of Noncommunicable Diseases (NCDs) 2013-2020;[5] which uses WHO-CHOICE methodology to prepare and update, as appropriate, estimates on the cost-effectiveness of a range of interventions. This included a new population-based economic analysis of universal and indicated school-based SEL programs.

WHO-CHOICE methods

Value for money and efficiency are fundamental considerations guiding investment in health, and WHO-CHOICE provides a way to measure them. Cost-effectiveness analysis supports priority setting by defining areas of action where the greatest health gains can be achieved. The use of cost-effectiveness analysis within decision making processes in health is increasingly common globally. However, a series of methodological shortcomings may limit the practical application of cost-effectiveness analysis results. Two examples of this are: methodological differences between studies that limit comparability; and use of the current practice as a comparator, which implicitly assumes current resource use is efficient.

Generalized Cost-Effectiveness Analysis (GCEA) was developed to overcome such shortcomings of traditional cost-effectiveness analysis.[6] The GCEA approach enables both existing and new interventions to be evaluated simultaneously. The comparator used in GCEA is a hypothetical 'null' scenario, where the impacts of all currently implemented interventions are removed. This method uniquely allows existing and new interventions to be analysed simultaneously. Using WHO-CHOICE, the analyst is no longer constrained by what is already being done, and policymakers can revisit and revise past choices if necessary and feasible. They will have a rational basis for deciding to reallocate resources between interventions to achieve social objectives. GCEA also allows the definition of an optimal set of interventions, considering setting-specific factors such as the burden of disease, health system practice and economic conditions.

WHO-CHOICE takes the costing perspective of "the health system", by which is meant the ensemble of actions and actors whose primary intent is to improve human health.[2] WHO-CHOICE therefore includes all direct, market-valued costs, whether public or private, that are required to deliver the intervention, regardless of payer. WHO-CHOICE does not account for non-monetary patient contributions such as travel time, time off work or lost income. It also does not account for costs outside of the health system, such as the cost of social services whose aim is not primarily health oriented. So the costing perspective of WHO-CHOICE is broader than the health sector per se, and is health system focused according to accepted international definitions of the health system. Other sector costs (e.g., legislation) are included to the extent that they are a direct component of the intervention that is intended to improve human health.

In addition to the health system perspective, WHO-CHOICE:

- Uses a standardised method for cost-effectiveness analysis that can be applied to all interventions in different settings;
- Evaluates all interventions relative to the “null”, a scenario in which the absence of health care interventions is estimated;
- Uses a population-based approach for estimating health impacts, measured as healthy life years gained (HLYGs) due to an intervention over a 100-year time frame, where one healthy life year gained is equivalent to one disability-adjusted life year (DALY) averted;
- Does not apply discounting to health impacts measured (i.e., HLYGs);
- Uses an ingredients-based economic costing methodology for the calculation of costs. Costs are calculated over a 100-year time frame, discounted at 3% per year and expressed in International dollars (I$) that adjust for the differences in purchasing power between countries; and
- Expresses intervention cost-effectiveness as a ratio of international dollars (I$) per healthy life year gained.

Country income groups

Economic parameters have been assessed for two country income groups: low- and lower middle-income countries (LLMICs); and upper middle- and high-income countries (UMHICs). Recognising the need for generalisability, applicability and comprehensiveness, countries were selected so that a significant proportion of the total population and health burden would be represented. The importance of representation from countries in different regional settings was also recognised. Twenty countries were included in the analysis and are listed below (these are the same countries as for WHO-CHOICE analyses underpinning Appendix 3 to the Global Action Plan for the Prevention and Control of NCDs 2013-2020).[5] Ten countries were analysed from low and lower-middle income settings, and ten from upper-middle and high-income settings. Combined, they represent >80% of the global population and the global burden of mental health conditions. All economic analyses were first conducted at the country level. Country-specific results were then aggregated to produce corresponding results for the two country income groups.

**Low- to Lower Middle-Income countries (LLMICs)**	**Upper Middle- to High-Income Countries (UMHICs)**
Bangladesh	China
Ethiopia	Germany
Guatemala	Iran (Islamic Republic of)
India	Japan
Indonesia	Mexico
Nigeria	Russian Federation
Pakistan	South Africa
Philippines	Thailand
Ukraine	Turkey
Vietnam	United States of America

International expert panel

The WHO Secretariat convened a technical consultation in Geneva on 21 August 2019 to review the epidemiologically-based population model, selected input parameters and cost-effectiveness findings for universal and indicated school-based SEL programs to prevent depression/anxiety and suicide among adolescents. International experts were invited by the WHO Secretariat based on their ability to contribute technical advice to the modelling work and to ensure adequate global representation across the six WHO regions (see Acknowledgements of the main manuscript for the full list of experts). All conflicts of interest were declared and checked prior to the meeting. Technical advice was provided in-person during the meeting and through out-of-session email communications. This review informed the development of revised estimates that were presented in a draft WHO Discussion Paper published online on 2 September 2019.

Appendix S2 – Consolidated Health Economic Evaluation Reporting Standards (CHEERS) statement

Section/item	Item No	Recommendation	Section reported
**Title and abstract**			
Title	1	Identify the study as an economic evaluation or use more specific terms such as “cost-effectiveness analysis”, and describe the interventions compared.	See Title
Abstract	2	Provide a structured summary of objectives, perspective, setting, methods (including study design and inputs), results (including base case and uncertainty analyses), and conclusions.	See Abstract
**Introduction**			
Background and objectives	3	Provide an explicit statement of the broader context for the study.	See Introduction
		Present the study question and its relevance for health policy or practice decisions.	See Introduction
**Methods**			
Target population and subgroups	4	Describe characteristics of the base case population and subgroups analysed, including why they were chosen.	See Analytic approach subsection in the Methods
Setting and location	5	State relevant aspects of the system(s) in which the decision(s) need(s) to be made.	See Analytic approach subsection in the Methods
Study perspective	6	Describe the perspective of the study and relate this to the costs being evaluated.	See Analytic approach subsection in the Methods
Comparators	7	Describe the interventions or strategies being compared and state why they were chosen.	See Analytic approach subsection in the Methods
Time horizon	8	State the time horizon(s) over which costs and consequences are being evaluated and say why appropriate.	See Demographic projections subsection in the Methods
Discount rate	9	Report the choice of discount rate(s) used for costs and outcomes and say why appropriate.	See Analytic approach subsection in the Methods
Choice of health outcomes	10	Describe what outcomes were used as the measure(s) of benefit in the evaluation and their relevance for the type of analysis performed.	See Analytic approach subsection in Methods
Measurement of effectiveness	11a	Single study-based estimates: Describe fully the design features of the single effectiveness study and why the single study was a sufficient source of clinical effectiveness data.	Not applicable
	11b	Synthesis-based estimates: Describe fully the methods used for identification of included studies and synthesis of clinical effectiveness data.	See Intervention effect sizes subsection in the Methods and Appendix S3

Section/item	Item No	Recommendation	Section reported
Measurement and valuation of preference based outcomes	12	If applicable, describe the population and methods used to elicit preferences for outcomes.	Not applicable
Estimating resources and costs	13a	Single study-based economic evaluation: Describe approaches used to estimate resource use associated with the alternative interventions. Describe primary or secondary research methods for valuing each resource item in terms of its unit cost. Describe any adjustments made to approximate to opportunity costs.	Not applicable
	13b	Model-based economic evaluation: Describe approaches and data sources used to estimate resource use associated with model health states. Describe primary or secondary research methods for valuing each resource item in terms of its unit cost. Describe any adjustments made to approximate to opportunity costs.	See Costing analysis subsection in the Methods and Appendix S7
Currency, price date, and conversion	14	Report the dates of the estimated resource quantities and unit costs. Describe methods for adjusting estimated unit costs to the year of reported costs if necessary. Describe methods for converting costs into a common currency base and the exchange rate.	See the Analytic approach and Costing analysis subsections in the Methods
Choice of model	15	Describe and give reasons for the specific type of decision-analytical model used. Providing a figure to show model structure is strongly recommended.	See the Analytic approach, Demographic projections and Health impact modelling subsections in the Methods
Assumptions	16	Describe all structural or other assumptions underpinning the decision-analytical model.	See Methods and Appendices S1 to S7
**Results**			
Study parameters	18	Report the values, ranges, references, and, if used, probability distributions for all parameters. Report reasons or sources for distributions used to represent uncertainty where appropriate. Providing a table to show the input values is strongly recommended.	See Methods and Appendix S3. Data that were not listed are in the public domain and can be readily accessed online.
Incremental costs and outcomes	19	For each intervention, report mean values for the main categories of estimated costs and outcomes of interest, as well as mean differences between the comparator groups. If applicable, report incremental cost-effectiveness ratios.	See Results and Table 2 in the main manuscript. See also Appendix S8.
Characterising uncertainty	20a	Single study-based economic evaluation: Describe the effects of sampling uncertainty for the estimated incremental cost and incremental effectiveness parameters, together with the impact of methodological assumptions (such as discount rate, study perspective).	Not applicable

Section/item	Item No	Recommendation	Section reported
	20b	Model-based economic evaluation: Describe the effects on the results of uncertainty for all input parameters, and uncertainty related to the structure of the model and assumptions.	See Results, Table 2 and Table 3 in the main manuscript. Also see Appendices S8 to S10.
Characterising heterogeneity	21	If applicable, report differences in costs, outcomes, or cost-effectiveness that can be explained by variations between subgroups of patients with different baseline characteristics or other observed variability in effects that are not reducible by more information.	See Results
**Discussion**			
Study findings, limitations, generalisability, and current knowledge	22	Summarise key study findings and describe how they support the conclusions reached. Discuss limitations and the generalisability of the findings and how the findings fit with current knowledge.	See Discussion
Other			
Source of funding	23	Describe how the study was funded and the role of the funder in the identification, design, conduct, and reporting of the analysis. Describe other non-monetary sources of support.	See Financial Support
Conflicts of interest	24	Describe any potential for conflict of interest of study contributors in accordance with journal policy. In the absence of a journal policy, we recommend authors comply with International Committee of Medical Journal Editors recommendations.	See Conflicts of Interest

Appendix S3 – Overview of model parameters

Appendix Table 1 Summary of data used to inform model parameters

Section	Model parameter	Description
Demographic projections	Population	Data on the 2017 population were obtained by country, age and sex from the OneHealth Tool.[7]
	All-cause mortality	Data on all-cause mortality rates were obtained by country, age and sex from the OneHealth Tool.[7] Data were available by year over the 100-year model time horizon.
	New births	The rate of new births was estimated using country-specific data on the crude birth rate and the sex ratio at birth obtained from WPP 2017.[8] Data were available by year over the 100-year model time horizon.
	Net migration	Country-specific data on the net migration rate were obtained from WPP 2017.[8] Data were available by year over the 100-year model time horizon.
Intervention coverage	Secondary school attendance rate	Universal and indicated school-based SEL programs were modelled with a population coverage of 95% among adolescents aged 12-17 years. An additional adjustment was made to exclude adolescents who do not attend secondary school by multiplying population coverage with the secondary school attendance rate in each country. Data on the percentage of adolescents who attend secondary school were based on the (inverse) rate of out-of-school adolescents that were of secondary school age. Data were obtained by country and sex from the UIS Statistics (UIS.Stat) database published by UNESCO.[9]
	Prevalence of subthreshold depression/anxiety	Under the indicated SEL program, intervention coverage was restricted to students with subthreshold depression/anxiety. Based on the findings of previous reviews,[10-12] it was estimated that 5% of adolescents (range: 1% to 9%) would have subthreshold depression/anxiety.
Health impact modelling	Intervention effect sizes	Two sets of intervention effect sizes were applied in the model.<br><br>Intervention effect sizes for depression/anxiety were based on results from a systematic review of adolescent mental health programs, encompassing 158 included studies.[13] In-scope studies for meta-analyses were limited to those involving: (1) depression/anxiety; and (2) program delivery in school settings. Intervention effect size data were collected for all available trial arms and time points; and were expressed as a standardised mean difference. A total of 29 universal studies and 31 indicated studies were deemed in-scope. Meta-analyses were performed using the robust variance estimation (RVE) method and the 'robumeta' package in Stata. The RVE method accounts for dependence between observations and enables the inclusion of all relevant information while generating consistent and asymptotically accurate estimates of standard errors. A within-study intercorrelation parameter of 0.8 was assumed. The universal SEL program produced a standardised mean difference (SMD) of -0.10 (95% CI: -0.17 to -0.04) in reducing depression/anxiety symptoms at 1-year follow-up, while the indicated SEL program produced a SMD of -0.19 (95% CI: -0.33 to -0.05). Effect sizes were observed to attenuate completely after 1-year follow-up; a finding that is consistent with other similar studies.[14,15] The Cochrane conversion method, described in Appendix S4, was used to transform SMD effect sizes into corresponding relative risk (RR) effect sizes.[16] The universal SEL program consequently led to a RR of 0.84 (95% CI: 0.75 to 0.94), while the indicated SEL program led to RR of 0.73 (95% CI: 0.57 to 0.93). A summary of studies included in the meta-analysis of intervention effect sizes for the universal and indicated SEL programs is presented in Appendix Table 2 and Appendix Table 3.<br><br>The intervention effect size for suicide mortality was based on a meta-analysis of three studies identified as part of a review[17] conducted by WHO to update mhGAP evidence-based guidelines.[18] It found that school-based

Section	Model parameter	Description
		SEL programs produced a RR of 0.65 (95% CI: 0.51 to 0.83) in reducing suicide attempts after 1-year follow-up. Equations (23) to (28) in Appendix S5 present a mathematical proof demonstrating how post-intervention reductions in suicide mortality can be estimated using an effect size involving suicide attempts. To summarise, if the case fatality proportion of suicide attempts is assumed to be constant pre- and post- intervention, then it is possible to directly apply the effect size for suicide attempts to suicide mortality. In response to feedback provided by the international expert panel, a decision was made to adopt a lower intervention effect size by using the upper confidence interval bound as the point estimate. Both universal and indicated school-based SEL programs were subsequently estimated to produce a RR of 0.83 (95% CI: 0.70 to 0.99) in reducing suicide mortality after 1-year follow-up.
	Suicide mortality	Data on overall suicide rates were obtained for the year 2017 by country, age and sex from GBD 2017.[19]<br><br>Overall suicide rates occurring between the years 2018 and 2117 were estimated by accounting for historical trends in suicide rates. These trends were based on GBD 2017 data on the average year-on-year percentage change in suicide rates, as observed in each country between the years 1990 and 2017.[19] For example, the average year-on-year percentage change in the Indian suicide rate between 1990 and 2017 was -0·46% (SD: 1·98) in males and -1·46% (SD: 2·53) in females. Parametric bootstrapping, using a normal distribution, was performed on the average year-on-year percentage change in suicide rates (calculated above) to estimate the percentage change in suicide rates occurring in each year between 2018 and 2117. For example, using parametric bootstrapping we calculate estimates for the year-on-year change of -2·4% in year one, +2·8% in year two and -1·1% in year three. If the suicide rate for Indian females aged 15 to 19 years was 18·9 deaths per 100,000 during 2017, then the suicide rate would be: 18·4 per 100,000 in year one (18·9 × [1 - 0·024]); 19·0 per 100,000 in year two (18·4 × [1 + 0·028]); and 18·7 per 100,000 in year three (19·0 × [1 - 0·011]).
	Relative risk of suicide mortality among people with depression/anxiety	The relative risk (RR) of suicide mortality among people with depression/anxiety is required to estimate the population attributable fraction used to split the overall suicide rate into: (1) suicides attributable to people with depression/anxiety; and (2) suicides attributable to people at risk of depression/anxiety [see Equations (7) to (13) in Appendix S5]. The RR of suicide mortality among people with depression/anxiety was calculated to be 6.5 (95% CI: 2.7 to 12.9). This estimate is the weighted average of the RR of suicide mortality among people with depression and the RR of suicide mortality among people with anxiety. In GBD 2017,[19] the RR of suicide mortality among people with depression is estimated to be 19.9 (95% CI: 9.5 to 41.7),[20] while the RR of suicide mortality among people with depression is zero. World Mental Health Survey data on the prevalence of depression and anxiety[21,22] were used to the weight the RR of suicide mortality for depression and anxiety, respectively (see Appendix Table 4).
	Epidemiology of depression	Data on the incidence, prevalence, case fatality and remission for depression were obtained for the year 2017 by country, age and sex from GBD 2017.[19]
	Epidemiology of anxiety	Data on the incidence, prevalence, case fatality and remission for anxiety were obtained for the year 2017 by country, age and sex from GBD 2017.[19]
	Epidemiology of depression and/or anxiety	The incidence, prevalence, case fatality and remission for depression and/or anxiety (i.e., depression/anxiety) were estimated by combining separate epidemiological data for depression and anxiety using the dependent comorbidity method.[23] See Appendix S6 for further details.

Section	Model parameter	Description
	Disability-Adjusted Life Years (DALYs)	Intervention health impacts were summarised using the DALY measure, which is the sum of the total Years of Life Lost (YLL) and Years Lived with Disability (YLDs) in the population. The estimation of YLLs and YLDs are described in the following rows.
	Years of Life Lost (YLLs)	YLLs were estimated for each age-sex cohort by: taking the number of all deaths experienced by a cohort in a particular year; and multiplying this by the potential years of life lost. The potential years of life lost were, in turn, calculated as the lowest value of either: the difference between the current age of the cohort and the average life expectancy in the country; or the difference between the current age of the cohort and the remaining time before the end of the 100-year model time horizon. For example, a person dying at age 50 in the baseline year of 2017 would lead to 30 YLLs (if the average life expectancy was 80 years); while a person dying at age 50 in the year 2117 (i.e., the final year over the 100-year time horizon) would only lead to one YLL. Overall, total YLLs in the intervention scenario will be lower than total YLLs in the comparator scenario due to the reduction in suicide mortality. Data on the average life expectancy of males and females in each country were obtained from GBD 2017.[19]<br><br>YLL estimates were adjusted to account for differing levels of background morbidity experienced by different age groups. For example, YLLs among older individuals (e.g., those aged 65+ years) will be lower as they experience greater background morbidity due to age-related chronic diseases (e.g., cardiovascular disease, cancers, stroke, dementia) when compared to younger individuals. YLLs were adjusted by multiplying each age-specific potential year of life lost by: (1 – pYLD), where pYLD is the age-specific prevalent YLD rate due to all causes of disease (i.e., background morbidity). Data on age-specific pYLD rates due to all causes of disease were obtained from GBD 2017.[19]
	Years Lived with Disability (YLDs)	YLDs were estimated for each age-sex cohort by: calculating the total number of depression/anxiety cases experienced by a cohort in a particular year; and multiplying this by the relevant GBD 2017 disability weight.<br><br>The GBD 2017 disability weight was 0.254 (SE: 0.032) for depression and 0.146 (SE: 0.019) for anxiety.[19] The following multiplicative function was used to calculate the disability weight for comorbid depression and anxiety, which was estimated to be 0.362 (SE: 0.031).<br><br>$$DW_{Depression+Anxiety} = 1 - \left(1 - DW_{Depression}\right)\left(1 - DW_{Anxiety}\right)$$<br><br>The combined disability weight for depression and/or anxiety was calculated as the weighted average of disability weights for: depression only (0.254, SE: 0.032); anxiety only (0.146, SE: 0.019); and comorbid depression and anxiety (0.362, SE: 0.031). Data from the World Mental Health Surveys[21,22] were used to estimate the relative distribution of the prevalence for: depression only (15.6%, SE: 0.53); anxiety only (62.5%, SE: 0.86); and comorbid depression and anxiety (21.9%, SE: 0.57). The combined disability weight for depression/anxiety was subsequently estimated to be 0.203 (SE: 0.018).<br><br>YLD estimates were adjusted to account for differing levels of background morbidity experienced by different age groups. For example, YLDs among older individuals (e.g., those aged 65+ years) will be higher as they experience greater background morbidity due to age-related chronic diseases (e.g., cancers, stroke, dementia) when compared to younger individuals. Data on prevalent YLD rates due to all causes of disease (i.e., background morbidity) were obtained from GBD 2017.[19] A multiplicative function was used to combine age-specific YLD rates due to depression/anxiety with age-specific YLD rates due to other causes of disease.

Appendix Table 2 **Summary of studies included in the meta-analysis of intervention effect sizes for the universal SEL program**

Author & year	Study design	Name of intervention	Treatment description with target group/recipient	Control description	Country	Female (%)	Total sample (n) at start of study	Intervention Group (n)	Control Group (n)	Mean age (mean, sd) or age range	Source
Bond et al. (2004), Patton et al. (2006)	cRCT	The Gatehouse Project	Gatehouse Project intervention, whole-school focus, tailored to some extent to schools' needs and including consultation on policies, also included a curriculum with 8th graders delivering on average 20 lessons over 10 weeks	Treatment as usual	Australia	53·2%	2678	1335	1343	18·75, 1·03	[24,25]
Buttigieg et al. (2015)	cRCT	Resilient Families	Intervention + parent education: A comprehensive school-based, weekly 10-session intervention with satellite sessions for parents about protective factors	No treatment	Australia	56·4%	2027	967	1060	16·80, 0·6	[26]

Author & year	Study design	Name of intervention	Treatment description with target group/recipient	Control description	Country	Female (%)	Total sample (n) at start of study	Intervention Group (n)	Control Group (n)	Mean age (mean, sd) or age range	Source
Butzer et al. (2017)	cRCT	Kripalu Yoga in the Schools (KYIS), expanded	Yoga-based curriculum in physical education classes in 32 sessions over 24 weeks, combining physical exercises with socioemotional lessons and mindfulness practice	Treatment as usual: physical education class	United States	63·2%	211	117	94	14·00, n/s	[27]
Horowitz et al. (2007)	RCT	CB / IPT-AST	Cognitive-behavioral intervention program delivered over eight sessions in school	Treatment as usual: health classes	United States	54·0%	380	211	169	14·70, n/s	[28]
Khalsa et al. (2012)	cRCT	Yoga Ed	A yoga intervention implemented during physical education classes 2-3 times per week over 11 weeks, including postures, breathing, visualization, and games	Treatment as usual: physical education class	United States	42·1%	121	74	47	13·20, 0·5	[29]

**Author & year**	**Study design**	**Name of intervention**	**Treatment description with target group/recipient**	**Control description**	**Country**	**Female (%)**	**Total sample (n) at start of study**	**Intervention Group (n)**	**Control Group (n)**	**Mean age (mean, sd) or age range**	**Source**
Kindt et al. (2014)	cRCT	Op Volle Kracht (OVK)	OVK intervention uses CBT principles to teach adolescents coping skills and problem-solving in classroom setting over 16 weekly sessions	Treatment as usual	Netherlands	52·3%	1343	667	676	T = 11·99, 0·34; C = 11·99, 0·33	[30]
Kraag et al. (2009)	cRCT	Learn Young, Learn Fair	Learn Young, Learn Fair intervention providing coping skills and mental health literacy over 8 weekly sessions with 5 boosters	Wait-list condition	Netherlands	49·9%	1467	693	744	16·70, 0·51	[31]
Langer et al. (2017)	RCT	Mindfulness in Schools Project (MiSP)	Mindfulness meditation class delivered in class over 8 weekly sessions	Wait-list condition	Chile	52·3%	88	41	47	10·91, 0·86	[32]

Author & year	Study design	Name of intervention	Treatment description with target group/recipient	Control description	Country	Female (%)	Total sample (n) at start of study	Intervention Group (n)	Control Group (n)	Mean age (mean, sd) or age range	Source
Leventhal et al. (2015)	cRCT	Girls First Resilience Curriculum (RC)	Girls First RC teaches resilience skills, focused on psychosocial assets and wellbeing; this study uses data from RC condition and RC+ HC (health curriculum) combined condition from a larger study, delivered over 23 weeks	Treatment as usual (school)	India	100·0%	2665	1802	863	6th grade	[33]
Melnyk et al. (2013, 2015)	cRCT	COPE (Creating Opportunities for Personal Empowerment) Healthy Lifestyles TEEN (Thinking, Emotions, Exercise, Nutrition) Program	COPE intervention over 15 weekly sessions to equip students with skills to combat obesity and reduce mental health disorders	Healthy Teens, a 15-week attention-control program to complement the intervention period	United States	51·6%	779	358	421	10-12; 13-16	[34,35]

Author & year	Study design	Name of intervention	Treatment description with target group/recipient	Control description	Country	Female (%)	Total sample (n) at start of study	Intervention Group (n)	Control Group (n)	Mean age (mean, sd) or age range	Source
Mendelson et al. (2010)	cRCT	(no name)	Mindfulness and yoga program to promote positive mental health and relieve stress, delivered four times weekly in class over 12 weeks	No treatment	United States	60·8%	97	51	46	14·74, 0·73	[36]
Merry et al· (2004)	RCT	Resourceful Adolescent Program (RAP)-Kiwi	Intervention with cognitive behavioral and interpersonal therapy models delivered in school over 11 sessions	Placebo: similar program, without cognitive components	New Zealand	51·6%	364	192	172	16·43, n/s	[37]
Nash (2007)	RCT	Empower Youth Program	Empower Youth Program + Usual School Services: Holistic Empower Youth Program combines physical, psychosocial, emotional, intellectual, and spiritual aspects of wellbeing, with an aim to reduce risk and boost healthy behaviors, delivered over 9 weekly sessions	Treatment as usual: Usual School Services	United States	72·5%	40	21	19	13·08, 0·58	[38]

Author & year	Study design	Name of intervention	Treatment description with target group/recipient	Control description	Country	Female (%)	Total sample (n) at start of study	Intervention Group (n)	Control Group (n)	Mean age (mean, sd) or age range	Source
Noggle et al. (2012)	RCT	(no name)	Yoga curriculum delivered in physical education class over 10 weeks, 2-3 times weekly, including physical exercises, breathing, meditation techniques, as well as discussion of a theme focused on life skills	Treatment as usual: physical education class	United States	56·9%	51	36	15	K, 4th, 8th grade	[39]
Park et al. (2000), Mason et al. (2007)	RCT	Preparing for the Drug Free Years (PDFY)	PDFY intervention works with families and parents to prevent and reduce substance use in adolescents, specifically targeting communication and resistance skills, over 5 weekly sessions	Informational leaflets about adolescent development delivered in mail	United States	52·0%	429	221	208	14·41, 1·20	[40,41]
Pössel et al. (2004)	RCT	LISA-T	Received 10 sessions of the LISA-T program at school	Received treatment as usual	Germany	47·8%	347	200	147	12 to 13	[42]

Author & year	Study design	Name of intervention	Treatment description with target group/recipient	Control description	Country	Female (%)	Total sample (n) at start of study	Intervention Group (n)	Control Group (n)	Mean age (mean, sd) or age range	Source
Pössel et al. (2013)	RCT	LARS&LISA	Cognitive-behavioral intervention based on a social information processing model, employs elements of CBT to prevent negative thinking and depression and tying thoughts to behaviors, and increase social competence, delivered over 10 weekly sessions	Treatment as usual: typical Wellness class curriculum	United States	62·7%	518	341	177	13·00, 0·40	[43]
Quayle et al. (2001)	RCT	Optimism and Life Skills Intervention (adapted version of Penn Depression Prevention Program)	Optimism and Lifeskills Program, a social and cognitive based intervention delivered over 8 weekly sessions in class	Usual educational program	Australia	100·0%	47	24	23	12 to 13	[44]

Author & year	Study design	Name of intervention	Treatment description with target group/recipient	Control description	Country	Female (%)	Total sample (n) at start of study	Intervention Group (n)	Control Group (n)	Mean age (mean, sd) or age range	Source
Rivet-Duval et al. (2011)	RCT	RAP-A (Resourceful Adolescent Program-Adolescent version)	Universal prevention program based on cognitive-behavioural and interpersonal therapies delivered in 11 one-hour weekly sessions to groups		Mauritius	50·0%	160	80	80	10 to 17	[45]
Roberts et al. (2010)	cRCT	Aussie Optimism Program	Received 20 lessons of the Aussie Optimism program at school	Received usual health education lessons at school	Australia	54·4%	496	274	222	12·30, n/s	[46]
Rose et al. (2014)	cRCT	RAP (Resourceful Adolescent Program); PIR (Peer Interpersonal Relatedness)	RAP-PIR: RAP, a depression reduction and resourcefulness intervention, delivered in class over 11 weekly sessions; followed by PIR, a social skills intervention, delivered in class over 9 weekly sessions	No treatment	Australia	44·0%	210	130	80	12·30, n/s	[47]

**Author & year**	**Study design**	**Name of intervention**	**Treatment description with target group/recipient**	**Control description**	**Country**	**Female (%)**	**Total sample (n) at start of study**	**Intervention Group (n)**	**Control Group (n)**	**Mean age (mean, sd) or age range**	**Source**
Ruini et al. (2007)	RCT	(no name)	Received CBT-based intervention 4 two-hour sessions in the class every other week	Received well-being therapy intervention 4 two-hour sessions in the class every other week	Italy	46·0%	111	54	57	18·70, 0·8	[48]
Sawyer et al. (2010a, 2010b)	RCT	Beyondblue	Receives 10 sessions of Beyondblue at school	No treatment	Australia	53·0%	5,633	3,040	2,593	16·30, 1·20	[49,50]
Schilling et al. (2016)	cRCT	Signs of Suicide (SOS)	SOS multimethod intervention that teaches students to recognize signs of suicide and gain more knowledge about strategies to link to support, delivered in a classroom setting over 2 days	Wait-list condition	United States	41·7%	1575	729	555	12·99, 1·17	[51]

Author & year	Study design	Name of intervention	Treatment description with target group/recipient	Control description	Country	Female (%)	Total sample (n) at start of study	Intervention Group (n)	Control Group (n)	Mean age (mean, sd) or age range	Source
Sibinga et al. (2013)	RCT	Mindfulness-Based Stress Reduction (MBSR)	13 weeks of mindfulness training at school	Education in nutrition, exercise, body systems, adolescence, and puberty at school	United States	0·0%	41	22	19	13·00, n/s	[52]
Sibinga et al. (2016)	RCT	Mindfulness-Based Stress Reduction (MBSR)	12 weeks of mindfulness training in class at school	Education in nutrition, exercise, body systems, adolescence, and puberty at school	United States	50·7%	298	158	140	T = 11·25, n/s; C = 11·24, n/s	[53]
Stallard et al. (2012, 2013)	cRCT	Resourceful Adolescent Program (RAP) - United Kingdom	RAP-UK: CBT-based and IPT-based program delivered over 9 weekly or fortnightly sessions in class, to whole classes, by external facilitators	Usual PSHE: personal social health education sessions delivered by teachers	United Kingdom	49·0%	5030	3426	1604	12 to 16	[54,55]

Author & year	Study design	Name of intervention	Treatment description with target group/recipient	Control description	Country	Female (%)	Total sample (n) at start of study	Intervention Group (n)	Control Group (n)	Mean age (mean, sd) or age range	Source
Stallard et al. (2014, 2015)	cRCT	FRIENDS	"Health-led" FRIENDS: Whole-class delivery over nine 60-minute weekly sessions delivered by 2 health staff leaders with a teacher (3 people per class)	Treatment as usual: Control schools participated in the usual health education provided by the school	United Kingdom	51·4%	1362	961	401	14·72, 1·57	[56,57]
Tak et al. (2014, 2016)	cRCT	Op Volle Kracht (OVK)	Received 16 sessions of OKV and two boosters sessions at school	No treatment	Netherlands	47·3%	1341	634	707	7th-8th grade	[58,59]

Abbreviations: C - control; cRCT - cluster randomised controlled trial; K - kindergarten; n/s - not stated; RCT - randomised controlled trial; T - Treatment.

Appendix Table 3 Summary of studies included in the meta-analysis of intervention effect sizes for the indicated SEL program

Author & year	Study design	Name of intervention	Treatment description with target group/recipient	Control description	Country	Study population description	How were the participants screened in to the intervention?	Female (%)	Total sample (n) at start of study	Mean age (mean, sd) or age range	Source
Arnarson & Craighead (2009); Arnarson & Craighead (2011)	RCT	n/s	14 group sessions based on the developmental psychosocial model of enhancement of resilience to factors associated with the occurrence of mood disorders in adolescents at risk for developing a mental health diagnosis	Treatment as usual	Iceland	Adolescents judged to be 'at risk' for depression, reporting the presence of depressive symptoms or a negative attributional style	A score between the 75th and 90th percentile on Children's Depression Inventory or at the 75th percentile or higher on the negative composite of the Children's Attributional Style Questionnaire	52.0%	171	14-15	[60,61]
Balle & Tortella-Feliu (2010)	RCT	n/s	6 group sessions to reduce anxiety and depression in school children with high anxiety sensitivity	Wait-list control	Spain	School children with high anxiety sensitivity	Scoring over 80th percentile in the Children Anxiety Sensitivity Index	n/s	92	11-17	[62]
Bella-Awusah et al. (2016)	RCT	n/s	A culturally-relevant 5 session school-based group cognitive behavioural therapy programme for depressed adolescents	Wait-list control	Nigeria	School adolescents reporting depressive symptoms	Score of 18 or above on the Beck Depression Inventory	70.0%	40	15.6, 0.9	[63]

Author & year	Study design	Name of intervention	Treatment description with target group/recipient	Control description	Country	Study population description	How were the participants screened in to the intervention?	Female (%)	Total sample (n) at start of study	Mean age (mean, sd) or age range	Source
Berry & Hunt (2009)	RCT	Confident Kids Program	8 groups sessions focused on reducing individual vulenrability to bullying for anxious adolescent boys experiencing bullying at school as well as parallel sessions for their parents	Wait-list control	Australia	School children with anxiety and who have been bullied and their parents	Anxiety score of at least one standard deviation above the population mean on any subscale of the Screen for Child and Anxiety Related Emotional Disorders and a definitely disabling and disturbing rating on the Bullying Incidence Scale	n/a	46	13.04, 0.79	[64]
Dobson et al. (2010)	Factorial RCT	Adolescent Coping with Stress Course	15 CBT sessions to prevent the development of depression in high-risk school children	Active control (15 groups sessions discussing topics of interest to school children)	Canada	School children with elevated scores of depression	Scoring 24 or above on the Center for Epidemiological Studies–Depression Scale	69.5%	46	15.26, 1.1	[65]
Gaete et al. (2016)	RCT	Yo Pienso Siento Actuo - R (I Think Feel Act - Revised)	8 group CBT sessions with low-income school children at risk for depression	Treatment as usual	Chile	School children from a low-income area experiencing symtproms of depression	Having a Beck Depression Inventory score ≥10 (among boys) and ≥15 (among girls).	50.3%	342	15.9, 0.9	[66]

**Author & year**	**Study design**	**Name of intervention**	**Treatment description with target group/recipient**	**Control description**	**Country**	**Study population description**	**How were the participants screened in to the intervention?**	**Female (%)**	**Total sample (n) at start of study**	**Mean age (mean, sd) or age range**	**Source**
Gau et al. (2012)	RCT	n/s	6 group brief cognitive behavioral depression prevention program for adolescents with elevated depressive symptoms	Active control (received a brochure describing depression symptoms and treatment options)	United States	School students experiencing sadness	Score of 20 or above on the Center for Epidemiologic Studies-Depression scale	58.0%	173	15.5, 1.2	[67]
Hunt et al. (2009)	cRCT	FRIENDS	10 weekly cognitive-behavioural group sessions and two booster sessions for anxious children as well as two session for their parents	Monitoring control	Australia	Adolescents reporting anxiety symptoms and adolescents seen as having anxiety symptoms by their teachers as well as their parents	Score above 11 on the Revised Children's Manifest Anxiety Scale or nomination from teacher as displaying anxiety	n/s	260	11-13	[68]

Author & year	Study design	Name of intervention	Treatment description with target group/recipient	Control description	Country	Study population description	How were the participants screened in to the intervention?	Female (%)	Total sample (n) at start of study	Mean age (mean, sd) or age range	Source
Jacob & De Guzman (2016)	RCT	Taking in the Good	8 group session based-Bibliotherapy Intervention Program for depressed female adolescents. An innovative type of psychotherapy treatment program aimed to build up their inner strengths	Non-treatment group	Phillippines	Depressed adolescent females	High scores in depression in the pre-test: Beck Depression Inventory-II (BDI-II >14), Asian Adolescent Depression Scale (AADS >61) and Kutcher Adolescent Depression Scale-11 (KADS–11 >12).	100.0%	30	13-16	[69]
Kwok et al. (2016)	RCT	Live a Positive Life	Positive psychology group-based intervention program aimed at decreasing depression and increasing life satisfaction among primary school students	No-treatment control	Hong Kong	Primary school students from 5 primary schools with marginal or probable clinical cases of depression or anxiety.	A score from 9 to 11 on the Chinese Hospital Anxiety and Depression Scale	47.1%	68	10.4, 1.84	[70]

Author & year	Study design	Name of intervention	Treatment description with target group/recipient	Control description	Country	Study population description	How were the participants screened in to the intervention?	Female (%)	Total sample (n) at start of study	Mean age (mean, sd) or age range	Source
Livheim et al. (2015)	RCT	ACT (Acceptance and Commitment Therapy) Experiential Adolescent Group (Australian sample)	Brief group intervention based on the principles of Acceptance and Commitment Therapy for school children with psychosocial problems	Treatment as usual (monitoring support from the school counsellor)	Australia	Adolescents screened for depression symptoms in 5 schools	School counsellor/welfare coordinators nominated students who were experiencing mild to moderate depressive symptoms	87.9%	66	14.6, 1.03	[71]
Livheim et al. (2015)	RCT	ACT (Acceptance and Commitment Therapy) Experiential Adolescent Group (Swedish sample)	Brief group intervention based on the principles of Acceptance and Commitment Therapy for school children with psychosocial problems	Treatment as usual (individual support by the school nurse)	Sweden	Adolescents screened for anxiety symptoms in a school setting	Scoring above the 80th percentile on scales measuring psychological problems (Strengths and Difficulties Questionnaire, the Perceived Stress Scale, and the General Health Questionnaire).	71.9%	32	14-15	[71]

Author & year	Study design	Name of intervention	Treatment description with target group/recipient	Control description	Country	Study population description	How were the participants screened in to the intervention?	Female (%)	Total sample (n) at start of study	Mean age (mean, sd) or age range	Source
Martinsen et al. (2019)	cRCT	EMOTION, Coping Kids Managing Anxiety and Depression	20 child sessions and 7 parent sessions of a CBT programme targetting disturbances in cognition, affect regulation, problem solving, and coping skills among youth at risk for emotional difficulties	Normal contact with school health nurse/physician	Norway	Adolescents at risk for emotional difficulties	Child scoring 1 SD above mean on measure of anxiety or/and child scoring 1 SD above mean on measures of depression: MASC-Child Girls: IC - 4 6 + 1 (SD), Boys: IC - 39 + 15 (SD); SMFQ-Child Boys/girls: IC - 3.8 + 3.6 (SD)	42.0% (of analysed sample)	873	10.1, 0.90	[72]
McCarty et al. (2011)	RCT	Positive Thoughts and Actions	12-week program with a parent-component aimed to address depressive symptoms using three intervention targets—coping, cognitive style, and parent-child communication	School-as-usual	United States	Middle school students with elevated depressive symptoms from 4 public schools	Score of higher than 14 (top 25%) on the Mood and Feelings Questionnaire	50.7%	67	12.98, 0.38	[73]

**Author & year**	**Study design**	**Name of intervention**	**Treatment description with target group/recipient**	**Control description**	**Country**	**Study population description**	**How were the participants screened in to the intervention?**	**Female (%)**	**Total sample (n) at start of study**	**Mean age (mean, sd) or age range**	**Source**
Noel et al. (2013)	RCT	Talk 'n' Time	12 weekly sessions of a peer-led CBT school-based intervention among female adolescents experiencing depressive symptoms	No intervention	United States	Female adolescents experiencing symptoms of depression	Scored 10 or above on the Center for Epidemiological Studies Depression Scale (CES-D), or endorsed questions 1 or 3 (depressed mood or anhedonia) as moderate or severe for the current month on the Kiddie-Schedule for Affective Disorders and Schizophrenia (K-SADS)	100%	34	13.76, 1.02	[74]

Author & year	Study design	Name of intervention	Treatment description with target group/recipient	Control description	Country	Study population description	How were the participants screened in to the intervention?	Female (%)	Total sample (n) at start of study	Mean age (mean, sd) or age range	Source
Poppelaars et al. (2016)	Factorial RCT	Op Volle Kracht (OVK) / SPARX	Three intervention arms: 1) 8 lessons of a CBT-based depression prevention intervention (OVK) for Dutch adolescent girls with elevated depressive symptoms; 2) 7 levels of a CBT-based digital game (SPARX) for Dutch adolescent girls with elevated depressive symptoms; 3) Combined intervention	Monitoring control	Netherlands	Adolescent Dutch girls with elevated depressive symptoms	Score at or above the 70th percentile on depressive symptoms within the sample (Reynolds Adolescent Depression Scale (RADS-2) score of 59 or more, n = 297)	100%	208	13.35, 0.71	[75]
Puskar et al. (2003)	RCT	Teaching Kids to Cope	10 week group-based, CBT intervention on rural adolescents experiencing depressive symptoms	Usual care	United States	Rural adolescents experiencing depressive symptoms	Score in the mid-range (at least 60) on the Reynolds Adolescent Depression Scale (RADS)	82.0%	89	16.0, 0.95	[76]

Author & year	Study design	Name of intervention	Treatment description with target group/recipient	Control description	Country	Study population description	How were the participants screened in to the intervention?	Female (%)	Total sample (n) at start of study	Mean age (mean, sd) or age range	Source
Pybis et al. (2015)	RCT	n/s	10 sessions of school-based humanistic counselling for adolescents experiencing psychological distress	Wait-list control	United Kingdom	Adolescents experiencing psychological distress	Score of five or more on the Emotional Symptoms subscale of the self-reported Strengths and Difficulties Questionnaire (SDQ-ES)	71.4%	42	14.5, 1.35	[77]
Roberts et al. (2003); Roberts et al. (2004)	cRCT	Penn Prevention Program (PPP)	12 session CBT programme aimed at reducing depressive and anxious symptoms in rural adolescents with elevated depressive symptoms	Usual health education classes and symptom monitoring	Australia	Adolescents with elevated depressive symptoms	Participating children in each class were ranked ordered using their Child Depression Inventory (CDI) scores, and 13 children with the highest scores from each class were invited to participate. In classes with 13 or fewer students, all children were invited. Sixty-one percent of children with CDI scores ranging from 1 to 37 were invited to participate.	49.7%	189	11.89, 0.33	[78,79]

Author & year	Study design	Name of intervention	Treatment description with target group/recipient	Control description	Country	Study population description	How were the participants screened in to the intervention?	Female (%)	Total sample (n) at start of study	Mean age (mean, sd) or age range	Source
Rohde et al. (2014a); Rohde et al. (2015)	RCT	n/s	Two intervention arms: 1) Six sessions of group CBT for adolescents with depressive symptoms; 2) Minimal contact bibliotherapy (i.e. provided with copies of a self-help book) for adolescents with depressive symptoms	Educational brochure (provided with NIMH brochure describing depression and treatment options)	United States	Adolescents with symptoms of depression	One-page self-administered screening measure assessing depressive symptoms based on the Center for Epidemiologic Studies-Depression Scale, students who endorsed two or more symptoms were encouraged to participate.	68.0%	378	15.5, 1.2	[80,81]

Author & year	Study design	Name of intervention	Treatment description with target group/recipient	Control description	Country	Study population description	How were the participants screened in to the intervention?	Female (%)	Total sample (n) at start of study	Mean age (mean, sd) or age range	Source
Saelid & Nordahl (2017)	RCT	n/s	Two intervention arms: 1) Three individual rational emotive behaviour therapy (REBT) sessions for adolescents with subclinical levels of anxiety and depression; 2) Three individual attentional placebo (ATP) sessions in which participants could ventilate and express their feelings for adolescents with subclinical levels of anxiety and depression	No sessions	Norway	Adolescents with subclinical levels of anxiety and depression	Hospital Anxiety and Depression Scale (HADS), on which between 8 and 13 should suggest a subclinical, but elevated, level of symptoms	n/s	62	16-19	[82]

Author & year	Study design	Name of intervention	Treatment description with target group/recipient	Control description	Country	Study population description	How were the participants screened in to the intervention?	Female (%)	Total sample (n) at start of study	Mean age (mean, sd) or age range	Source
Sheffield et al. (2006)	cRCT	n/s	Two intervention arms: 1) Indicated only including 8-week indicated CBT program (small group format, longer sessions) focussing on cognitive restructuring, problem solving skills, and interpersonal skills training for adolescents with elevated symptoms of depression; 2) Universal + Indicated program starting with 8-week universal CBT program focussing on cognitive restructuring and problem solving skills training, followed by 8-week indicated program	No intervention	Australia	Adolescents with elevated symptoms of depression	High-symptom students were selected as those scoring in the top 20% on the combined scores (sum of standardized scores) on the Children's Depression Inventory (CDI) and the Center for Epidemiologic Studies—Depression Scale (CES-D)	69.0%	521	14.34, 0.46	[83]

Author & year	Study design	Name of intervention	Treatment description with target group/recipient	Control description	Country	Study population description	How were the participants screened in to the intervention?	Female (%)	Total sample (n) at start of study	Mean age (mean, sd) or age range	Source
Smith et al. (2015)	RCT	Stressbusters	8 week computerised-CBT (C-CBT) programme for adolescents with mild to moderate depression	Wait-list control	United Kingdom	Adolesents with mild to moderate depression	Scored 20 or greater on the Mood and Feelings Questionnaire-Child Report (MFQ-C)	n/s	112	12-16	[84]
Spence et al. (2003)	RCT	Problem Solving for Life	8 sessions of cognitive restructuring and problem solving skills training for universal and high risk samples of adolescents	Monitoring control	Australia	All adolescents and high/low-risk adolescents	Students were classified as high risk on the basis of scores greater than or equal to 13 on the Beck Depression Inventory (BDI). Those who did not report elevated BDI scores were included in the high-risk group on the basis of positive responses to specific dysthymia questions or a positive response to the suicide question on the BDI. Students whose BDI scores were less than 13 were categorized as low-risk status.	51.5% - here, n/s for high-risk	399	12.87, 0.54	[85]

Author & year	Study design	Name of intervention	Treatment description with target group/recipient	Control description	Country	Study population description	How were the participants screened in to the intervention?	Female (%)	Total sample (n) at start of study	Mean age (mean, sd) or age range	Source
Stallard et al. (2013)	cRCT	The Resourceful Adolescent Programme	Two intervention arms: 1) Classroom-based cognitive-behavioural therapy (CBT) to reduce symptoms of depression in high-risk adolescents, 2) Attention control (usual school PSHE lessons with additional support from two facilitators)	School-as-usual	United Kingdom	Young people who attended Personal, Social and Health Education lessons at their school	Scores of ≥ 5 on the Short Mood and Feelings Questionnaire	65.7%	1064	14.18, 1.09	[55]
Stasiak et al. (2014)	RCT	The Journey	7 modules of a computerised cognitive behavioural therapy (cCBT) programme for adolescents with symptoms of depression	Attention placebo program with psychoeducational content (CPE); same delivery techniques but different content	New Zealand	Adolescents with symptoms of depression	Raw score of 30 or more on Depression Rating Scale Revised (CDRS-R) or if they scored 76 or above on Reynolds' Adolescent Depression Scale-2nd Edition (RADS-2)	41.2%	34	15.18, 1.48	[86]

Author & year	Study design	Name of intervention	Treatment description with target group/recipient	Control description	Country	Study population description	How were the participants screened in to the intervention?	Female (%)	Total sample (n) at start of study	Mean age (mean, sd) or age range	Source
Wijnhoven et al. (2014)	RCT	Op Volle Kracht (OVK)	8 sessions of a CBT depression prevention programme for adolescent girls with elevated depressive symptoms	Wait-list control	Netherlands	Adolescent girls with elevated depressive symptoms	CDI score of 16 or more	100%	118	13.30, 0.64 (of n=102)	[87]
Woods et al. (2011)	RCT	Kiwi Adolescents Coping with Emotions (ACE)	8 sessions of a cognitive behavioural and psychoeducational based intervention for adolescents experiencing depressive symptoms	Usual care including counselling with school counselors	New Zealand	Adolescents identifying as Maori/Pacific Islander experiencing depressive symptoms	Adolescents scoring over the 'above average' cut off point of 63 on the Children's Depressive Inventory (CDI)	n/s	83	14.0, n/s	[88]

Author & year	Study design	Name of intervention	Treatment description with target group/recipient	Control description	Country	Study population description	How were the participants screened in to the intervention?	Female (%)	Total sample (n) at start of study	Mean age (mean, sd) or age range	Source
Young et al. (2006); Young et al. (2009)	RCT	Teen Talk / Interpersonal Psychotherapy-Adolescent Skills Training (IPT-AST)	2 individual and 8 weekly group sessions of an Interpersonal Psychotherapy Adolescent Skills Training (IPT-AST) for adolescents with elevated depression symptoms	School counselling	United States	Adolescents with elevated symptoms of depression	First screened with CES-D and had to score within range of between and including 16-39; Complete measures and diagnostic interview; at least 2 subthreshold or threshold depression symptoms on the K-SADS-PL and did not meet criteria for a current depressive episode; elevated depressed mood, irritability, or anhedonia was required as was a CGAS score of 61 or higher	85.4%	41	13.4, 1.2	[89,90]

Author & year	Study design	Name of intervention	Treatment description with target group/recipient	Control description	Country	Study population description	How were the participants screened in to the intervention?	Female (%)	Total sample (n) at start of study	Mean age (mean, sd) or age range	Source
Young et al. (2010)	RCT	Interpersonal Psychotherapy-Adolescent Skills Training (IPT-AST)	2 pre-group and 8 group sessions of an Interpersonal Psychotherapy Adolescent Skills Training (IPT-AST) for adolescents with elevated depression symptoms	School counselling	United States	Adolescents with elevated depressive symptoms	First screened with CESD, Adolescents with a CESD score between 16 and 39 were eligible to be approached for the prevention project, those with a score of 40 or higher were seen by the Principal Investigator (PI) to assess clinical severity and determine potential eligibility; adolescents were eligible if they had at least two subthreshold or threshold depression symptoms on the K-SADS-PL, did not meet criteria for a current depressive episode, and had a CGAS score of 61 or higher	59.7%	57	14.51, 0.76	[91]

Author & year	Study design	Name of intervention	Treatment description with target group/recipient	Control description	Country	Study population description	How were the participants screened in to the intervention?	Female (%)	Total sample (n) at start of study	Mean age (mean, sd) or age range	Source
Young et al. (2016); Young et al. (2018)	RCT	Depression Prevention Initiative (DPI)	2 pre-group, 8 group sessions, and an individual midgroup session (involving parents) of an Interpersonal Psychotherapy Adolescent Skills Training (IPT-AST) for adolescents with elevated depression symptoms	School group counselling	United States	Adolescents with elevated depressive symptoms	CES-D score of 16 or higher	66.7%	186	14.01, 1.22	[92,93]

Abbreviations: cRCT - cluster randomised controlled trial; K - kindergarten; n/s - not stated; RCT - randomised controlled trial.

Appendix S4 – Description of the Cochrane conversion method

The Cochrane conversion method described by Lee et al.[16] was used to convert the standardised mean difference (SMD) effect size into a corresponding relative risk (RR) effect size. The Cochrane conversion method encompasses two steps.

The first step involves transforming the SMD into a log odds ratio using Equation (1):

$$ln(OR) = \left(\frac{\pi}{\sqrt{3}}\right).SMD \tag{1}$$

Where: $\ln(OR)$ is the log odds ratio; SMD is the standardised mean difference. This equation assumes that mean scores underlying the intervention and control groups of the SMD metric follow a logistic distribution and have standard deviations that are equal across both groups.[94,95]

The second step transforms the OR estimate into a corresponding RR effect size using Equation (2):

$$RR = \frac{OR}{1 - ACR\,(1 - OR)} \tag{2}$$

Where: RR is the relative risk; OR is the odds ratio; and ACR is the assumed control risk – i.e., the risk of depression/anxiety that is observed in the study control group of a meta-analysis. The ACR was assumed to correspond with the global prevalence of depression/anxiety, which was estimated to be 7.2% (SE: 0.2) in the Global Burden of Disease study 2017.[19] The 95% confidence interval for each predicted RR effect size was estimated by using Monte Carlo simulation to propagate parameter uncertainty around the initial SMD effect size through to the final predicted RR effect size.[96] The SMD effect size was assumed to be normally distributed with: a mean corresponding with the SMD point estimate; and a standard deviation corresponding with the SMD standard error. Overall, it was found that the universal school-based SEL program led to a RR of 0.84 (95% CI: 0.75 to 0.94), while the indicated SEL program led to RR of 0.73 (95% CI: 0.57 to 0.93).

Appendix S5 – Mathematical equations underlying the modelling of health impacts

Introduction

This section provides a mathematical description outlining how input model parameters were used to estimate the epidemiology of depression/anxiety and suicide deaths over the 100-year time horizon of the Markov model developed for this study. The equations described in this section should be interpreted with respect to the state transition diagrams presented below in Appendix Figure 1, Appendix Figure 2 and Appendix Figure 3. The three state transition diagrams are each shown: descriptively (on the left); and using mathematical notation (on the right). It follows that all of the mathematical equations presented in this section adopt the mathematical notation contained in the three state transition diagrams.

Appendix Figure 1 First state transition diagram

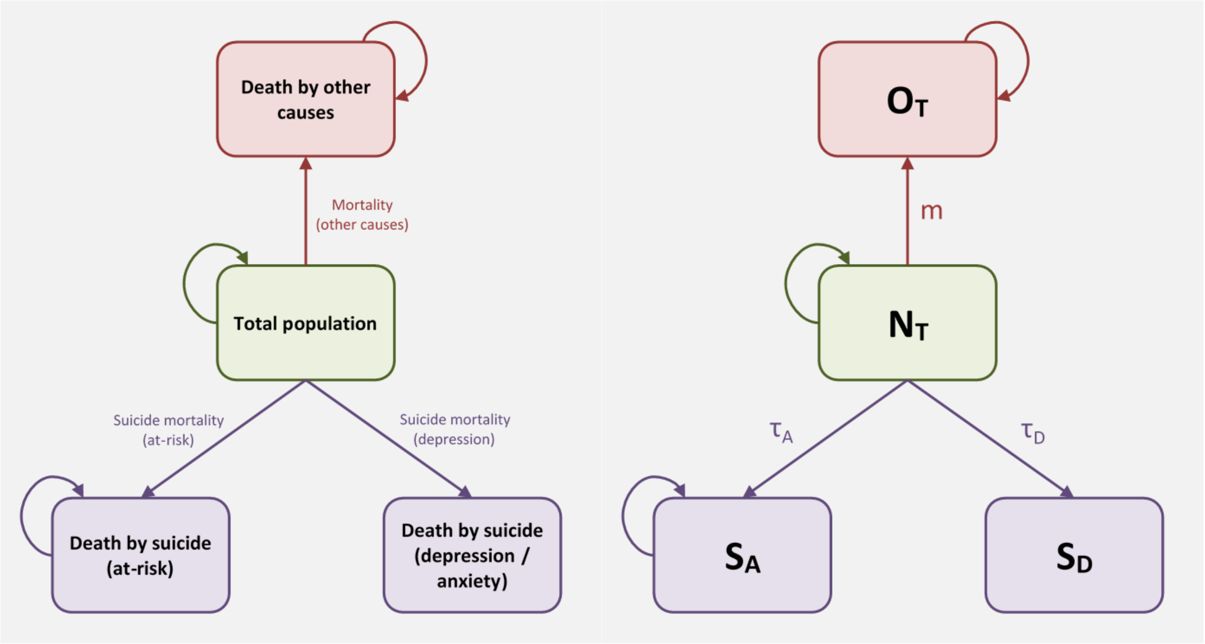

Appendix Figure 2 Second state transition diagram

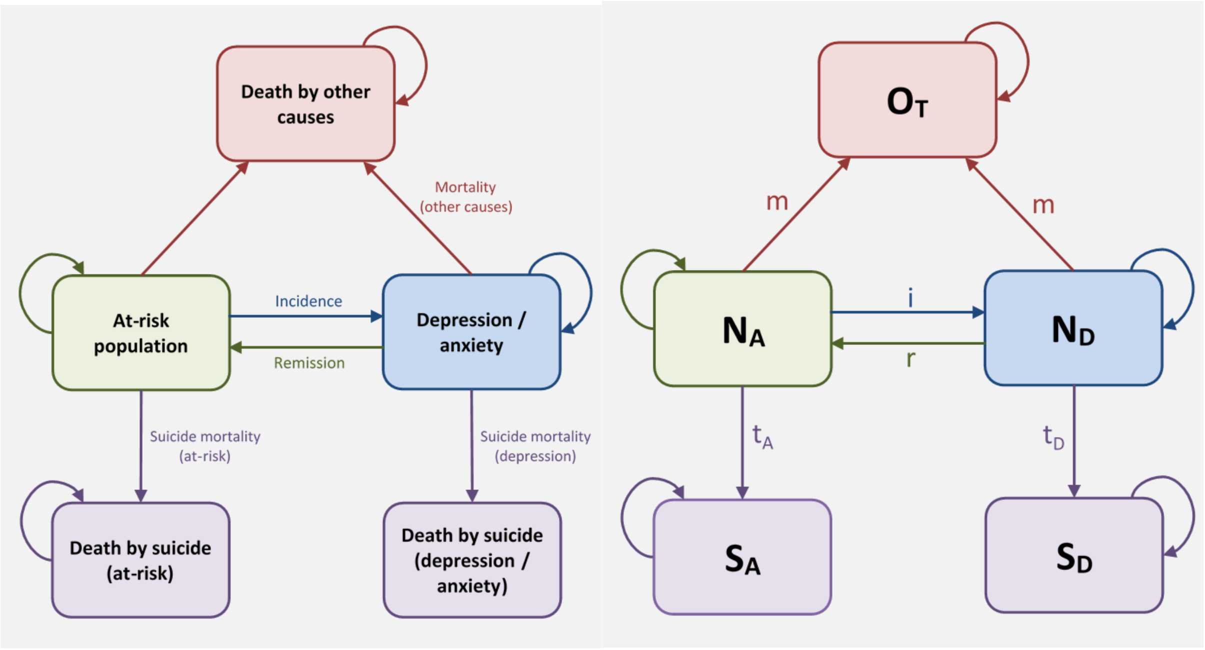

Appendix Figure 3 Third state transition diagram

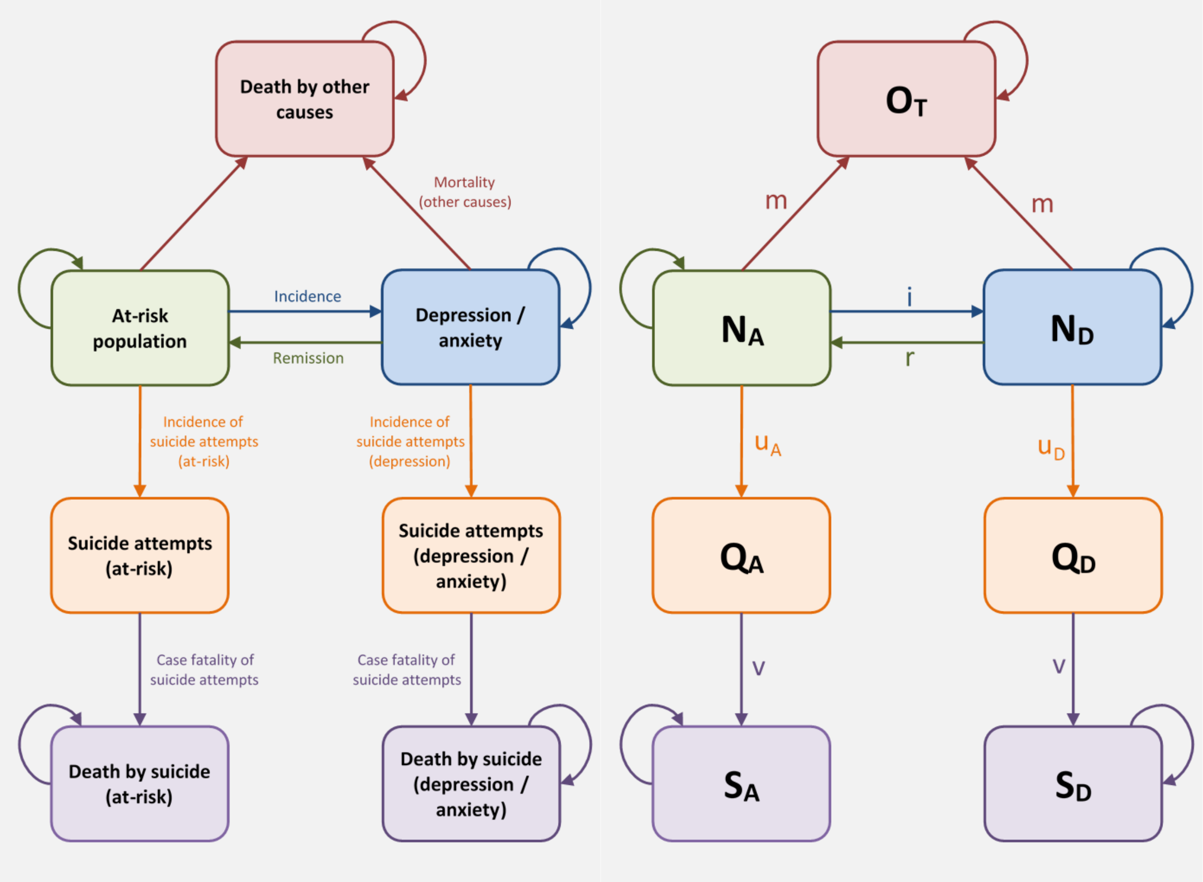

Background equations for each health state

The total number of people in the population is represented by Equation (3):

$$N_T = N_A + N_D \tag{3}$$

Where: N_T is the number of people in the total population; N_A is the number of people at risk of depression/anxiety; and N_D is the number of people with depression/anxiety.

The total number of people who attempt suicide is represented by Equation (4):

$$Q_T = Q_A + Q_D \tag{4}$$

Where: Q_T is the total number of suicide attempts; Q_A is the number of suicide attempts among people at risk of depression/anxiety; and Q_D is the number of suicide attempts among people with depression/anxiety.

The total number of deaths due to suicide is represented by Equation (5):

$$S_T = S_A + S_D \tag{5}$$

Where: S_T is the total number of deaths due to suicide; S_A is the number of deaths due to suicide among people at risk of depression/anxiety; and S_D is the number of deaths due to suicide among people with depression/anxiety.

The total number of deaths due to other causes is represented by Equation (6):

$$O_T = O_A + O_D \tag{6}$$

Where: O_T is the total number of deaths due to other causes; O_A is the number of deaths due to other causes among people at risk of depression/anxiety; and O_D is the number of deaths due to other causes among people with depression/anxiety.

Equations for suicide rates and suicide probabilities

Equation (7) denotes the overall suicide rate:

$$\begin{aligned} \tau_T &= \frac{S_T}{N_T} \\ &= \frac{S_A + S_D}{N_T} \\ &= \frac{S_A}{N_T} + \frac{S_D}{N_T} \\ \tau_T &= \tau_A + \tau_D \end{aligned} \tag{7}$$

Where: τ_T is the overall suicide rate in the population; τ_A is the suicide rate attributable to people at risk of depression/anxiety; and τ_D is the suicide rate attributable to people with depression/anxiety.

Various suicide probabilities are represented by Equations (8), (9) and (10):

$$t_T = \tau_T = \frac{S_T}{N_T} \tag{8}$$

$$t_A = \frac{S_A}{N_A} \tag{9}$$

$$t_D = \frac{S_D}{N_D} \tag{10}$$

Where: t_T is the overall suicide probability in the population; t_A is the suicide probability among people at risk of depression/anxiety; and t_D is the suicide probability among people with depression/anxiety.

Expressing suicide rates in terms of the population attributable fraction

The population attributable fraction is denoted by Equation (11):

$$\begin{aligned} PAF_\tau &= \frac{\tau_D}{\tau_T} \\ PAF_\tau &= \frac{(\tau_T - \tau_A)}{\tau_T} \\ PAF_\tau &= \frac{p(RR_{suicide} - 1)}{p(RR_{suicide} - 1) + 1} \end{aligned} \tag{11}$$

Where: PAF_τ is the population attributable fraction for the overall suicide rate; p is the prevalence of depression/anxiety; and $RR_{suicide}$ is the relative risk of suicide mortality among people with depression/anxiety compared to those without depression/anxiety.

Equations (12) and (13) express the suicide rates τ_D and τ_A in terms of the overall suicide rate in the population (τ_T) and the population attributable fraction.

$$\tau_D = \tau_T \, PAF_\tau \tag{12}$$

$$\tau_A = \tau_T(1 - PAF_\tau) \tag{13}$$

Expressing the suicide probabilities as a function of the suicide rate

To express suicide probabilities as a function of the suicide rate, the equations in the previous sections for t_D, τ_D, t_A and τ_A must be re-arranged as follows.

First, take the suicide probability among people with depression/anxiety (t_D) and express this as a function of the overall suicide rate (τ_T).

$$t_D = \frac{S_D}{N_D}$$

$$S_D = t_D N_D$$

$$\tau_D = \frac{S_D}{N_T}$$

$$S_D = \tau_D N_T$$

$$t_D N_D = \tau_D N_T$$

$$t_D = \tau_D \left(\frac{N_T}{N_D}\right)$$

$$t_D = \tau_D \left(\frac{1}{p}\right)$$

$$t_D = \tau_T \, PAF_\tau \left(\frac{1}{p}\right) \tag{14}$$

Where: p is the prevalence of depression/anxiety (i.e., $\frac{N_D}{N_T} = p$)

Second, take the suicide probability among people at risk of depression/anxiety (t_A) and express this as a function of the overall suicide rate (τ_T).

$$t_A = \frac{S_A}{N_A}$$

$$S_A = t_A N_A$$

$$S_A = \tau_A N_T$$

$$t_A N_A = \tau_A N_T$$

$$t_A = \tau_A \left(\frac{N_T}{N_A}\right)$$

$$t_A = \tau_A \left(\frac{1}{1-p}\right)$$

$$t_A = \tau_T \, (1 - PAF_\tau) \left(\frac{1}{1-p}\right) \tag{15}$$

Where: $1 - p$ is the proportion of people at risk of depression/anxiety (i.e., $\frac{N_A}{N_T} = \frac{N_T - N_D}{N_T} = 1 - \frac{N_D}{N_T} = 1 - p$).

Equation for the mortality probability due to other causes

The mortality probability due to other causes is represented by Equation (16):

$$m = m_T = m_A = m_D \tag{16}$$

Where: m is the other cause mortality probability; m_T is the overall other cause mortality probability in the population; m_A is the other cause mortality probability among people at risk of depression/anxiety; and m_D is the other cause mortality probability among people with depression/anxiety.

Expressing the other cause mortality probability in terms of the all-cause mortality and suicide rates

The overall mortality rate is shown below in Equation (17):

$$\begin{aligned}
\delta_T &= \frac{(S_T + O_T)}{N_T} \\
\delta_T &= \frac{S_T}{N_T} + \frac{O_T}{N_T} \\
\delta_T &= \tau_T + m \\
m &= \delta_T - \tau_T
\end{aligned} \tag{17}$$

Where: δ_T is the overall all-cause mortality rate in the population.

Equations for the incidence probability of suicide attempts

The incidence probability of a suicide attempt among people at risk of depression/anxiety is denoted below by Equation (18):

$$\begin{aligned}
u_D &= \frac{Q_D}{N_D} \\
Q_D &= u_D N_D \\
\\
u_A &= \frac{Q_A}{N_A} \\
Q_A &= u_A N_A \\
\\
u_T &= \frac{Q_T}{N_T} \\
u_T &= \frac{Q_D + Q_A}{N_D + N_A}
\end{aligned} \tag{18}$$

Where: u_D is the incidence probability of suicide attempts among people with depression/anxiety; u_A is the incidence probability of suicide attempts among people at risk of depression/anxiety; u_T is the overall incidence probability of suicide attempts in the population; Q_D is the number of suicide attempts among people with depression/anxiety; Q_A is the number of suicide attempts among people at risk of depression/anxiety; and Q_T is the total number of suicide attempts in the population.

Equations for the case fatality probability following a suicide attempt

The case fatality probability following a suicide attempt is represented by Equation (19):

$$v = v_T = v_A = v_D$$
$$v = \frac{S_T}{Q_T} = \frac{S_A}{Q_A} = \frac{S_D}{Q_D} \qquad (19)$$

Where: v is the case fatality probability following a suicide attempt; v_T is the overall case fatality probability following a suicide attempt in the population; v_A is the case fatality probability following a suicide attempt among people at risk of depression/anxiety; and v_D is the case fatality probability following a suicide attempt among people with depression/anxiety.

Expressing the incidence of suicide attempts in terms of the suicide rate and case fatality of attempts

Equation (20) expresses the incidence probability of suicide attempts in the population (u_T) as a function of the overall suicide rate (τ_T) and the case fatality probability following a suicide attempt (v).

$$\frac{S_T}{N_T} = \frac{S_T}{Q_T} \cdot \frac{Q_T}{N_T}$$
$$t_T = v.u_T$$
$$\tau_T = v.u_T$$
$$u_T = \frac{1}{v}(\tau_T) \qquad (20)$$

Equation (21) expresses the incidence probability of suicide attempts among people at risk of depression/anxiety (u_A) as a function of the overall suicide rate (τ_T) and the case fatality probability following a suicide attempt (v).

$$\frac{S_A}{N_A} = \frac{S_A}{Q_A} \cdot \frac{Q_A}{N_A}$$
$$t_A = v.u_A$$
$$u_A = \frac{1}{v}(t_A)$$
$$u_A = \frac{1}{v}\left[\tau_T\,(1 - PAF_\tau)\left(\frac{1}{1-p}\right)\right] \qquad (21)$$

Equation (22) expresses the incidence probability of suicide attempts among people with depression/anxiety (u_D) as a function of the overall suicide rate attributable (τ_T) and the case fatality probability following a suicide attempt (v).

$$\frac{S_D}{N_D} = \frac{S_D}{Q_D} \cdot \frac{Q_D}{N_D}$$
$$t_D = v.u_D$$
$$u_D = \frac{1}{v}(t_D)$$
$$u_D = \frac{1}{v}\left(\tau_T\,PAF_\tau\left(\frac{1}{p}\right)\right) \qquad (22)$$

Applying the intervention effect size to the overall suicide rate in the population

Equation (23) demonstrates the application of an intervention effect size to the incidence of suicide attempts in the population.

$$u_T^{Int} = RR_{Int}\, u_T^{Com} \tag{23}$$

Where: u_T^{Int} is the incidence of suicide attempts in the population in the intervention scenario – i.e., post-intervention incidence; RR_{Int} is the intervention effect size expressed as a relative risk; and u_T^{Com} is the incidence of suicide attempts in the population in the comparator scenario – i.e., pre-intervention incidence.

Equation (24) presents the post-intervention overall suicide rate in the population (τ_T^{Int}) as a function of the pre-intervention overall suicide rate in the population (τ_T^{Com}).

$$\begin{aligned}
\tau_T^{Int} &= v.\, u_T^{Int} \\
&= v\left(RR_{Int}\, u_T^{Com}\right) \\
&= v\,(RR_{Int})\left(\frac{1}{v}\right)\left(\tau_T^{Com}\right) \\
\tau_T^{Int} &= RR_{Int}\, \tau_T^{Com}
\end{aligned} \tag{24}$$

Where: τ_T^{Int} is the overall suicide rate in the population in the intervention scenario – i.e., the post-intervention suicide rate; and τ_T^{Com} is the overall suicide rate in the population in the comparator scenario – i.e., the pre-intervention suicide rate.

Applying the intervention effect size to the suicide probability among people with depression/anxiety

Equation (25) demonstrates the application of an intervention effect size to the incidence of suicide attempts among people with depression/anxiety.

$$u_D^{Int} = RR_{Int}\, u_D^{Com} \tag{25}$$

Where: u_D^{Int} is the incidence of suicide attempts among people with depression/anxiety in the intervention scenario – i.e., post-intervention incidence; RR_{Int} is the intervention effect size expressed as a relative risk; and u_D^{Com} is the incidence of suicide attempts among people with depression/anxiety in the comparator scenario – i.e., pre-intervention incidence.

Equation (26) presents the post-intervention suicide probability among people with depression/anxiety as a function of the pre-intervention overall suicide rate in the population.

$$\begin{aligned}
t_D^{Int} &= v.\, u_D^{Int} \\
&= v\left(RR_{Int}\, u_D^{Com}\right) \\
&= v\,(RR_{Int})\left(\frac{1}{v}\right)\left(t_D^{Com}\right) \\
&= RR_{Int}\, t_D^{Com} \\
t_D^{Int} &= RR_{Int}\left[\tau_T^{Com}\, PAF_\tau\left(\frac{1}{p}\right)\right]
\end{aligned} \tag{26}$$

Where: t_A^{Int} is the suicide probability among people with depression/anxiety in the intervention scenario – i.e., the post-intervention suicide probability; and t_A^{Com} is the suicide probability among people with depression/anxiety in the comparator scenario – i.e., the pre-intervention suicide probability.

Applying the intervention effect size to suicide probability among people at risk of depression/anxiety

Equation (27) demonstrates the application of an intervention effect size to the incidence of suicide attempts among people at risk of depression/anxiety.

$$u_A^{Int} = RR_{Int}\, u_A^{Com} \tag{27}$$

Where: u_A^{Int} is the incidence of suicide attempts among people at risk of depression/anxiety in the intervention scenario – i.e., post-intervention incidence; RR_{Int} is the intervention effect size expressed as a relative risk; and u_A^{Com} is the incidence of suicide attempts among people at risk of depression/anxiety in the comparator scenario – i.e., pre-intervention incidence.

Equation (28) presents the post-intervention suicide probability among people at risk of depression/anxiety as a function of the pre-intervention overall suicide rate in the population.

$$\begin{aligned} t_A^{Int} &= v.\, u_A^{Int} \\ &= v\left(RR_{Int}\, u_A^{Com}\right) \\ &= v\,(RR_{Int})\left(\frac{1}{v}\right)\left(t_A^{Com}\right) \\ &= RR_{Int}\, t_A^{Com} \\ t_A^{Int} &= RR_{Int}\left[\tau_T^{Com}\,(1 - PAF_\tau)\left(\frac{1}{1-p}\right)\right] \end{aligned} \tag{28}$$

Where: t_A^{Int} is the suicide probability among people at risk of depression/anxiety in the intervention scenario – i.e., the post-intervention suicide probability; and t_A^{Com} is the suicide probability among people at risk of depression/anxiety in the comparator scenario – i.e., the pre-intervention suicide probability.

Appendix S6 – Methods used to account for dependent comorbidity between depression and anxiety

It is common for comorbidity to be observed between two or more health conditions, particularly among mental health conditions. For example, depression and anxiety are highly comorbid with around 41.6% (SE: 0.9%) of respondents that have a 12-month diagnosis of major depression in the World Mental Health Survey also being diagnosed with a 12-month anxiety disorder.[22] Appendix Figure 4 depicts the overlap between the prevalence risk (i.e., probability) between two health conditions.

Appendix Figure 4 Comorbidity between two overlapping health conditions

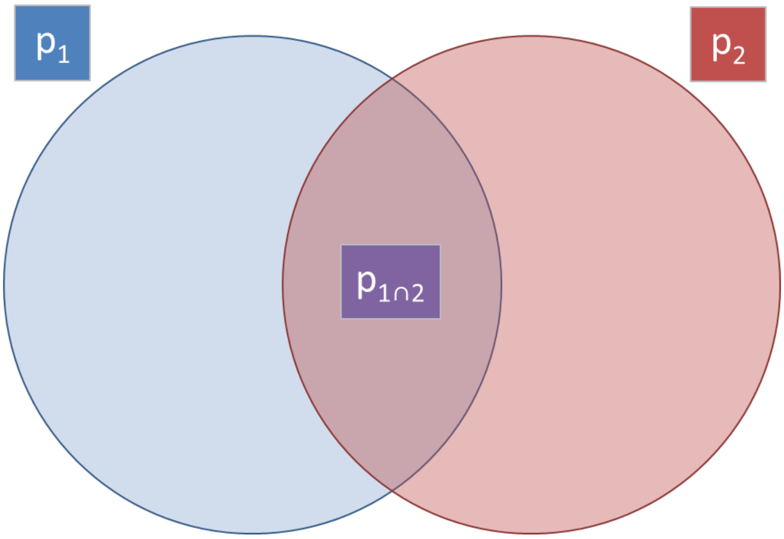

Where: p_1 is the prevalence of health condition 1; p_2 is the prevalence of health condition 2; and $p_{1\cap 2}$ is the intersection of prevalence between the two health conditions – i.e., comorbid prevalent cases that simultaneously experience conditions 1 and 2.

The Global Burden of Disease study conventionally assumes independent comorbidity between pairs of comorbid health conditions, where the probability of having two health conditions is the product of the probability of each.[23] Equation (29) provides a mathematical representation of independent comorbidity between two health conditions:

$$\begin{aligned} p_{1\cup 2} &= p_1 + p_2 - p_{1\cap 2} \\ &= p_1 + p_2 - p_1\, p_2 \\ p_{1\cup 2} &= 1 - (1 - p_1)(1 - p_2) \end{aligned} \tag{29}$$

Where: $p_{1\cup 2}$ is the prevalence of health condition 1 or health condition 2; p_1 is the prevalence of health condition 1, inclusive of comorbid cases; p_2 is the prevalence of health condition 2, inclusive of comorbid cases; and $p_{1\cap 2}$ is the prevalence of comorbid cases of health condition 1 and health condition 2.

In practice, it is unlikely for independent comorbidity to be observed between two health conditions. Mathers et al.[23] describe a method to account for dependent comorbidity – i.e., the situation where the probability of having a pair of health conditions is greater than the product of the probabilities for each health condition. Equation (30) provides a mathematical representation of dependent comorbidity between two health conditions:

$$p_{1\cup 2} = p_1 + p_2 - f_{1\cap 2}\, p_1\, p_2 \tag{30}$$

Where: $f_{1\cap 2}$ is the comorbidity factor, as described in Equation (31).

$$f_{1\cap 2} = \frac{p_{1\cap 2}}{p_1\, p_2} \tag{31}$$

A comorbidity factor ($f_{1\cap 2}$) of 3 subsequently indicates that the combined prevalence of health conditions 1 and 2 is three times more likely than would be expected if the occurrence of the two conditions was independent. A comorbidity factor of 1 would indicate that the comorbidity between health conditions 1 and 2 is independent.

Data from the World Mental Health Surveys[21,22] (see Appendix Table 4) were used to determine the dependent comorbidity factor ($f_{1\cap 2}$) for depression and anxiety, which was estimated to be 4.25 (SE: 0.13).

Appendix Table 4 Data used to estimate the comorbidity factor between depression and anxiety

Model input parameter	Value and uncertainty range	Source
Prevalence of depression (p_1)	4.7% (SE: 0.1)	Kessler et al.[22]
Prevalence of anxiety (p_2)	9.8% (SE: 0.2)	Alonso et al.[21]
Prevalence of comorbid depression and anxiety ($p_{1\cap 2}$)	2.0% (SE: 0.04)	Kessler et al.[22]
Dependent comorbidity factor between depression and anxiety ($f_{1\cap 2}$)	4.25 (SE: 0.13)	Derived estimate

Abbreviations: *SE - standard error.*

The dependent comorbidity methods described above are directly applicable to epidemiological parameters for depression and anxiety (i.e., prevalence) that are expressed as probabilities bounded between 0 and 1. For example, if the prevalence of depression was 4.0% and the prevalence of anxiety was 8.0%, then the combined prevalence of depression/anxiety would be 10.6%.

Conversely, several epidemiological parameters related to depression and anxiety (i.e., incidence and remission) are expressed as instantaneous rates (a.k.a. hazards), which denote the number of events per unit of time – e.g., the number of incident cases of depression per person year. Unlike rates, probabilities denote the likelihood of an event occurring over a given period of time. Incidence rates and remission rates for depression and anxiety were thus converted into 1-year probabilities using Equation (32), prior to the application of the dependent comorbidity method to estimate the 1-year incidence probability and remission probability for depression/anxiety. These 1-year probabilities were then, in turn, converted to the incidence rate and remission rate for depression/anxiety using Equation (33).

$$p = 1 - e^{(-rt)} \tag{32}$$

$$r = \frac{-ln(1-p)}{t} \tag{33}$$

Where: p is the probability; r is the rate; and t is the time period over which the probability applies.

Appendix S7 – Additional details on the costing analysis

Overview

The costing framework and methods developed by the WHO-CHOICE program were used to estimate the country-specific costs of universal and indicated SEL programs to prevent depression/anxiety and suicide among adolescents aged 12-17 years.[2] WHO-CHOICE adopts an ingredients approach that multiplies quantities of resources required to implement an intervention by the respective price or unit cost of those resources. Resource needs are split between program-level costs (such as program management, training, media and regulation) and patient-level costs incurred at the level of the health care facility. Country-specific intervention costs were estimated using previous NCD costing templates developed and used by WHO for evaluating NCD prevention and control; both in the context of identifying 'best buys' and for subsequent work on global 'price tags', NCD investment cases and updates to Appendix 3 of the WHO NCD Global Action Plan.[97,98]

Previous NCD costing templates were modified to account for the different stages involved with implementing universal and indicated SEL programs. A resource needs matrix was used to identify resource needs for universal/indicated SEL programs, consisting of four stages of policy development (planning stage [year 1]; policy development [year 2]; partial implementation [years 3-5]; full implementation [year 6 onwards], and six categories of resource use: human resources; training; meetings; mass media; supplies and equipment; and other resources. The cost of training and meetings was based on the frequency of meetings and workshops within a year, their average duration, the number of national and sub-national participants (plus associated support staff), and the size of the meeting venue.

To derive comparable estimates of resource needs across interventions and countries, resource need estimates were made for the different resource categories for a standardized country of 50 million people (split into 10 provinces of 5 million and 10 districts of 0·5 million persons). These estimates were subsequently adjusted to reflect the actual population size and administrative composition of each country. Unit costs for resource items were taken from the WHO-CHOICE database (https://www.who.int/teams/health-systems-governance-and-financing/economic-analysis), which contains country-specific estimates for primary care visits of different durations, salaries, per diem allowances (for training and meetings), media costs and consumable items, including fuel and office supplies. Generation of these estimates was based on an econometric analysis of a multinational dataset, using gross national income per capita (plus other explanatory variables) to predict unit costs in different WHO Member States.[99]

Country-specific costs available through the WHO-CHOICE database were converted to 2017 international dollars (2017 I$) using USD consumer price inflation indices for traded goods (e.g., drugs and consumables) and country-specific GDP price deflators for non-traded goods (e.g., staff wages, inpatient days and outpatient visits).[100] All costs were discounted at a 3% annual rate and modelled with ±20% uncertainty ranges using the PERT distribution.

Data required to cost program delivery

Data were obtained from studies included in the prior meta-analysis of intervention effect sizes to estimate resource use associated with program delivery. Data from 14 studies suggested that a total of 20 hours (range: 2 to 40) would be required to train program facilitators to deliver SEL programs in the classroom (see Appendix Table 5). The universal SEL program comprised 15 hours (range: 6 to 36) of total contact time with students, based on data from 27 studies (see Appendix Table 6). While the indicated program involved 10 hours (range: 3.5 to 18) of total contact time, based on data from 27 studies (see Appendix Table 7). The indicated SEL program additionally involved time spent by facilitators to screen students for subthreshold depression/anxiety using locally-validated mental health questionnaires. Facilitators were assumed to spend 30 minutes per class disseminating mental health questionnaires and 5 minutes per student scoring each questionnaire.

Appendix Table 5 Study data used to estimate the average time required to train facilitators to deliver socio-emotional learning (SEL) programs in the classroom

Study author and year	Total training time (hours)	Reference
Bond et al., (2004); Patton et al., (2006)	40.0	[24,25]
Buttigieg et al., (2015)	2.0	[26]
Kindt et al., (2014)	32.0	[30]
Kraag et al., (2009)	6.0	[31]
Melnyk et al., (2013, 2015)	8.0	[34,35]
Merry et al., (2004)	20.0	[37]
Park et al., (2000); Mason et al., (2007)	24.0	[40,41]
Quayle et al., (2001)	30.0	[44]
Rivet-Duval et al., (2011)	16.0	[45]
Roberts et al., (2009)	16.0	[46]
Sawyer et al., (2010a, b)	8.0	[49,50]
Stallard et al., (2013); Stallard et al., (2012)	12.0	[54,55]
Stallard et al., (2014, 2015)	16.0	[56,57]
Tak et al., (2014, 2016)	40.0	[58,59]

Appendix Table 6 Study data used to estimate the average total contact time involved in delivering the universal socio-emotional learning (SEL) program

Study author and year	Total contact time (hours)	Reference
Bond et al., (2004); Patton et al., (2006)	15.0	[24,25]
Buttigieg et al., (2015)	8.5	[26]
Butzer et al., (2017)	24.0	[27]
Horowitz et al., (2007)	12.0	[28]
Khalsa et al., (2012)	27.0	[29]
Kindt et al., (2014)	13.5	[30]
Kraag et al., (2009)	13.0	[31]
Langer et al., (2017)	6.0	[32]
Leventhal et al., (2015)	23.0	[33]
Melnyk et al., (2013, 2015)	15.0	[34,35]
Mendelson et al., (2010)	36.0	[36]
Merry et al., (2004)	11.0	[37]
Nash (2007)	7.0	[38]
Noggle et al., (2012)	14.0	[39]
Park et al., (2000); Mason et al., (2007)	10.0	[40,41]
Pössel et al., (2004)	15.0	[42]
Pössel et al., (2013)	15.0	[43]
Quayle et al., (2001)	11.0	[44]
Rivet-Duval et al., (2011)	11.0	[45]
Roberts et al., (2009)	20.0	[46]
Rose et al., (2014)	16.5	[47]
Ruini et al., (2007)	8.0	[48]
Sawyer et al., (2010a, b)	22.5	[49,50]
Sibinga et al., (2013)	10.0	[52]
Stallard et al., (2013); Stallard et al., (2012)	11.0	[54,55]
Stallard et al., (2014, 2015)	9.0	[56,57]
Tak et al., (2014, 2016)	15.5	[58,59]

Appendix Table 7 Study data used to estimate the average total contact time involved in delivering the indicated socio-emotional learning (SEL) program

Study author and year	Total contact time (hours)	Reference
Balle & Tortella-Feliu (2010)	4.5	[62]
Bella-Awusah et al., (2016)	4.5	[63]
Berry & Hunt (2009)	8.0	[64]
Dobson et al., (2010)	11.5	[65]
Gaete et al., (2016)	8.5	[66]
Gau et al., (2012)	6.0	[67]
Hunt et al., (2009)	11.5	[68]
Jacob & De Guzman (2016)	12.0	[69]
Kwok et al., (2016)	12.0	[70]
Livheim et al., (2015)	12.0	[71]
Martinsen et al., (2019)	17.5	[72]
Noel et al., (2013)	18.0	[74]
Poppelaars et al., (2016)	8.0	[75]
Puskar et al., (2003)	7.5	[76]
Pybis et al., (2015)	7.5	[77]
Roberts et al., (2003); Roberts et al., (2004)	18.0	[78,79]
Rohde et al., (2014a); Rohde et al., (2015)	6.0	[80,81]
Sheffield et al., (2006)	12.0	[83]
Smith et al., (2015)	5.0	[84]
Spence et al., (2003)	7.0	[85]
Stallard et al., (2013)	8.5	[55]
Stasiak et al., (2014)	3.5	[86]
Wijnhoven et al., (2014)	7.0	[87]
Woods et al., (2011)	12.0	[88]
Young et al., (2006); Young et al., (2009)	13.5	[89,90]
Young et al., (2010)	13.5	[91]
Young et al., (2016); Young et al., (2019)	11.5	[92,93]

Appendix S8 – Additional results for the base case analysis

Absolute results (per 1 population) are presented for the universal and indicated school-based SEL programs in Appendix Table 8, while the corresponding population-standardised results (per 1,000,000 population) are presented in Appendix Table 9.

Appendix Table 8 **Absolute results for the base case analysis (per 1 population)**

**Intervention type & country income group**	**CE ratio (I$ per HLYG) [a]** *(95% UI)*	**Intervention costs (2017 I$)** *(95% UI)*	**Healthy life years gained [a]** *(95% UI)*	**YLDs averted** *(95% UI)*	**YLLs averted** *(95% UI)*	**Prevalent cases of depression/anxiety averted** *(95% UI)*	**Suicide deaths averted** *(95% UI)*
Universal SEL program							
LLMICs (n = 10)	$958 (724 to 1,337)	$240.82M (222.11M to 259.44M)	251,290 (181,685 to 324,820)	205,307 (139,087 to 271,938)	45,983 (17,659 to 72,438)	1,007,374 (695,841 to 1,328,851)	879 (370 to 1,375)
UMHICs (n = 10)	$2,006 (1,551 to 2,782)	$408.12M (374.82M to 440.52M)	203,443 (146,383 to 258,872)	176,127 (119,377 to 227,786)	27,316 (16,880 to 38,514)	861,624 (594,481 to 1,125,850)	585 (347 to 832)
Indicated SEL program							
LLMICs (n = 10)	$11,123 (6,003 to 22,290)	$150.21M (138.83M to 161.75M)	13,505 (6,681 to 24,132)	12,117 (5,614 to 22,123)	1,388 (384 to 3,295)	59,827 (27,059 to 109,718)	26 (8 to 64)
UMHICs (n = 10)	$18,473 (10,546 to 37,703)	$212.06M (195.72M to 227.19M)	11,480 (5,442 to 20,153)	10,703 (4,672 to 19,342)	776 (329 to 1,483)	52,647 (23,699 to 98,343)	16 (6 to 33)

Abbreviations: 95% UI: 95% uncertainty interval; CE - cost-effectiveness; HLYG - healthy life year gained; I$ - international dollars; LLMICs - low- to lower middle-income countries; M - millions; SEL - socio-emotional learning; UMHICs - upper middle- to high-income countries; YLDs - years lived with disability; YLLs - years of life lost.

[a] *Healthy Life Years Gained (HLYGs) are equivalent to Disability-Adjusted Life Years (DALYs) averted – i.e., the sum of YLLs averted and YLDs averted.*

Appendix Table 9 Population-standardised results for the base case analysis (per 1,000,000 population)

**Intervention type & country income group**	**CE ratio (I$ per HLYG)** [a] *(95% UI)*	**Intervention costs (2017 I$)** *(95% UI)*	**Healthy life years gained** [a] *(95% UI)*	**YLDs averted** *(95% UI)*	**YLLs averted** *(95% UI)*	**Prevalent cases of depression/anxiety averted** *(95% UI)*	**Suicide deaths averted** *(95% UI)*
Universal SEL program							
LLMICs (n = 10)	$958 (724 to 1,337)	$95,548 (88,127 to 102,939)	99.70 (72.09 to 128.88)	81.46 (55.19 to 107.90)	18.24 (7.01 to 28.74)	399.7 (276.1 to 527.2)	0.3 (0.1 to 0.5)
UMHICs (n = 10)	$2,006 (1,551 to 2,782)	$162,213 (148,977 to 175,088)	80.86 (58.18 to 102.89)	70.00 (47.45 to 90.54)	10.86 (6.71 to 15.31)	342.5 (236.3 to 447.5)	0.2 (0.1 to 0.3)
Indicated SEL program							
LLMICs (n = 10)	$11,123 (6,003 to 22,290)	$59,600 (55,082 to 64,179)	5.36 (2.65 to 9.57)	4.81 (2.23 to 8.78)	0.55 (0.15 to 1.31)	23.7 (10.7 to 43.5)	0.010 (0.003 to 0.025)
UMHICs (n = 10)	$18,473 (10,546 to 37,703)	$84,284 (77,792 to 90,298)	4.56 (2.16 to 8.01)	4.25 (1.86 to 7.69)	0.31 (0.13 to 0.59)	20.9 (9.4 to 39.1)	0.006 (0.002 to 0.013)

Abbreviations: 95% UI: 95% uncertainty interval; CE - cost-effectiveness; HLYG - healthy life year gained; I$ - international dollars; LLMICs - low- to lower middle-income countries; SEL - socio-emotional learning; UMHICs - upper middle- to high-income countries; YLDs - years lived with disability; YLLs - years of life lost.

[a] *Healthy Life Years Gained (HLYGs) are equivalent to Disability-Adjusted Life Years (DALYs) averted – i.e., the sum of YLLs averted and YLDs averted.*

[b] *Per 1,000,000 population.*

Appendix S9 – Univariate deterministic sensitivity analysis results

Appendix Figure 5 presents the tornado graphs for the univariate deterministic sensitivity analysis of the universal school-based SEL program, among low- to lower middle-income countries (LLMICs) and upper middle- to high-income countries (UMHICs). Each tornado graph outlines the top ten input parameters (out of a total 423) that had the largest impact on the cost-effectiveness ratio following a ±10% change in the mean parameter value.

For LLMICs, the input parameters that led to the largest percentage change to the cost-effectiveness ratio comprised: the intervention effect size applied to depression/anxiety cases (maximal change of 62.0%); the intervention effect size applied to suicide deaths (15.8%); and the disability weight for depression/anxiety (7.8%). For UMHICs, the input parameters that led to the largest percentage change included: the intervention effect size applied to depression/anxiety cases (97.9%); the disability weight for depression/anxiety (8.6%); and the remission rate for depression (4.0%). The remaining input parameters across both LLMICs and UMHICs involved maximal changes to the cost-effectiveness ratio that were less than 3.0%.

Appendix Figure 5 Tornado graphs for the univariate deterministic sensitivity analysis involving the universal school-based SEL program, presented by country income group

a) LLMICs

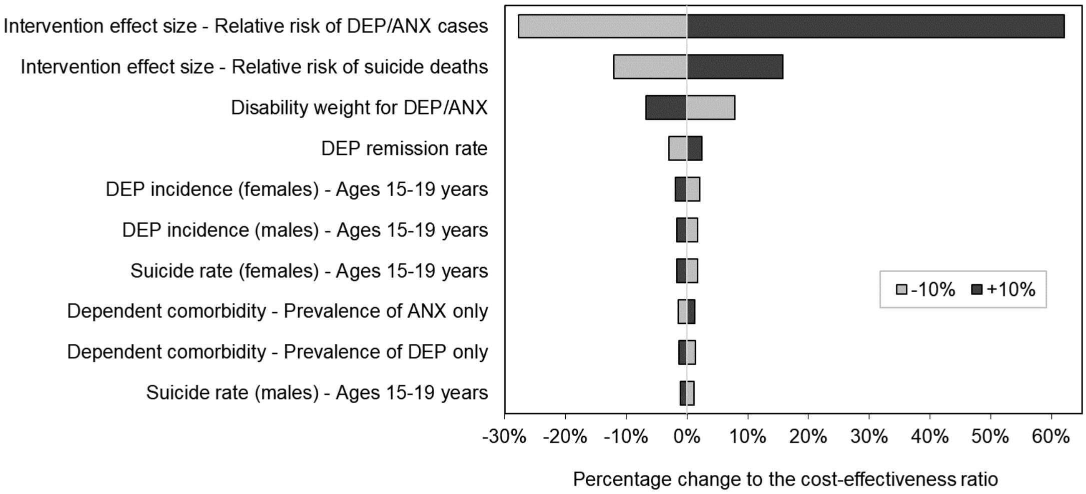

b) UMHICs

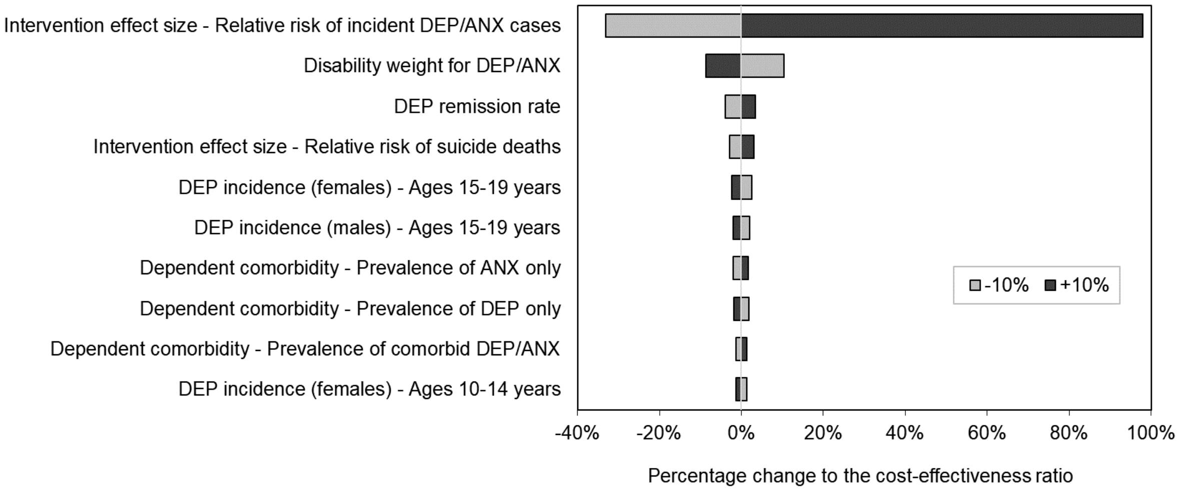

Abbreviations: ANX - anxiety; DEP - depression; LLMICs - low- to lower middle-income countries; UMHICs - upper middle- to high-income countries.

Appendix Figure 6 presents the tornado graphs for the univariate deterministic sensitivity analysis of the indicated school-based SEL program, among LLMICs and UMHICs. Each tornado graph outlines the top ten input parameters (out of a total 425) that had the largest impact on the cost-effectiveness ratio following a ±10% change in the mean parameter value.

For LLMICs, the input parameters that led to the largest percentage change to the cost-effectiveness ratio comprised: the intervention effect size applied to depression/anxiety cases (maximal change of 29.8%); the increased risk of developing depression/anxiety among individuals with subthreshold depression (23.6%); the prevalence of subthreshold depression/anxiety (10.7%); the disability weight for depression/anxiety (9.2%); and the intervention effect size applied to suicide deaths (8.6%). For UMHICs, the input parameters that led to the largest percentage change included: the intervention effect size applied to depression/anxiety cases (35.9%); the increased risk of developing depression/anxiety among people with subthreshold depression (28.2%); the disability weight for depression/anxiety (10.7%); the prevalence of subthreshold depression/anxiety (10.7%); and the remission rate for depression (4.1%). The remaining input parameters across both LLMICs and UMHICs involved maximal changes to the cost-effectiveness ratio that were less than 3.5%.

Appendix Figure 6 Tornado graphs for the univariate deterministic sensitivity analysis involving the indicated school-based SEL program, presented by country income group

a) LLMICs

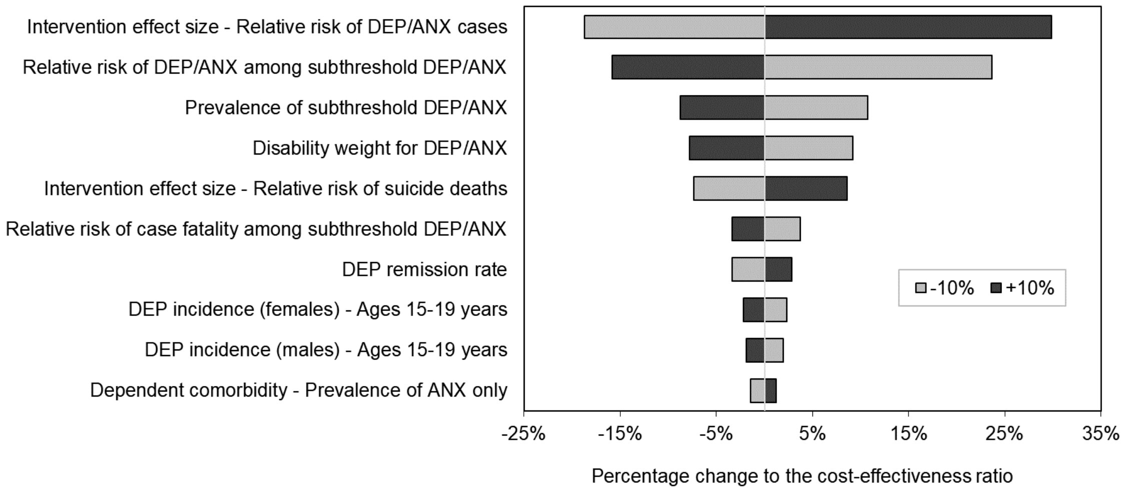

b) UMHICs

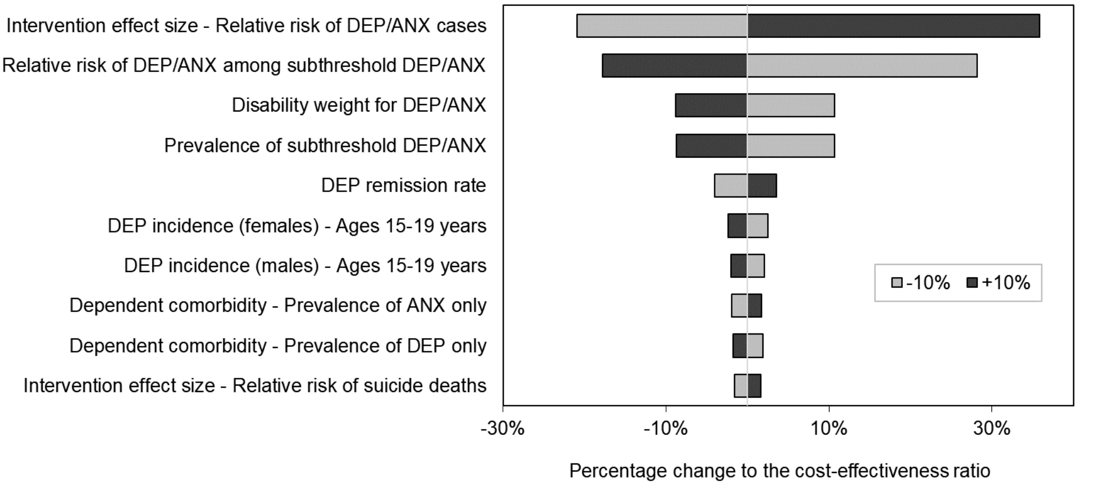

Abbreviations: ANX - anxiety; DEP - depression; LLMICs - low- to lower middle-income countries; UMHICs - upper middle- to high-income countries.

Appendix S10 – Multivariate probabilistic sensitivity analysis results

Appendix Figure 7 presents the tornado graphs for the multivariate probabilistic sensitivity analysis of the universal school-based SEL program, among LLMICs and UMHICs. Each tornado graph outlines the top ten input parameters (out of a total 423) that had the largest impact on the cost-effectiveness ratio. The strength of association between each input parameter and the final outcome was measured using Spearman's ranked correlation coefficient (r_s), where absolute values of: 0.00-0.19 denote a very weak correlation; 0.20-0.39 denote a weak correlation; 0.40-0.59 denote a moderate correlation; 0.60-0.79 denote a strong correlation; and 0.80-1.00 denote a very strong correlation.[44]

For LLMICs: the intervention effect size applied to depression/anxiety cases was strongly correlated with the cost-effectiveness ratio ($|r_s|=0.80$); the intervention effect size applied to suicide deaths was moderately correlated ($|r_s|=0.46$); and the disability weight for depression/anxiety was weakly correlated ($|r_s|=0.23$). For UMHICs: the intervention effect size applied to depression/anxiety cases was strongly correlated with the cost-effectiveness ratio ($|r_s|=0.92$); and the disability weight for depression/anxiety was weakly correlated ($|r_s|=0.31$). Very weak correlations ($|r_s|<0.20$) were observed for the remaining input parameters across both LLMICs and UMHICs.

Appendix Figure 7 Tornado graphs for the multivariate probabilistic sensitivity analyses involving the universal school-based SEL program, presented by country income group

a) LLMICs

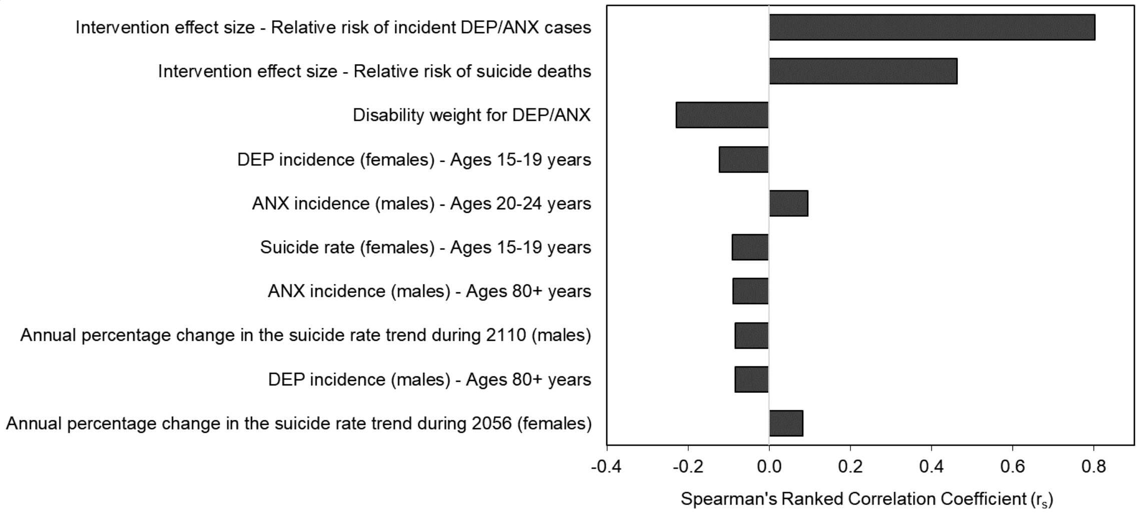

b) UMHICs

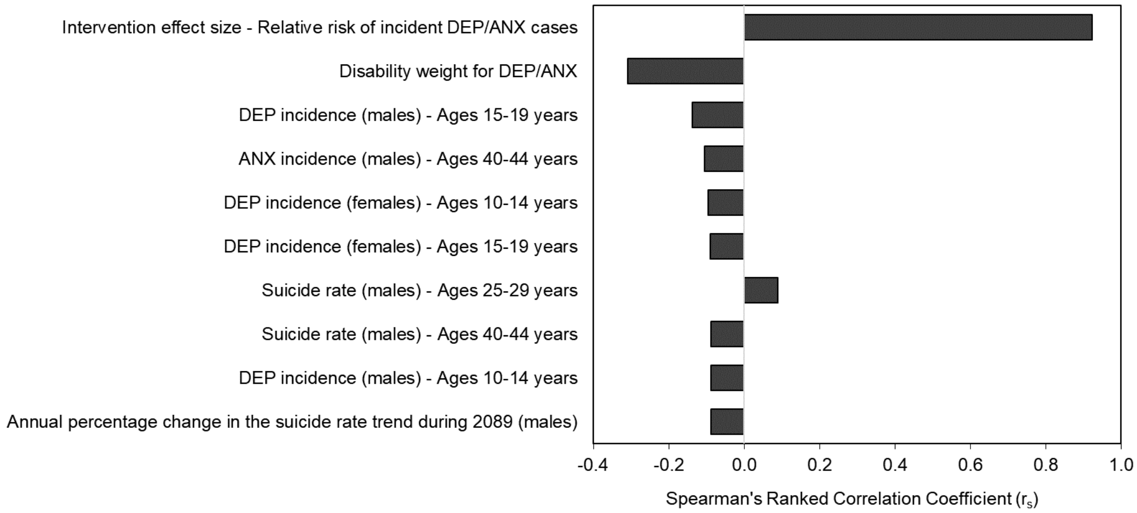

Abbreviations: ANX - anxiety; DEP - depression; LLMICs - low- to lower middle-income countries; UMHICs - upper middle- to high-income countries.

Appendix Figure 8 presents the tornado graphs for the multivariate probabilistic sensitivity analysis of the indicated school-based SEL program, among LLMICs and UMHICs. Each tornado graph outlines the top ten input parameters (out of a total 425) that had the largest impact on the cost-effectiveness ratio.

For LLMICs: the increased risk of developing depression/anxiety among individuals with subthreshold depression was strongly correlated with the cost-effectiveness ratio ($|r_s|=0.74$); the prevalence of subthreshold depression/anxiety was moderately correlated ($|r_s|=0.42$); and the intervention effect size applied to depression/anxiety cases was moderately correlated ($|r_s|=0.41$). For UMHICs: the increased risk of developing depression/anxiety among individuals with subthreshold depression was strongly correlated with the cost-effectiveness ratio ($|r_s|=0.73$); the intervention effect size applied to depression/anxiety cases was weakly correlated ($|r_s|=0.38$); and the prevalence of subthreshold depression/anxiety was weakly correlated ($|r_s|=0.38$). Very weak correlations ($|r_s|<0.20$) were observed for the remaining input parameters across both LLMICs and UMHICs.

Appendix Figure 8 Tornado graphs for the multivariate probabilistic sensitivity analyses involving the indicated school-based SEL program, presented by country income group

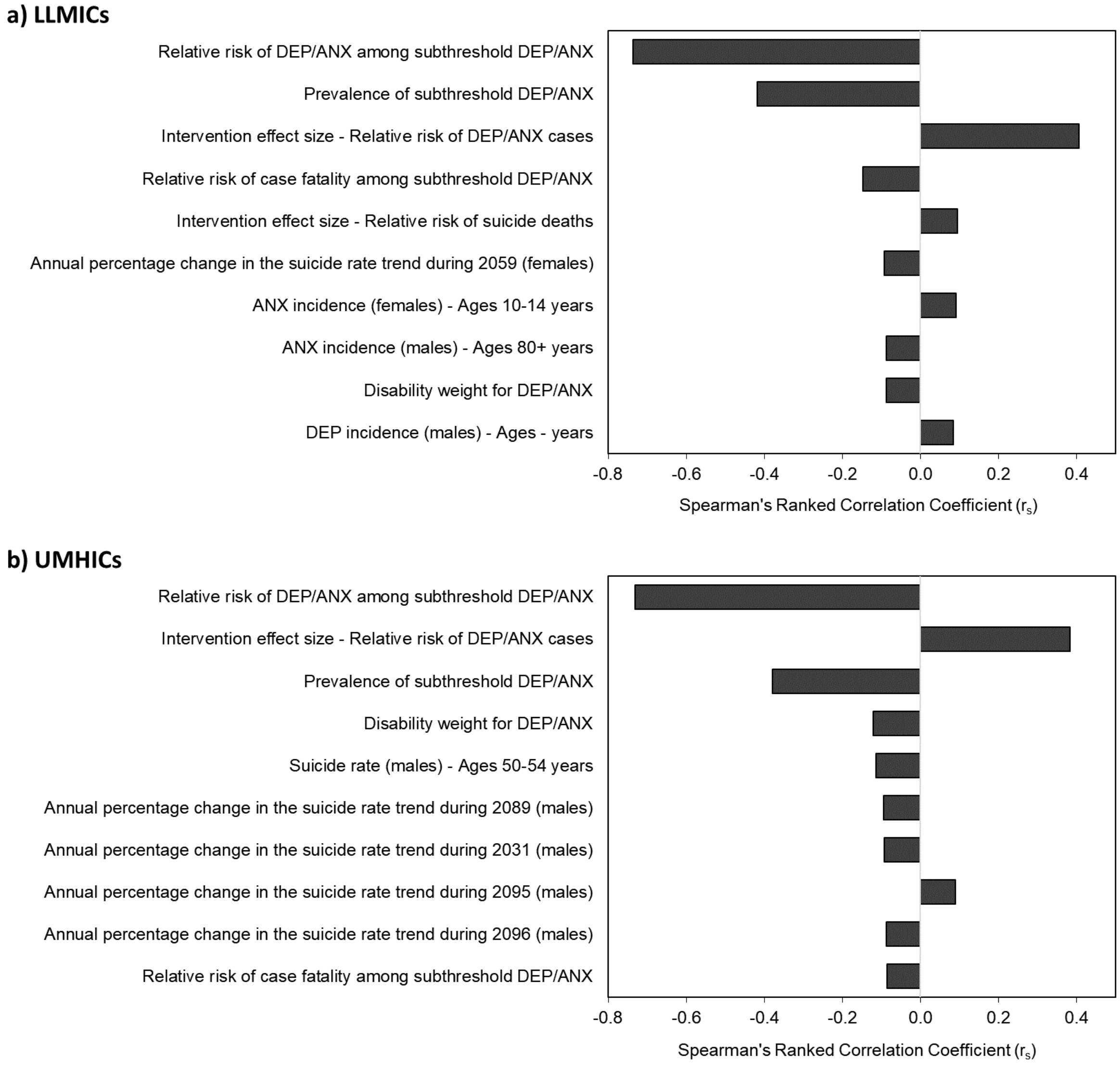

Abbreviations: ANX - anxiety; DEP - depression; LLMICs - low- to lower middle-income countries; UMHICs - upper middle- to high-income countries.

Appendix S11 – Threshold analysis results

A threshold analysis was conducted to examine the impact of simultaneously reducing the intervention effect sizes applied to depression/anxiety and suicides from 0% to 100% (where a 100% reduction leads to a RR of 1.00). Threshold analysis results for the universal SEL program are presented for: low- to lower middle-income countries (LLMICs) in Appendix Figure 9; and upper middle- to high-income countries (UMHICs) in Appendix Figure 10. The cost-effectiveness ratios remained below I$5,000 per HLYG following an 80% reduction in the intervention effect size for LLMICs and a 59% reduction for UMHICs. Threshold analysis results for the indicated SEL program are presented for: LLMICs in Appendix Figure 11; and UMHICs in Appendix Figure 12. The cost-effectiveness ratio remained below I$50,000 per HLYG following a 77% reduction in the intervention effect size for LLMICs and a 63% reduction for UMHICs.

Appendix Figure 9 Threshold analysis results for the universal SEL program in LLMICs

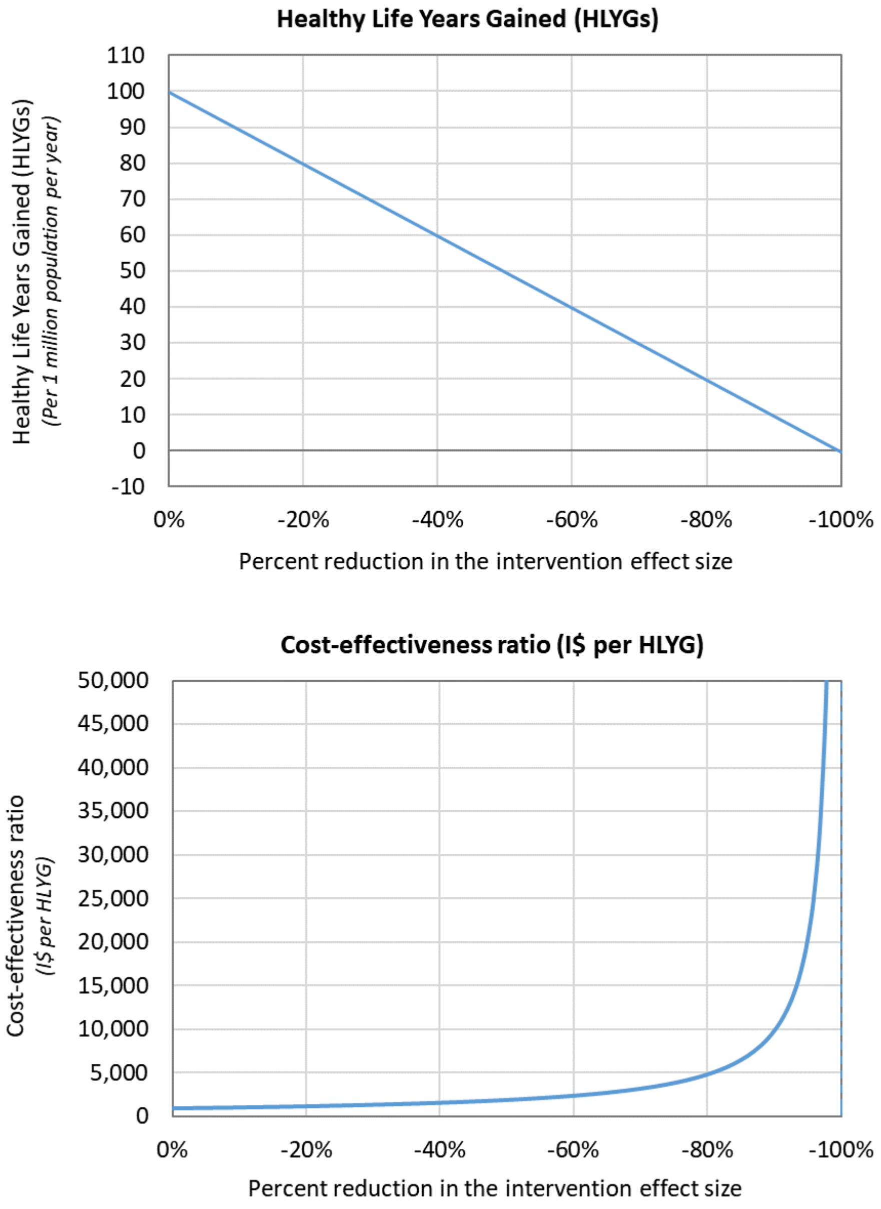

Abbreviations: HLYG - healthy life year gained; I$ - international dollars; LLMICs - low- to lower middle-income countries; SEL - socio-emotional learning.

Note: Percent reductions in the intervention effect size were applied equally to both the intervention effect size applied to depression/anxiety cases and the intervention effect size applied to suicide deaths.

Appendix Figure 10 Threshold analysis results for the universal SEL program in UMHICs

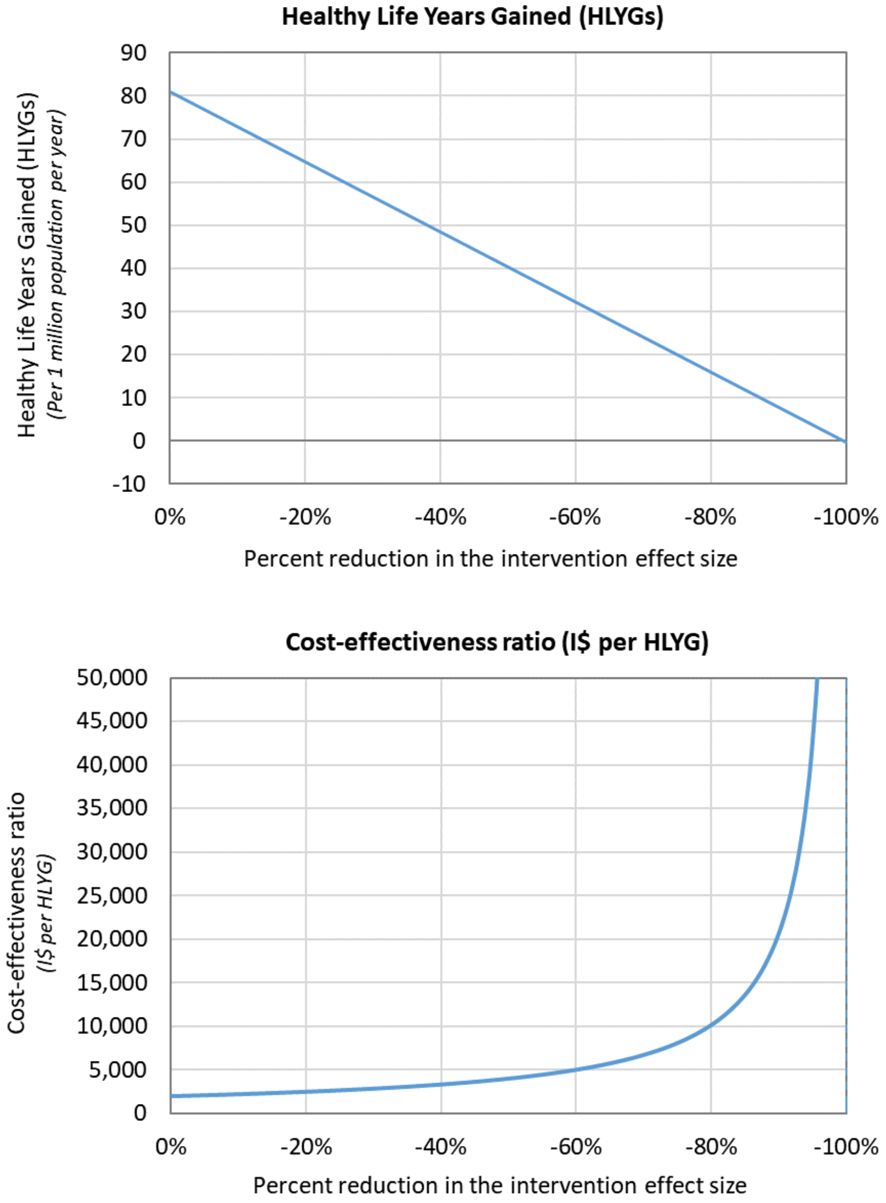

Abbreviations: HLYG - healthy life year gained; I$ - international dollars; UMHICs - upper middle- to high-income countries; SEL - socio-emotional learning.

Note: Percent reductions in the intervention effect size were applied equally to both the intervention effect size applied to depression/anxiety cases and the intervention effect size applied to suicide deaths.

Appendix Figure 11 Threshold analysis results for the indicated SEL program in LLMICs

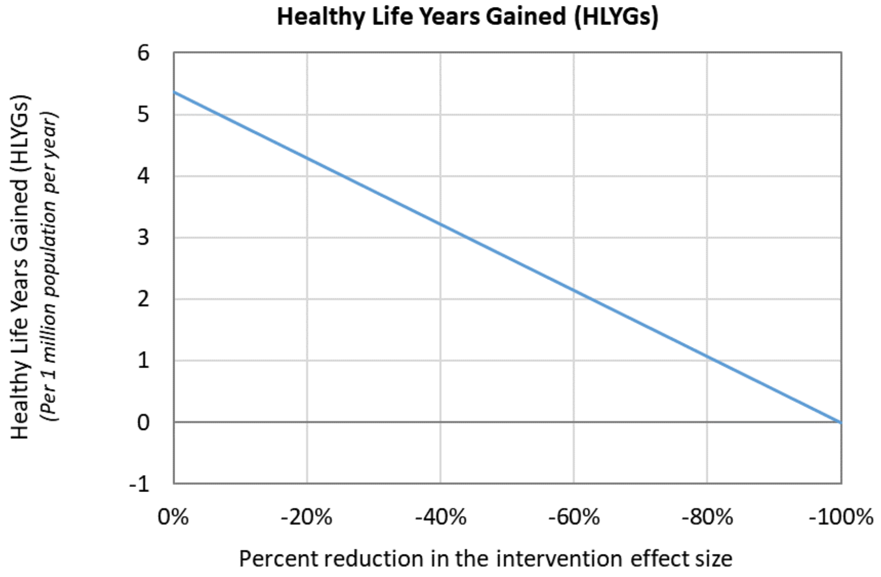

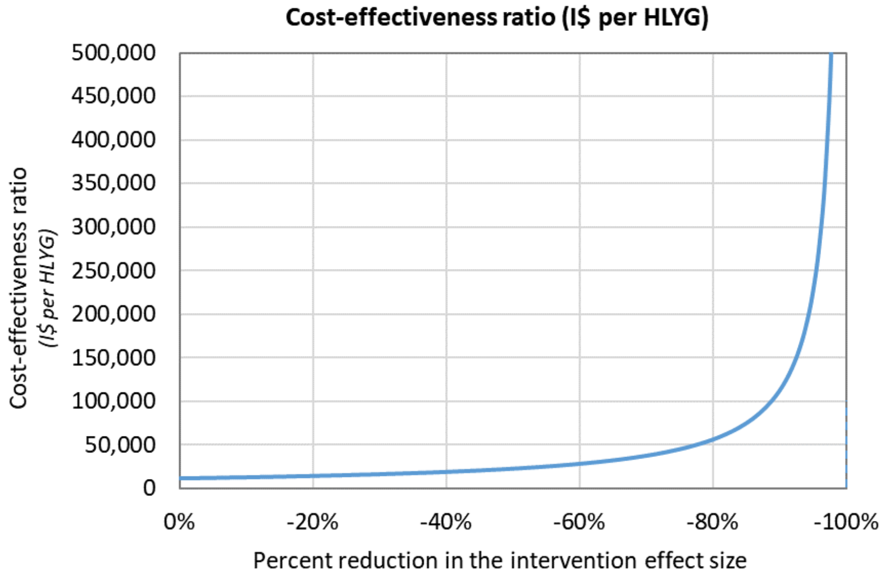

Abbreviations: HLYG - healthy life year gained; I$ - international dollars; LLMICs - low- to lower middle-income countries; SEL - socio-emotional learning.

Note: Percent reductions in the intervention effect size were applied equally to both the intervention effect size applied to depression/anxiety cases and the intervention effect size applied to suicide deaths.

Appendix Figure 12 Threshold analysis results for the indicated SEL program in UMHICs

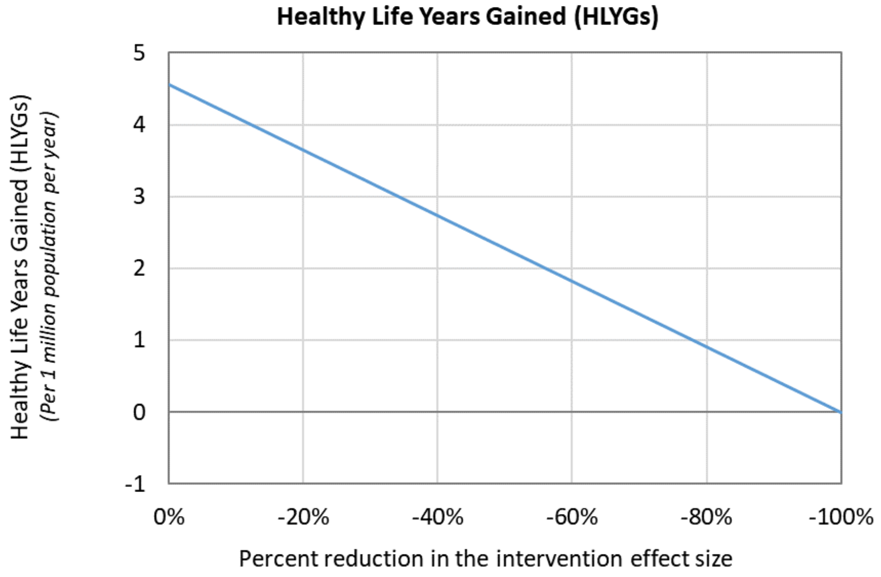

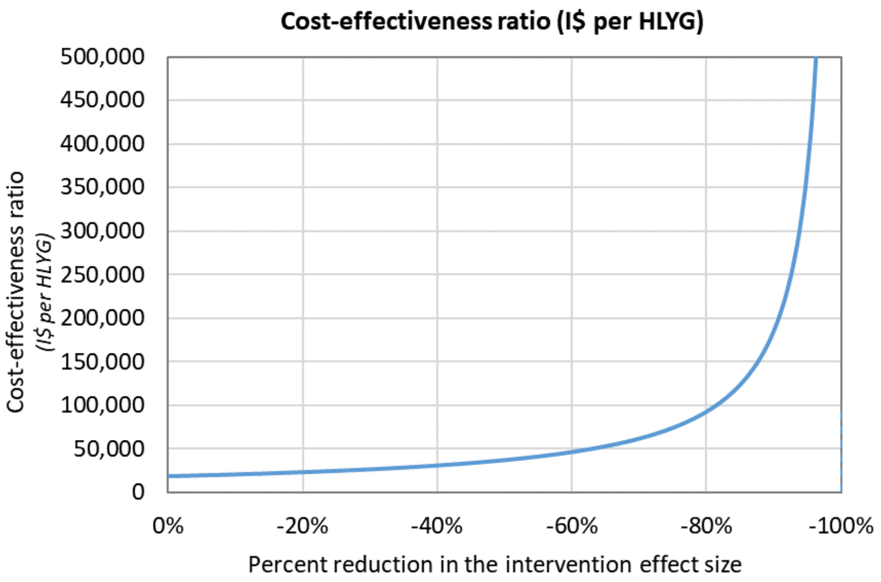

Abbreviations: HLYG - healthy life year gained; I$ - international dollars; UMHICs - upper middle- to high-income countries; SEL - socio-emotional learning.

Note: Percent reductions in the intervention effect size were applied equally to both the intervention effect size applied to depression/anxiety cases and the intervention effect size applied to suicide deaths.

Reference list

1. Bertram MY, Chisholm D, Watts R, Waqanivalu T, Prasad V, Varghese C. Cost-Effectiveness of Population Level and Individual Level Interventions to Combat Non-communicable Disease in Eastern Sub-Saharan Africa and South East Asia: A WHO-CHOICE Analysis. Int J Health Policy Manag 2021;10(11):724-33.

2. Bertram MY, Lauer JA, Stenberg K, Edejer TTT. Methods for the Economic Evaluation of Health Care Interventions for Priority Setting in the Health System: An Update From WHO CHOICE. Int J Health Policy Manag 2021;10(11):673-7.

3. WHO. WHO menu of cost-effective interventions for mental health [Online]. World Health Organization. 2021 [cited 20 July 2022]; Available from: https://apps.who.int/iris/handle/10665/343074

4. WHO. Comprehensive mental health action plan 2013–2030 [Online]. World Health Organization. 2013 [cited 20 July 2022]; Available from: https://apps.who.int/iris/handle/10665/345301

5. WHO. Global Action Plan for the Prevention and Control of NCDs 2013-2020 [Online]. World Health Organization. 2013 [cited 20 July 2022]; Available from: https://www.who.int/publications/i/item/9789241506236

6. Tan-Torres Edejer T, Baltussen R, Adam T, Hutubessy R, Acharya A, Evans DB, Murray CJL. Making choices in health: WHO guide to cost-effectiveness analysis [Online]. World Health Organization. 2003; Available from: https://apps.who.int/iris/handle/10665/42699

7. Avenir Health. OneHealth Tool [Online]. Avenir Health. 2022 [cited 20 July 2022]; Available from: https://www.avenirhealth.org/software-onehealth.php

8. UN DESA. World Population Prospects: The 2017 Revision [Online]. United Nations. 2017 [cited 3 August 2019]; Available from: https://www.un.org/development/desa/publications/world-population-prospects-the-2017-revision.html

9. UIS. Welcome to UIS.Stat [Online]. UNESCO Institute for Statistics. 2022 [cited 25 August 2019]; Available from: http://data.uis.unesco.org/

10. Bertha EA, Balazs J. Subthreshold depression in adolescence: a systematic review. Eur Child Adolesc Psychiatry 2013;22(10):589-603.

11. Carrellas NW, Biederman J, Uchida M. How prevalent and morbid are subthreshold manifestations of major depression in adolescents? A literature review. J Affect Disord 2017;210:166-73.

12. Haller H, Cramer H, Lauche R, Gass F, Dobos GJ. The prevalence and burden of subthreshold generalized anxiety disorder: a systematic review. BMC Psychiatry 2014;14:128.

13. Skeen S, Laurenzi CA, Gordon SL, du Toit S, Tomlinson M, Dua T, Fleischmann A, Kohl K, Ross D, Servili C, Brand AS, Dowdall N, Lund C, van der Westhuizen C, Carvajal-Aguirre L, Eriksson de Carvalho C, Melendez-Torres GJ. Adolescent Mental Health Program Components and Behavior Risk Reduction: A Meta-analysis. Pediatrics 2019;144(2).

14. Hetrick SE, Cox GR, Witt KG, Bir JJ, Merry SN. Cognitive behavioural therapy (CBT), third-wave CBT and interpersonal therapy (IPT) based interventions for preventing depression in children and adolescents. Cochrane Database Syst Rev 2016(8):CD003380.

15. Stockings EA, Degenhardt L, Dobbins T, Lee YY, Erskine HE, Whiteford HA, Patton G. Preventing depression and anxiety in young people: a review of the joint efficacy of universal, selective and indicated prevention. Psychol Med 2016;46(1):11-26.

16. Lee YY, Le LK, Stockings EA, Hay P, Whiteford HA, Barendregt JJ, Mihalopoulos C. Estimation of a relative risk effect size when using continuous outcomes data: an application of methods in the prevention of major depression and eating disorders. Med Decis Making 2018;38(7):866-80.

17. WHO. School-based interventions for reducing deaths from suicide and suicide attempts among young people [Online]. World Health Organization. 2015 [cited 20 July 2022]; Available from: https://cdn.who.int/media/docs/default-source/mental-health/mhgap/self-harm-and-suicide/school-based-interventions-for-reducing-deaths-from-suicide-and-suicide-attempts-among-young-people.pdf

18. WHO. mhGAP Evidence Resource Centre [Online]. World Health Organization. 2022 [cited 20 July 2022]; Available from: https://www.who.int/teams/mental-health-and-substance-use/treatment-care/mental-health-gap-action-programme/evidence-centre

19. IHME. Global Burden of Disease Study 2017 (GBD 2017) Data Resources [Online]. University of Washington. 2022 [cited 10 February 2022]; Available from: https://ghdx.healthdata.org/gbd-2017

20. Ferrari AJ, Norman RE, Freedman G, Baxter AJ, Pirkis JE, Harris MG, Page A, Carnahan E, Degenhardt L, Vos T, Whiteford HA. The burden attributable to mental and substance use disorders as risk factors for suicide: findings from the Global Burden of Disease Study 2010. PLoS One 2014;9(4):e91936.

21. Alonso J, Liu Z, Evans-Lacko S, Sadikova E, Sampson N, Chatterji S, Abdulmalik J, Aguilar-Gaxiola S, Al-Hamzawi A, Andrade LH, Bruffaerts R, Cardoso G, Cia A, Florescu S, de Girolamo G, Gureje O, Haro JM, He Y, de Jonge P, Karam EG, Kawakami N, Kovess-Masfety V, Lee S, Levinson D, Medina-Mora ME, Navarro-Mateu F, Pennell BE, Piazza M, Posada-Villa J, Ten Have M, Zarkov Z, Kessler RC, Thornicroft G, Collaborators WHOWMHS. Treatment gap for anxiety disorders is global: Results of the World Mental Health Surveys in 21 countries. Depress Anxiety 2018;35(3):195-208.

22. Kessler RC, Sampson NA, Berglund P, Gruber MJ, Al-Hamzawi A, Andrade L, Bunting B, Demyttenaere K, Florescu S, de Girolamo G, Gureje O, He Y, Hu C, Huang Y, Karam E, Kovess-Masfety V, Lee S, Levinson D, Medina Mora ME, Moskalewicz J, Nakamura Y, Navarro-Mateu F, Browne MA, Piazza M, Posada-Villa J, Slade T, Ten Have M, Torres Y, Vilagut G, Xavier M, Zarkov Z, Shahly V, Wilcox MA. Anxious and non-anxious major depressive disorder in the World Health Organization World Mental Health Surveys. Epidemiol Psychiatr Sci 2015;24(3):210-26.

23. Mathers CD, Iburg KM, Begg S. Adjusting for dependent comorbidity in the calculation of healthy life expectancy. Popul Health Metr 2006;4:4.

24. Bond L, Patton G, Glover S, Carlin JB, Butler H, Thomas L, Bowes G. The Gatehouse Project: can a multilevel school intervention affect emotional wellbeing and health risk behaviours? J Epidemiol Community Health 2004;58(12):997-1003.

25. Patton GC, Bond L, Carlin JB, Thomas L, Butler H, Glover S, Catalano R, Bowes G. Promoting social inclusion in schools: a group-randomized trial of effects on student health risk behavior and well-being. Am J Public Health 2006;96(9):1582-7.

26. Buttigieg JP, Shortt AL, Slaviero TM, Hutchinson D, Kremer P, Toumbourou JW. A longitudinal evaluation of the Resilient Families randomized trial to prevent early adolescent depressive symptoms. J Adolesc 2015;44:204-13.

27. Butzer B, LoRusso A, Shin SH, Khalsa SB. Evaluation of Yoga for Preventing Adolescent Substance Use Risk Factors in a Middle School Setting: A Preliminary Group-Randomized Controlled Trial. J Youth Adolesc 2017;46(3):603-32.

28. Horowitz JL, Garber J, Ciesla JA, Young JF, Mufson L. Prevention of depressive symptoms in adolescents: a randomized trial of cognitive-behavioral and interpersonal prevention programs. J Consult Clin Psychol 2007;75(5):693-706.

29. Khalsa SB, Hickey-Schultz L, Cohen D, Steiner N, Cope S. Evaluation of the mental health benefits of yoga in a secondary school: a preliminary randomized controlled trial. J Behav Health Serv Res 2012;39(1):80-90.

30. Kindt KC, Kleinjan M, Janssens JM, Scholte RH. Evaluation of a school-based depression prevention program among adolescents from low-income areas: a randomized controlled effectiveness trial. Int J Environ Res Public Health 2014;11(5):5273-93.

31. Kraag G, Van Breukelen GJ, Kok G, Hosman C. 'Learn Young, Learn Fair', a stress management program for fifth and sixth graders: longitudinal results from an experimental study. J Child Psychol Psychiatry 2009;50(9):1185-95.

32. Langer AI, Schmidt C, Aguilar-Parra JM, Cid C, Magni A. [Effects of a mindfulness intervention in Chilean high schoolers]. Rev Med Chil 2017;145(4):476-82.

33. Leventhal KS, Gillham J, DeMaria L, Andrew G, Peabody J, Leventhal S. Building psychosocial assets and wellbeing among adolescent girls: A randomized controlled trial. J Adolesc 2015;45:284-95.

34. Melnyk BM, Jacobson D, Kelly S, Belyea M, Shaibi G, Small L, O'Haver J, Marsiglia FF. Promoting healthy lifestyles in high school adolescents: a randomized controlled trial. Am J Prev Med 2013;45(4):407-15.

35. Melnyk BM, Jacobson D, Kelly SA, Belyea MJ, Shaibi GQ, Small L, O'Haver JA, Marsiglia FF. Twelve-Month Effects of the COPE Healthy Lifestyles TEEN Program on Overweight and Depressive Symptoms in High School Adolescents. J Sch Health 2015;85(12):861-70.

36. Mendelson T, Greenberg MT, Dariotis JK, Gould LF, Rhoades BL, Leaf PJ. Feasibility and preliminary outcomes of a school-based mindfulness intervention for urban youth. J Abnorm Child Psychol 2010;38(7):985-94.

37. Merry S, McDowell H, Wild CJ, Bir J, Cunliffe R. A randomized placebo-controlled trial of a school-based depression prevention program. J Am Acad Child Adolesc Psychiatry 2004;43(5):538-47.

38. Nash KA. Implementation and evaluation of the empower youth program. J Holist Nurs 2007;25(1):26-36; discussion 7-8.

39. Noggle JJ, Steiner NJ, Minami T, Khalsa SB. Benefits of yoga for psychosocial well-being in a US high school curriculum: a preliminary randomized controlled trial. J Dev Behav Pediatr 2012;33(3):193-201.

40. Park J, Kosterman R, Hawkins JD, Haggerty KP, Duncan TE, Duncan SC, Spoth R. Effects of the "Preparing for the Drug Free Years" curriculum on growth in alcohol use and risk for alcohol use in early adolescence. Prev Sci 2000;1(3):125-38.

41. Mason WA, Kosterman R, Hawkins JD, Haggerty KP, Spoth RL, Redmond C. Influence of a family-focused substance use preventive intervention on growth in adolescent depressive symptoms. J Res Adolesc 2007;17(3):541-64.

42. Possel P, Horn AB, Groen G, Hautzinger M. School-based prevention of depressive symptoms in adolescents: a 6-month follow-up. J Am Acad Child Adolesc Psychiatry 2004;43(8):1003-10.

43. Possel P, Martin NC, Garber J, Hautzinger M. A randomized controlled trial of a cognitive-behavioral program for the prevention of depression in adolescents compared with nonspecific and no-intervention control conditions. J Couns Psychol 2013;60(3):432-8.

44. Quayle D, Dziurawiec S, Roberts C, Kane R, Ebsworthy G. The effect of an optimism and lifeskills program on depressive symptoms in preadolescence. Behav Change 2001;18(4):194-203.

45. Rivet-Duval E, Heriot S, Hunt C. Preventing Adolescent Depression in Mauritius: A Universal School-Based Program. Child Adolesc Ment Health 2011;16(2):86-91.

46. Roberts CM, Kane R, Bishop B, Cross D, Fenton J, Hart B. The prevention of anxiety and depression in children from disadvantaged schools. Behav Res Ther 2010;48(1):68-73.

47. Rose K, Hawes DJ, Hunt CJ. Randomized controlled trial of a friendship skills intervention on adolescent depressive symptoms. J Consult Clin Psychol 2014;82(3):510-20.

48. Ruini C, Belaise C, Brombin C, Caffo E, Fava GA. Well-being therapy in school settings: a pilot study. Psychother Psychosom 2006;75(6):331-6.

49. Sawyer MG, Pfeiffer S, Spence SH, Bond L, Graetz B, Kay D, Patton G, Sheffield J. School-based prevention of depression: a randomised controlled study of the beyondblue schools research initiative. J Child Psychol Psychiatry 2010;51(2):199-209.

50. Sawyer MG, Harchak TF, Spence SH, Bond L, Graetz B, Kay D, Patton G, Sheffield J. School-based prevention of depression: a 2-year follow-up of a randomized controlled trial of the beyondblue schools research initiative. J Adolesc Health 2010;47(3):297-304.

51. Schilling EA, Aseltine RH, Jr., James A. The SOS Suicide Prevention Program: Further Evidence of Efficacy and Effectiveness. Prev Sci 2016;17(2):157-66.

52. Sibinga EM, Perry-Parrish C, Chung SE, Johnson SB, Smith M, Ellen JM. School-based mindfulness instruction for urban male youth: a small randomized controlled trial. Prev Med 2013;57(6):799-801.

53. Sibinga EM, Webb L, Ghazarian SR, Ellen JM. School-Based Mindfulness Instruction: An RCT. Pediatrics 2016;137(1).

54. Stallard P, Sayal K, Phillips R, Taylor JA, Spears M, Anderson R, Araya R, Lewis G, Millings A, Montgomery AA. Classroom based cognitive behavioural therapy in reducing symptoms of depression in high risk adolescents: pragmatic cluster randomised controlled trial. BMJ 2012;345:e6058.

55. Stallard P, Phillips R, Montgomery AA, Spears M, Anderson R, Taylor J, Araya R, Lewis G, Ukoumunne OC, Millings A, Georgiou L, Cook E, Sayal K. A cluster randomised controlled trial to determine the clinical effectiveness and cost-effectiveness of classroom-based cognitive-behavioural therapy (CBT) in reducing symptoms of depression in high-risk adolescents. Health Technol Assess 2013;17(47):vii-xvii, 1-109.

56. Stallard P, Skryabina E, Taylor G, Phillips R, Daniels H, Anderson R, Simpson N. Classroom-based cognitive behaviour therapy (FRIENDS): a cluster randomised controlled trial to Prevent Anxiety in Children through Education in Schools (PACES). Lancet Psychiatry 2014;1(3):185-92.

57. Stallard P, Skryabina E, Taylor G, Anderson R, Ukoumunne OC, Daniels H, Phillips R, Simpson N. A cluster randomised controlled trial comparing the effectiveness and cost-effectiveness of a school-based cognitive-behavioural therapy programme (FRIENDS) in the reduction of anxiety and improvement in mood in children aged 9/10 years. Southampton (UK); 2015. (Public Health Research, No. 3.14).

58. Tak YR, Kleinjan M, Lichtwarck-Aschoff A, Engels RC. Secondary outcomes of a school-based universal resiliency training for adolescents: a cluster randomized controlled trial. BMC Public Health 2014;14:1171.

59. Tak YR, Lichtwarck-Aschoff A, Gillham JE, Van Zundert RM, Engels RC. Universal School-Based Depression Prevention 'Op Volle Kracht': a Longitudinal Cluster Randomized Controlled Trial. J Abnorm Child Psychol 2016;44(5):949-61.

60. Arnarson EO, Craighead WE. Prevention of depression among Icelandic adolescents. Behav Res Ther 2009;47(7):577-85.

61. Arnarson EO, Craighead WE. Prevention of depression among Icelandic adolescents: a 12-month follow-up. Behav Res Ther 2011;49(3):170-4.

62. Balle M, Tortella-Feliu M. Efficacy of a brief school-based program for selective prevention of childhood anxiety. Anxiety Stress Coping 2010;23(1):71-85.

63. Bella-Awusah T, Ani C, Ajuwon A, Omigbodun O. Effectiveness of brief school-based, group cognitive behavioural therapy for depressed adolescents in south west Nigeria. Child Adolesc Ment Health 2016;21(1):44-50.

64. Berry K, Hunt CJ. Evaluation of an intervention program for anxious adolescent boys who are bullied at school. J Adolesc Health 2009;45(4):376-82.

65. Dobson KS, Ahnberg Hopkins J, Fata L, Scherrer M, Allan LC. The prevention of depression and anxiety in a sample of high-risk adolescents: a randomized controlled trial. Can J Sch Psychol 2010;25(4):291-310.

66. Gaete J, Martinez V, Fritsch R, Rojas G, Montgomery AA, Araya R. Indicated school-based intervention to improve depressive symptoms among at risk Chilean adolescents: a randomized controlled trial. BMC Psychiatry 2016;16:276.

67. Gau JM, Stice E, Rohde P, Seeley JR. Negative life events and substance use moderate cognitive behavioral adolescent depression prevention intervention. Cogn Behav Ther 2012;41(3):241-50.

68. Hunt C, Andrews G, Crino R, Erskine A, Sakashita C. Randomized controlled trial of an early intervention programme for adolescent anxiety disorders. Aust N Z J Psychiatry 2009;43(4):300-4.

69. Jacob J, De Guzman RG. Effectiveness of taking in the good based-bibliotherapy intervention program among depressed Filipino female adolescents. Asian J Psychiatr 2016;23:99-107.

70. Kwok SYCL, Gu M, Kit KTK. Positive psychology intervention to alleviate child depression and increase life satisfaction: a randomized clinical trial. Res Soc Work Pract 2016;26(4):350-61.

71. Livheim F, Hayes L, Ghaderi A, Magnusdottir T, Högfeldt A, Rowse J, Turner S, Hayes SC, Tengström A. The effectiveness of Acceptance and Commitment Therapy for adolescent mental health: Swedish and Australian pilot outcomes. J Child Fam Stud 2015;24(4):1016-30.

72. Martinsen KD, Rasmussen LMP, Wentzel-Larsen T, Holen S, Sund AM, Lovaas MES, Patras J, Kendall PC, Waaktaar T, Neumer SP. Prevention of anxiety and depression in school children: Effectiveness of the transdiagnostic EMOTION program. J Consult Clin Psychol 2019;87(2):212-9.

73. McCarty CA, Violette HD, McCauley E. Feasibility of the positive thoughts and actions prevention program for middle schoolers at risk for depression. Depress Res Treat 2011;2011:241386.

74. Noël LT, Rost K, Gromer J. Depression prevention among rural preadolescent girls: a randomized controlled trial. Sch Soc Work J 2013;38(1):1-18.

75. Poppelaars M, Tak YR, Lichtwarck-Aschoff A, Engels RC, Lobel A, Merry SN, Lucassen MF, Granic I. A randomized controlled trial comparing two cognitive-behavioral programs for adolescent girls with subclinical depression: A school-based program (Op Volle Kracht) and a computerized program (SPARX). Behav Res Ther 2016;80:33-42.

76. Puskar K, Sereika S, Tusaie-Mumford K. Effect of the Teaching Kids to Cope (TKC) program on outcomes of depression and coping among rural adolescents. J Child Adolesc Psychiatr Nurs 2003;16(2):71-80.

77. Pybis J, Cooper M, Hill A, Cromarty K, Levesley R, Murdoch J, Turner N. Pilot randomised controlled trial of school-based humanistic counselling for psychological distress in young people: outcomes and methodological reflections. Couns Psychother Res 2015;15(4):241-50.

78. Roberts C, Kane R, Thomson H, Bishop B, Hart B. The prevention of depressive symptoms in rural school children: a randomized controlled trial. J Consult Clin Psychol 2003;71(3):622-8.

79. Roberts C, Kane R, Bishop B, Matthews H, Thomson H. The prevention of depressive symptoms in rural school children: a follow-up study. Int J Ment Health Promot 2004;6(3):4-16.

80. Rohde P, Stice E, Shaw H, Briere FN. Indicated cognitive behavioral group depression prevention compared to bibliotherapy and brochure control: acute effects of an effectiveness trial with adolescents. J Consult Clin Psychol 2014;82(1):65-74.

81. Rohde P, Stice E, Shaw H, Gau JM. Effectiveness trial of an indicated cognitive-behavioral group adolescent depression prevention program versus bibliotherapy and brochure control at 1- and 2-year follow-up. J Consult Clin Psychol 2015;83(4):736-47.

82. Saelid GA, Nordahl HM. Rational emotive behaviour therapy in high schools to educate in mental health and empower youth health. A randomized controlled study of a brief intervention. Cogn Behav Ther 2017;46(3):196-210.

83. Sheffield JK, Spence SH, Rapee RM, Kowalenko N, Wignall A, Davis A, McLoone J. Evaluation of universal, indicated, and combined cognitive-behavioral approaches to the prevention of depression among adolescents. J Consult Clin Psychol 2006;74(1):66-79.

84. Smith P, Scott R, Eshkevari E, Jatta F, Leigh E, Harris V, Robinson A, Abeles P, Proudfoot J, Verduyn C, Yule W. Computerised CBT for depressed adolescents: Randomised controlled trial. Behav Res Ther 2015;73:104-10.

85. Spence SH, Sheffield JK, Donovan CL. Preventing adolescent depression: an evaluation of the problem solving for life program. J Consult Clin Psychol 2003;71(1):3-13.

86. Stasiak K, Hatcher S, Frampton C, Merry SN. A pilot double blind randomized placebo controlled trial of a prototype computer-based cognitive behavioural therapy program for adolescents with symptoms of depression. Behav Cogn Psychother 2014;42(4):385-401.

87. Wijnhoven LA, Creemers DH, Vermulst AA, Scholte RH, Engels RC. Randomized controlled trial testing the effectiveness of a depression prevention program ('Op Volle Kracht') among adolescent girls with elevated depressive symptoms. J Abnorm Child Psychol 2014;42(2):217-28.

88. Woods B, Jose PE. Effectiveness of a school-based indicated early intervention program for Māori and Pacific adolescents. J Pac Rim Psychol 2011;5(1):40-50.

89. Young JF, Mufson L, Davies M. Efficacy of Interpersonal Psychotherapy-Adolescent Skills Training: an indicated preventive intervention for depression. J Child Psychol Psychiatry 2006;47(12):1254-62.

90. Young JF, Gallop R, Mufson L. Mother-child conflict and its moderating effects on depression outcomes in a preventive intervention for adolescent depression. J Clin Child Adolesc Psychol 2009;38(5):696-704.

91. Young JF, Mufson L, Gallop R. Preventing depression: a randomized trial of interpersonal psychotherapy-adolescent skills training. Depress Anxiety 2010;27(5):426-33.

92. Young JF, Benas JS, Schueler CM, Gallop R, Gillham JE, Mufson L. A Randomized Depression Prevention Trial Comparing Interpersonal Psychotherapy--Adolescent Skills Training to Group Counseling in Schools. Prev Sci 2016;17(3):314-24.

93. Young JF, Jones JD, Sbrilli MD, Benas JS, Spiro CN, Haimm CA, Gallop R, Mufson L, Gillham JE. Long-Term Effects from a School-Based Trial Comparing Interpersonal Psychotherapy-Adolescent Skills Training to Group Counseling. J Clin Child Adolesc Psychol 2019;48(sup1):S362-S70.

94. Chinn S. A simple method for converting an odds ratio to effect size for use in meta-analysis. Stat Med 2000;19(22):3127-31.

95. Hasselblad V, Hedges LV. Meta-analysis of screening and diagnostic tests. Psychol Bull 1995;117(1):167-78.

96. Briggs AH, Weinstein MC, Fenwick EA, Karnon J, Sculpher MJ, Paltiel AD, Force I-SMGRPT. Model parameter estimation and uncertainty: a report of the ISPOR-SMDM Modeling Good Research Practices Task Force--6. Value Health 2012;15(6):835-42.

97. WHO. Scaling up action against noncommunicable diseases: how much will it cost? [Online]. World Health Organization. 2011 [cited 20 July 2022]; Available from: https://apps.who.int/iris/handle/10665/44706

98. WHO. Tackling NCDs: "Best buys" and other recommended interventions for the prevention and control of noncommunicable diseases [Online]. World Health Organization. 2017 [cited 20 July 2022]; Available from: https://apps.who.int/iris/handle/10665/259232

99. Stenberg K, Lauer JA, Gkountouras G, Fitzpatrick C, Stanciole A. Econometric estimation of WHO-CHOICE country-specific costs for inpatient and outpatient health service delivery. Cost Eff Resour Alloc 2018;16:11.

100. World Bank. World Bank Open Data [Online]. The World Bank Group. 2019 [cited 25 August 2019]; Available from: https://data.worldbank.org/
